# Phylogeny of *Geoglossomycetes* with species diversity in China

**DOI:** 10.1080/21501203.2024.2436000

**Published:** 2025-02-03

**Authors:** Hongli Su, Kevin D. Hyde, Qing Cai, Wenyan Chen, Fatimah Al-Otibi, K.W. Thilini Chethana, Zhu L. Yang, Qi Zhao

**Affiliations:** a Key Laboratory of Phytochemistry and Natural Medicines, Chinese Academy of Sciences, Kunming, China; b Center of Excellence in Fungal Research, Mae Fah Luang University, Chiang Rai, Thailand; c School of Science, Mae Fah Luang University, Chiang Rai, Thailand; d Department of Botany and Microbiology, College of Science, King Saud University, Riyadh, Saudi Arabia

**Keywords:** 10 new taxa, earth tongue, *Geoglossaceae*, morphology, taxonomy

## Abstract

*Geoglossomycetes* is a class within the phylum Ascomycota that accommodates a single order and a single family, comprising nine genera. *Geoglossomycetes* is traditionally referred to as “earth tongues”. The class is characterised by tongue-shaped to clavate, stipitate, black ascomata covered with or without black setae, a swollen ascigerous portion, a cylindric stipe, filiform, septate paraphyses, cylindrical-clavate, 4–8-spored asci, and filiform or falciform, multi-septate, dark brown to hyaline ascospores. In this study, we examined 34 samples from four genera in China. Based on ecological comparisons, morphological studies, and phylogenetic analyses inferred from the combined internal transcribed spacer (ITS) regions and the large subunit of the ribosomal RNA gene (LSU), we introduce ten new species of *Geoglossomycetes*, and identify four known species. In addition, we summarise the sexual morph characters of all species within the two largest genera, *Geoglossum* and *Trichoglossum*.

## 1. Introduction

### 1.1. *Introduction of* Geoglossomycetes

*Geoglossomycetes* Zheng Wang, C.L. Schoch & Spatafora is a common terrestrial class of Ascomycota with *Geoglossales* as its type order. *Geoglossales* only accommodates one family, *Geoglossaceae* (Schoch et al. 2009; Wijayawardene et al. 2022). The class is characterised by large, tongue-shaped, claviform or capitate, stipitate, brown to black ascomata with or without acicular, brown to black setae, some taxa covered by a thick gel layer, terete, lanceolate, ellipsoid or fusoid ascigerous portion, brown to black stipe, some taxa with squamules, filiform, septate, brown to hyaline paraphyses with frequently swollen, usually curved apex, cylindrical-clavate, 4–8-spored asci with mostly amyloid, rarely inamyloid apex, and filiform, claviform, vermiform, falciform, straight to obviously curved, septate, dark brown to hyaline ascospores (Hustad et al. 2013; Jaklitsch et al. 2016).

### 1.2. *History of* Geoglossomycetes

*Geoglossaceae* was erected by Corda (1838) to accommodate *Geoglossum* (*Ge*.), and the family was originally diagnosed by club-shaped, stalked, fleshy fruiting body with smooth or hairy surface, submarginal hymenium bearing asci, compound, septate or cellular ascospores. Bonorden (1851), Bail (1858), Gillet (1879), and Boudier (1885) accepted the existence of *Geoglossaceae* and placed it in *Discomycetes* based on its macroscopic features. However, the family rank of *Geoglossaceae* was questioned by Fries (1846), Cooke (1871, 1875), Saccardo (1884), and Quélet (1886), who treated *Geoglossum* as a genus of *Helvellaceae* (previously known as *Elvellacei*, *Helvellacei*, *Helvelleae*, and *Helvelei*), based on its clavate and immarginate ascomata similar to those of the members of *Helvellaceae*. Saccardo (1889) divided the traditional *Geoglossum* group into two families based on the morphologies and the gelatinous consistency of their ascomata; one was *Helvelleae* with vertical fruiting bodies, and the other was *Bulgarieae* with their gelatinous fruiting bodies. Boudier (1907) categorised *Discomycetes* into two groups, Inopercules and Opercules, based on the apical dehiscence of their asci. *Geoglossaceae* belonged to Inopercules, while *Helvellaceae* belonged to Opercules, thus completely separating *Geoglossaceae* from *Helvellaceae*.

*Helotiales* was established by Nannfeldt (1932), and *Geoglossaceae* was identified as a family in *Helotiales* based on the characteristics of asci, ascospores, excipular, and other sterile structures (Nannfeldt 1932). This classification was endorsed by Nannfeldt (1942), Imai (1940, 1941, 1956), Korf (1973), and Dennis (1978). By this time, the taxonomic status of *Geoglossaceae* was clear, but its genera were confused due to differing taxonomic criteria used by Nannfeldt (1932, 1942), Imai (1940, 1941). Nannfeldt (1932, 1942), Imai (1956), and Raitviir (1971, 1991) questioned whether *Geoglossaceae* was morphologically polyphyletic, leading them to make numerous revisions to the classification and scope of *Geoglossaceae*.

Imai (1956) proposed a classification system for *Geoglossaceae* based primarily on the shape of the ascospores and the fleshy ascocarps covered by a gelatinous consistency, with gross morphology playing a smaller role. In his system, *Geoglossaceae* was divided into three subfamilies, eleven tribes, and nineteen genera. However, this classification was opposed by Raitviir (1971), who argued that Imai’s subfamilies were equivalent to commonly accepted tribes, and Imai’s tribes were equivalent to commonly accepted genera. As a result, many genera were placed within *Geoglossaceae*. Korf (1958) transferred the subfamily *Leotioideae* from *Geoglossaceae* to *Helotiaceae* based on the similar morphology of *Leotia* and *Neocudoniella*. Korf’s (1958) research laid the foundation for the modern classification of *Geoglossaceae*, which was endorsed by Kimbrough (1970), Korf (1973), Dennis (1978), and Spooner (1987). They accepted that *Geoglossaceae*, in the modern sense, was divided into two families: *Geoglossaceae* and *Helotiaceae*. However, the precise delimitation of *Geoglossaceae* was controversial; only three core genera (*Geoglossum*, *Microglossum*, and *Trichoglossum*) were universally acknowledged, while many other genera had contentious relationships with *Geoglossaceae* (Platt 1999; Lutzoni et al. 2004; Sandnes 2006; Wang et al. 2006a, 2006b; Schoch et al. 2009; Hustad et al. 2011, 2013, 2015; Arauzo and Iglesias 2014; Fedosova 2019).

As phylogenetics was applied to taxonomy, the generic circumscriptions of *Geoglossaceae* became clearer based on monophyly (Wheeler and Meier 2000; Sites and Marshall 2004). Platt (1999), Sandnes (2006), and Wang et al. (2006a, 2006b) used their phylogenies to propose that the modern concept of *Geoglossaceae* should include three genera (*Geoglossum*, *Sarcoleotia*, and *Trichoglossum*) while excluding some highly contentious genera (*Bryoglossum*, *Cudonia*, *Leotia*, *Microglossum*, *Mitrula*, and *Spathularia*), as well as one core genus (*Microglossum*). Furthermore, these phylogenies by Wang et al. (2006a, 2006b) demonstrated that two genera (*Geoglossum* and *Trichoglossum*) of *Geoglossaceae* formed a distinct monophyletic clade separated from *Leotiomycetes*. This phylogenetic affiliation with *Geoglossaceae* and *Leotiomycetes* was consistent with findings by Lutzoni et al. (2004). Therefore, *Geoglossaceae* was moved out of *Leotiomycetes* to become incertae sedis within *Pezizomycotina* in Kirk et al.’s (2008) “Ainsworth & Bisby’s Dictionary of the Fungi”. Based on the independent phylogenetic placement of *Geoglossaceae* within Ascomycota as shown by Lutzoni et al. (2004) and Wang et al. (2006a, 2006b), Schoch et al. (2009) reconstructed the phylogeny of Ascomycota, establishing *Geoglossomycetes* and *Geoglossales* with *Geoglossaceae* as the type family. Their phylogenetic tree confirmed that *Geoglossum*, *Trichoglossum*, and *Sarcoleotia* belonged to *Geoglossaceae*, while *Bryoglossum*, *Cudonia*, *Microglossum*, *Mitrula*, *Neolecta*, *Spathularia*, and *Thuemenidum* were excluded from *Geoglossaceae*.

As the molecular data increased, more and more genera were assigned to *Geoglossomycetes*. Hustad et al. (2011) explained that *Nothomitra* was a well-supported genus within *Geoglossomycetes* based on a combination of morphological characteristics and phylogeny of ITS, LSU, the minichromosome maintenance complex component 7 protein-coding gene (*MCM7*). Hustad et al. (2013) combined morphology and multi-gene phylogeny combined ITS, LSU, *MCM7*, and the largest subunit of RNA polymerase II gene sequences (*RPB1*) gene regions to clarify the generic circumscriptions of *Geoglossomycetes*, proposed *Glutinoglossum* (*Gl*.) and *Sabuloglossum* within *Geoglossomycetes*. Arauzo and Iglesias (2014) used *Trichoglossum leucosporum* to recombine *Leucoglossum*，and established *Hemileucoglossum* based on a combination of the ITS phylogeny of *Geoglossaceae* and morphological characteristics. Hustad and Miller (2015a) used morphology, ecology, and phylogeny based on the combined dataset of ITS and LSU to transfer *Geoglossum aseptatum* to *Maasoglossum*. Up to now, *Geoglossomycetes* includes one order (*Geoglossales*), one family (*Geoglossaceae*), and nine genera (*Geoglossum*, *Glutinoglossum*, *Hemileucoglossum*, *Leucoglossum*, *Maasoglossum*, *Nothomitra*, *Sabuloglossum*, *Sarcoleotia*, and *Trichoglossum*) (Fedosova 2019).

### 1.3. *Chinese study of* Geoglossaceae

The study of *Geoglossaceae* in China began in the late 19th century. Kalchbrenner and Thümen (1881) and Patouillard (1890, 1893) classified and described the Chinese samples of *Spathularia flavida*, *Microglossum partitum*, and *Trichoglossum hirsutum*. Additionally, Patouillard (1890) established *Hemiglossum* based on a fungus collected from Yunnan, China. Subsequently, more mycologists focused on *Geoglossaceae* in China. Teng (1932, 1934, 1939) and Tai (1937) studied fungi in China, mentioning *Geoglossaceae* in their work. Later, Tai (1944) published the study on the *Geoglossaceae* in Yunnan, China, in which he recorded twenty-nine species and four varieties, including twelve species and three varieties described as new. He also guessed that Yunnan seemed to possess a *Geoglossaceae* flora of exceptional richness. Subsequently, Teng (1963) and Tai (1979) each compiled studies on fungi from China, providing detailed and comprehensive research on *Geoglossaceae*. Zang (1983) complemented the species diversity of *Geoglossaceae* in Tibet. Zhuang and Wang (1997, 1998) revisited the *Discomycetes* of China, adding two new species (*Geoglossum laccatum* and *T. qingchengense*), one new variety (*T. hirsutum* var. *latisporum*), and four new geographical records. Additionally, Zhuang (1998) completed the “Flora of China Fungi”, which provides a comprehensive and detailed study of *Geoglossaceae* in China. Later, phylogenetics was applied to the taxonomy of *Geoglossaceae* (Platt 1999; Wheeler and Meier 2000; Sites and Marshall 2004; Sandnes 2006; Wang et al. 2006a, 2006b). Wang et al. (2011) published extensive molecular data from Chinese specimens of *Geoglossaceae*, laying the foundation for subsequent fungal taxonomy research. Hustad (2015) completed “A circumscription of the earth tongue fungi class *Geoglossomycetes*” based on some Chinese samples. Ekanayaka et al. (2017a) also used some Chinese specimens of *Geoglossaceae* to study *Discomycetes*. Recently, Zhuang et al. (2020) assessed the threat status of non-lichenised macro-ascomycetes in China, noting that tweleve species of *Geoglossaceae* face varying degrees of environmental threat.

In this study, 34 samples were collected from China. Based on the combination of ecological comparisons, morphological characteristics, and phylogenetic analyses using ITS and LSU, we propose ten new species. They include four new species of *Geoglossum*, one new species of *Glutinoglossum*, and five new species of *Trichoglossum*. Furthermore, we identified four known species: *Geoglossum cookeanum*, *Leucoglossum durandii*, *Trichoglossum hirsutum*, and *T. rasum*. Additionally, we summarised the sexual morphological characters of all species within *Geoglossum* and *Trichoglossum*.

## 2. Materials and methods

### 2.1. Sample collection and deposition

The thirty four specimens were newly collected in Yunnan, China. We found these collections in the wild, photographed each specimen, and recorded their sampling information, including macro-morphological descriptions, locations, and habitats. The specimens were individually wrapped in tissues and placed into separate clean plastic boxes. They were then transported to the lab and air-dried at approximately 30 °C in an electric dehydrator. After studying their morphology and phylogeny, the specimens were deposited in the Herbarium of Cryptogams at the Kunming Institute of Botany, Chinese Academy of Sciences (HKAS). Facesoffungi and Index Fungorum were obtained as in Jayasiri et al. (2015) and Index Fungorum (2024).

### 2.2. Morphological study

Morphological studies comprised two parts: macroscopic and microscopic observations. Macroscopic observations were based on field notes of fresh collections, photographs, and examinations of dried samples. Microscopic studies involved free-hand sections of dried specimens prepared using a C-PSN stereomicroscope (Nikon, Japan). Microscopic morphology was photographed and measured under water using an EOS 70D (W) digital camera (Canon, Japan) mounted to an ECLIPSE Ni-U compound microscope (Nikon, Japan) and Image Frame Work (Tarosoft, Thailand). Measurements were recorded as (a–)b–c(–d), where a denotes the minimum value, d is the maximum value, and b–c the 90% confidence interval. The average value of measurements is indicated by $\overset{ˉ}{x}$. Ascospore measurements were given as [n/m/p], indicating that n number of ascospores were measured from m ascomata of p collections. The length-to-width ratios of the ascospores were recorded as the Q value, while ***Q*** represents the average of Q for all ascospores ± standard deviation (Calatayud et al. 2002). The amyloid reaction of asci was checked using free-hand sections mounted in Melzer’s reagent with 3% KOH pre-treatment, following Korf (1958). All photographs were adjusted and compiled using Adobe Photoshop 2020 (Adobe, USA).

### 2.3. DNA extraction, PCR amplification, and sequencing

Genomic DNA of each specimen was extracted using a TSP101 DNA extraction kit (TSINGKE, China). Internal transcribed spacer (ITS) and large subunit rRNA (LSU) regions were amplified for subsequent phylogenetic analysis following protocols outlined by Hustad and Miller (2015a), Fadnes et al. (2021), Kučera et al. (2021), and de la Fuente et al. (2022). The primers used were ITS1/ITS4 (White et al. 1990) for ITS, and JS1 (Landvik 1996)/LR6 (Vilgalys and Hester 1990) for LSU. The PCR reactions were carried out in a final volume of 25 μL, consisting of 12.5 μL 2× PCR G013 Taq MasterMix with Dye (abm, Canada), 1 μL of each primer (10 μmol/L), 2 μL genomic DNA, and the remaining volume with sterilised, distilled water. The PCR process included an initial denaturation at 95 °C for 5 min, followed by 35 cycles of denaturation at 95 °C for 20 s, annealing at 53 °C for 10 s, elongation at 72 °C for 20 s, and a final elongation at 72 °C for 7 min. The PCR products were checked on 1% agarose gels stained with TSJ003 GoldView (TSINGKE, China) and observed under UV light. Successful PCR products were sequenced at TSINGKE Biotech (China).

### 2.4. Phylogenetic analyses and recombination analyses

The chromatograms of each sequence were checked using BioEdit v. 7.0.9 (Hall et al. 2011). Corresponding forward and reverse sequences were concatenated in DNASTAR Lasergene SeqMan Pro v. 7.1.0 (44.1) (Swindell and Plasterer 1997). These concatenated sequences were then searched in BLASTn against GenBank records to identify close relatives (Altschul et al. 1990). Newly generated sequences and the reference sequences from previous studies were obtained from GenBank and are listed in Table 1 (Arauzo and Iglesias 2014; Hustad and Miller 2015a; Fadnes et al. 2021; Kučera et al. 2021; de la Fuente et al. 2022). *Laetisaria arvalis* (CBS 131.82), *Marchandiomyces marsonii* (ATCC MYA-4210), *Mycotribulus mirabilis* (CPC 14167), and *Chaetospermum chaetosporum* (CBS 154.59) were used as the outgroup taxa.

**Table 1.** GenBank accession numbers for taxa used in this study.

Species name	Voucher/strain number	ITS	LSU	References
*Archaeorhizomyces finlayi*-**T**	CBS 128710	NR_121541	NG_064244	Ihrmark et al. (2012)
*Arthroderma curreyi*	CBS 130.70	AJ877223	AY176726	Brasch and Gräser (2005)
*Ascosphaera apis*	CBS 402.96	FJ172292	FJ358275	Chen et al. (2018)
*Candelaria pacifica*-**T**	Westberg 953 (LD)	GU929919	KP794965	Schneider et al. (2015)
*Chaenotheca furfuracea*-**T**	UPS Wedin 6366	NR_120128	JX000087	Schoch et al. (2014)
*Chaetospermum chaetosporum*	CBS 154.59	KJ710461	KJ710439	Crous et al. (2014)
*Ctenomyces serratus*	CBS 187.61	AJ877222	FJ358282	Brasch and Gräser (2005)
*Dothidea sambuci*-**T**	AFTOL-ID 274	NR_111220	NG_027611	Schoch et al. (2014)
*Endoconidioma populi*-**T**	UAMH 10297	NR_121303	NG_059198	Schoch et al. (2014)
*Eremascus albus*	CBS 239.50	U18359	AY004345	Lumbsch et al. (2000)
***Geoglossum ailaoense*****-T**	**HKAS 136945**	**PQ306506**	**PQ196608**	**This study**
***Ge. ailaoense***	**HKAS 136946**	**PQ306507**	**PQ196609**	**This study**
*Ge. azoricum*-**T**	AMI-SPL1247	OQ618223	OQ618224	Crous et al. (2023)
*Ge. azoricum*	AMI-SPL1264	OQ617305	-	Crous et al. (2023)
*Ge. barlae*	ILLS 61034	JQ256416	JQ256433	Hustad et al. (2011)
*Ge. brunneipes*-**T**	AH 44217	NR_132093	-	Schneider et al. (2015)
*Ge. brunneipes*	ERRO 2014012201	KP144091	-	Arauzo and Iglesias (2014)
*Ge. chamaecyparinum*	ERRO 2013013001	KP144100	-	Schneider et al. (2015)
*Ge. chamaecyparinum*-**T**	AH 44219	NR_132095	-	Schneider et al. (2015)
***Ge. cookeanum***	**HKAS 112838**	**PQ306509**	**PQ196611**	**This study**
*Ge. cookeanum*	ILLS 61035	JQ256417	JQ256434	Hustad et al. (2011)
*Ge. cookeanum*	ILLS 67347	KC222122	KC222135	Hustad et al. (2013)
*Ge. cookeanum*	PDD 76527	HQ222873	-	Wang et al. (2011)
*Ge. dunense*-**T**	TUR-A 199830	NR_137971	KP744517	Loizides et al. (2015)
*Ge. dunense*	TUR-A 203150	KP744515	-	Loizides et al. (2015)
*Ge. fallax*	1131046	AY789311	-	Wang et al. (2005)
*Ge. fallax*	ERRO 2010121202	KP144110	-	Schneider et al. (2015)
*Ge. fallax*	ILLS 61037	JQ256419	JQ256435	Hustad et al. (2011)
*Ge. geesterani*-**T**	AH 44218	NR_132092	-	Schneider et al. (2015)
*Ge. geesterani*	ERRO 2011011501	KP144084	-	Schneider et al. (2015)
*Ge. glabrum*	ILLS 61038	JQ256420	JQ256436	Hustad et al. (2011)
*Ge. glabrum*	SAV 10162	KJ152695	KJ152696	Hustad et al. (2014)
*Ge. glabrum*	ILLS 72358	KP657559	-	Hustad and Miller (2015a)
*Ge. glabrum*	OSC 60610	AY789318	AY789317	Wang et al. (2005)
*Ge. heuflerianum*	Ueli Graf 25.08.2013/1	KP742955	-	Ekanayaka et al. (2017b)
*Ge. inflatum*	ERRO 2011012004	KP144102	-	Schneider et al. (2015)
*Ge. jirinae*-**T**	SAV F-11578	MT940893	MT940893	Crous et al. (2020a)
*Ge. laurisilvae*-**T**	AMI-SPL642	OM691497	OM691457	Crous et al. (2022)
*Ge. laurisilvae*	ERRO2018112301	OM691496	-	Crous et al. (2022)
***Ge. lijiangense*****-T**	**HKAS 69728**	**PQ306510**	-	**This study**
***Ge. lijiangense***	**HKAS 137589**	**PQ306511**	-	**This study**
*Ge. peckianum*	ILLS 61036	JQ256418	-	Hustad et al. (2011)
*Ge. peckianum*	ILLS 67348	KC222123	KC222136	Hustad et al. (2013)
*Ge. peckianum*	ILLS 67349	KC222124	KC222137	Hustad et al. (2013)
*Ge. pseudoumbratile*	ERRO 2014011506	KP144087	-	Arauzo and Iglesias (2014)
*Ge. pseudoumbratile*	ERRO 2009122201	KP144086	-	Arauzo and Iglesias (2014)
*Ge. pseudoumbratile*	ERRO 2012121001	KP144085	-	Arauzo and Iglesias (2014)
*Ge. pygmaeum*	ERRO 2013112415	KP144104	-	Schneider et al. (2015)
*Ge. raitviirii*-**T**	LE 303983	NR_185522	NG_228746	Crous et al. (2016)
*Ge. raitviirii*	LE 291814	KT936309	-	Crous et al. (2016)
*Ge. scabripes*-**T**	AH 44220	NR_132094	-	Schneider et al. (2015)
*Ge. simile*-**T**	CORT 005220	NR_171796	NG_073596	Hustad et al. (2014)
*Ge. simile*	ILLS 67350	KC222125	KC222138	Hustad et al. (2013)
*Ge. simile*	ILLS 61039	JQ256421	JQ256437	Hustad et al. (2011)
*Ge. glabrum*	ILLS 67351	KC222126	KC222139	Hustad et al. (2013)
***Ge. tetrasporum*****-T**	**HKAS 136947**	**PQ306508**	**PQ196610**	**This study**
*Ge. umbratile*	AFTOL-ID 56	DQ491490	AY544650	Baral and Haelewaters (2015)
*Ge. umbratile*	ERRO 2008092701	KP144097	-	Schneider et al. (2015)
*Ge. umbratile*	ILLS 61040	JQ256422	JQ256438	Hustad et al. (2011)
*Ge. umbratile*	RBG Kew K(M)64699	EU784258	-	Brock et al. (2009)
*Ge. variabilisporum*-**T**	AH 44216	NR_132096	-	Schneider et al. (2015)
*Ge. vleugelianum*	ERRO 2010120702	KP144103	-	Schneider et al. (2015)
***Ge. yuxiense*****-T**	**HKAS 137590**	**PQ306513**	**PQ196613**	**This study**
***Ge. yuxiense***	**HKAS 137588**	**PQ306512**	**PQ196612**	**This study**
*Glutinoglossum americanum*	ILLS 64444	KP690086	KP690098	Hustad and Miller (2015b)
*Gl. americanum*	ILLS 67352	KC222128	KC222141	Hustad et al. (2013)
*Gl. australasicum*-**T**	PDD 103623	KP690088	KP690100	Hustad and Miller (2015b)
*Gl. australasicum*	PDD 103619	KP690087	KP690099	Hustad and Miller (2015b)
*Gl. circinatum*	LE 303993	KX694149	KX694187	Fedosova et al. (2018)
*Gl. decorum-***T**	AH49358	OP610058	-	Sierra et al. (2023)
*Gl. decorum*	AH49356	MW901452	OP714523	Sierra et al. (2023)
*Gl. exiguum*-**T**	PDD 103574	KP690089	KP690101	Hustad and Miller (2015b)
*Gl. exiguum*	PDD 103611	KP690090	KP690102	Hustad and Miller (2015b)
*Gl. glutinosum*-**T**	LE 222165	KX694157	KX694196	Fedosova et al. (2018)
*Gl. glutinosum*	ILLS 67353	KC222129	KC222142	Hustad et al. (2013)
*Gl. heptaseptatum*	ILLS 67354	KC222130	KC222143	Hustad et al. (2013)
*Gl. heptaseptatum*	K(M) 165359	KC222131	KC222144	Hustad et al. (2013)
*Gl. heptaseptatum*	LE 222169	KX694162	KX694200	Fedosova et al. (2018)
*Gl. lumbricale*-**T**	LE<RUS>:303987	NR_158499	KX694202	Fedosova et al. (2018)
*Gl. methvenii*-**T**	PDD 103629	KP690096	KP690108	Hustad and Miller (2015b)
*Gl. methvenii*	PDD 103597	KP690095	KP690107	Hustad and Miller (2015b)
*Gl. orientale*-**T**	LE<RUS>:222166	NR_158500	NG_060681	Fedosova et al. (2018)
*Gl. orientale*	LE 291818	KX694167	KX694204	Fedosova et al. (2018)
*Gl. peregrinans*-**T**	LE<RUS>:303988	NR_158501	KX694207	Fedosova et al. (2018)
*Gl. peregrinans*	SAV F-10789	KX694168	KX694205	Fedosova et al. (2018)
*Gl. persoonii*-**T**	MCVE 31360	NR_172556	MW901459	Saitta et al. (2021)
*Gl. persoonii*	SAV F-11599	MW901457	MW901462	Kučera et al. (2022)
*Gl. proliferatum*-**T**	SAV F-11249	KX694175	KX694212	Fedosova et al. (2018)
*Gl. pseudoglutinosum*-**T**	SAV F-10903	KX694178	KX694215	Fedosova et al. (2018)
*Gl. pseudoglutinosum*	SAV F-10399	KX694183	KX694221	Fedosova et al. (2018)
***Gl.*** **sp.**	**HKAS 112858**	**PQ638543**	**PQ637362**	**This study**
*Gl. triseptatum*-**T**	SAV F-9828	KX694185	KX694223	Fedosova et al. (2018)
*Gl. triseptatum*	SAV F-10262	KX694186	KX694224	Fedosova et al. (2018)
***Gl. yunnanense*****-T**	**HKAS 112840**	**PQ306488**	**PQ196592**	**This study**
***Gl. yunnanense***	**HKAS 112861**	**PQ306487**	**PQ196591**	**This study**
*Hemileucoglossum alveolatum*	LE 291805	MF353087	-	Crous et al. (2017)
*Hemileucoglossum alveolatum*	MICH s.n.	KP657560	KP657565	Hustad and Miller (2015a)
*Hemileucoglossum kelabitense*-**T**	SAR MS0684	NR_173910	NG_088117	Crous et al. (2020b)
*Hemileucoglossum littorale*	C 35673	KP657561	-	Hustad and Miller (2015a)
*Hemileucoglossum littorale*	SAV F-10486	MF353089	MF353092	Crous et al. (2017b)
*Hemileucoglossum pusillum*-**T**	SAV F-11293	NR_158524	NG_060706	Crous et al. (2017)
*Hemileucoglossum pusillum*	O-F-257329	MW295710	MW295713	Fadnes et al. (2021)
*Laboulbenia bruchii*	D. Haelew. 1346b	OR680724	MN394843	Van Caenegem et al. (2023)
*Laboulbenia collae*	D. Haelew. 1456b	MN394845	MN394844	Haelewaters et al. (2019)
*Laboulbenia flagellata*	D. Haelew 1454b	MN397133	MN394850	Haelewaters et al. (2019)
*Laetisaria arvalis*	CBS 131.82	MH861489	MH873229	Vu et al. (2019)
*Leotia lubrica*	AFTOL-ID 1	DQ491484	AY544644	Senanayake et al. (2016)
***Leucoglossum durandii***	**HKAS 112842**	**PQ306515**	**PQ196615**	**This study**
***Leucoglossum durandii***	**HKAS 137598**	**PQ306514**	**PQ196614**	**This study**
*Leucoglossum durandii*	HMAS70090	HQ222875	-	Wang et al. (2011)
*Leucoglossum leucosporum*-**T**	B 70 0015491	NR_171218	-	Fedosova and Kovalenko (2015)
*Leucoglossum leucosporum*	LE<RUS>:291891	KP272112	KP272113	Fedosova and Kovalenko (2015)
*Maasoglossum aseptatum*	UPS F-118883	KP657562	KP657567	Hustad and Miller (2015a)
*Maasoglossum verrucisporum*	CUP-IN-000606	KP657563	KP657568	Hustad and Miller (2015a)
*Marchandiomyces marsonii*-**T**	ATCC MYA-4210	NR_164214	NG_059434	Lawrey et al. (2008)
*Microascus longirostris*-**T**	CBS 196.61	NR_132945	NG_058479	Daros-Pawlyta and Pawlyta (2023)
*Microglossum viride*-**T**	SAV 10249	NR_132026	NG_060291	Kučera et al. (2014)
*Mycotribulus mirabilis*-**T**	CPC 14167	KJ710481	KJ710456	Crous et al. (2014)
*Neodothiora populina*-**T**	CBS 147087	NR_172000	MW175405	Crous et al. (2020a)
*Neolecta vitellina*	MCVE 28969	KU725995	KU725997	Sadrati (2021)
*Nothomitra cinnamomea*	ILLS:61042	JQ256424	JQ256439	Hustad et al. (2011)
*Novakomyces olei*-**T**	NCAIM Y.02187	NR_172188	-	Čadež et al. (2021)
*Orbilia xanthostigma*	HMAS 287211	OQ534216	OQ534502	Genbank (2024)
*Peziza vesiculosa*	AFTOL-ID 507	DQ491509	DQ470948	Zhou et al. (2018)
*Sabuloglossum arenarium*	ILLS 61043	JQ256426	JQ256440	Hustad et al. (2011)
*Sabuloglossum arenarium*	OULU-F077201	GU324765	GU324764	Ohenoja et al. (2010)
*Sabuloglossum monticola*	HR_94300	MW471103	MW471101	Kučera et al. (2022)
*Sabuloglossum monticola*	SAV F-11291	MW471105	MW471102	Kučera et al. (2022)
*Sabuloglossum monticola*	SAV F-11594	MW471106	-	Kučera et al. (2022)
*Saccharomyces cerevisiae*-**T**	CBS 1171	NR_111007	NG_042623	Sugita et al. (1999)
*Sarcoleotia globosa*	MBH52476	AY789429	AY789428	Wang et al. (2005)
*Sarcoleotia globosa*	OSC 63633	AY789410	AY789409	Wang et al. (2005)
*Schizosaccharomyces pombe*	NRRL Y-12796	-	NG_042649	Kurtzman and Robnett (2013)
*Sordaria fimicola*	CBS 723.96	MH862606	MH874231	Vu et al. (2019)
*Taphrina americana*-**T**	CBS 331.55	NR_155874	MH869037	Rodrigues and Fonseca (2003)
*Taphrina antarctica*-**T**	CCFEE 5198	NR_132870	NG_059477	Nutaratat et al. (2022)
*Taphrina caerulescens*-**T**	CBS 351.35	NR_155875	NG_057691	Rodrigues and Fonseca (2003)
*Thuemenidium atropurpureum*	ILLS 61044	JQ256427	JQ256441	Hustad et al. (2011)
*Trichoglossum ailaoenses*-**T**	HKAS 124482	OP538029	-	Hyde et al. (2023)
*T. ailaoenses*	HKAS 124481	OP538028	-	Hyde et al. (2023)
*T. benghalense*-**T**	CUH AM698	MT573336	-	Chakraborty et al. (2022)
*T. benghalense*	SCC 2	MT622541	-	Chakraborty et al. (2022)
*T. beninense*-**T**	BR5020149821538	OP355329	OP355337	Senanayake et al. (2023)
*T. beninense*	BR5020163771352	OP355330	-	Senanayake et al. (2023)
*T. caespitosum*	JF-208-ITCV	-	OM727118	de la Fuente et al. (2022)
***T. chuxiongense*****-T**	**HKAS 137587**	**PQ306489**	**PQ196593**	**This study**
***T. chuxiongense***	**HKAS 137599**	**PQ306490**	**PQ196594**	**This study**
***T. conica*****-T**	**HKAS 112867**	**PQ306501**	**PQ196603**	**This study**
***T. conica***	**HKAS 112841**	**PQ306502**	**PQ196604**	**This study**
***T. distortus*****-T**	**HKAS 112863**	**PQ306499**	**PQ196601**	**This study**
***T. distortus***	**HKAS 137594**	**PQ306500**	**PQ196602**	**This study**
*T. farlowii*	ZW-personal collection	HQ222862	-	Wang et al. (2011)
***T. hirsutum***	**HKAS 137586**	**PQ306503**	**PQ196605**	**This study**
***T. hirsutum***	**HKAS 137593**	**PQ306505**	**PQ196607**	**This study**
***T. hirsutum***	**HKAS 137595**	**PQ306504**	**PQ196606**	**This study**
*T. hirsutum*	HKAS 55133	KC222133	KC222146	Hustad et al. (2013)
*T. hirsutum*	ILLS 61045	JQ256428	JQ256442	Hustad et al. (2011)
*T. hirsutum*	ILLS 67355	KC222132	KC222145	Hustad et al. (2013)
*T. hirsutum*	Jamie Platt JP267	-	AY640976	Reeb et al. (2004)
*T. hirsutum*	OSC 61726	AY789314	AY789313	Wang et al. (2005)
*T. jejuense*-**T**	JBRI-M19-946	NR_176737	-	Lee et al. (2021)
*T. octopartitum*	ILLS 61046	JQ256429	JQ256443	Hustad et al. (2011)
*T. octopartitum*	ILLS 67356	KC222134	KC222147	Hustad et al. (2013)
*T. rasum*	HCIO 52051	KY457226	KY457227	Prabhugaonkar and Pratibha (2017)
***T. rasum***	**HKAS 98131**	**PQ306491**	**PQ196595**	**This study**
***T. rasum***	**HKAS 112834**	**PQ638539**	**PQ637358**	**This study**
***T. rasum***	**HKAS 112836**	**PQ638540**	**PQ637359**	**This study**
***T. rasum***	**HKAS 112847**	**PQ638541**	**PQ637360**	**This study**
***T. rasum***	**HKAS 112849**	**PQ306492**	**PQ638538**	**This study**
***T. rasum***	**HKAS 112853**	**PQ306493**	**PQ196596**	**This study**
***T. rasum***	**HKAS 112854**	**PQ638542**	**PQ637361**	**This study**
***T. ruiliense*****-T**	**HKAS 126728**	**PQ306494**	**PQ196597**	**This study**
*T. septatum*-**T**	MFLU 17-0283	NR_160461	-	Ekanayaka et al. (2017b)
***T. subhirsutum*****-T**	**HKAS 112832**	**PQ306497**	**PQ196600**	**This study**
***T. subhirsutum***	**HKAS 69042**	**PQ306498**	-	**This study**
***T. subhirsutum***	**HKAS 112850**	**PQ306496**	**PQ196599**	**This study**
***T. subhirsutum***	**HKAS 137597**	**PQ306495**	**PQ196598**	**This study**
*T. tropicale*	JF-526-ITCV	-	OM727119	de la Fuente et al. (2022)
*T. variabile*	ERRO 2011012206	KP144106	-	Schneider et al. (2015)
*T. variabile*	ERRO 2009111802	KP144105	-	Schneider et al. (2015)
*T. variabile*	Personal collection: Carmel Sammut CS1378	OL653718	-	Sammut (2021)
*T. walteri*	ILLS 61047	JQ256430	-	Hustad et al. (2011)
*T. walteri*	PDD 74201	HQ222866	-	Wang et al. (2011)
*T. walteri*	PDD 75514	HQ222865	-	Wang et al. (2011)
*T. walteri*	PDD 75657	HQ222867	-	Wang et al. (2011)
*Xylaria hypoxylon*	CBS 122620	AM993141	KY610495	Peršoh et al. (2009)
*Xylona heveae*-**T**	CBS 132557	NR_121539	NG_066160	Gazis et al. (2012)

The newly generated sequences are marked in bold. Type specimens are indicated with **T**.

The datasets for each gene region were aligned using MAFFT v. 7 (Kuraku et al. 2013; Katoh et al. 2019) and trimmed in TrimAl v. 1.3 (Capella-Gutiérrez et al. 2009). The “gapthreshold” was set to 0.4 for ITS and 0.2 for LSU. Alignments were manually checked using BioEdit v. 7.0.9 (Hall et al. 2011). The ITS and LSU sequences were assembled into a combined dataset using SequenceMatrix v. 1.8 (Vaidya et al. 2011). The fasta format was converted to the phylip format for Maximum likelihood (ML) analysis and nexus format for Bayesian inference (BI) analysis using Alignment Transformation EnviRonment (ALTER) (Glez-Peña et al. 2010).

The ML and BI analyses were conducted in the CIPRES Science Gateway platform (Miller et al. 2010). The ML analysis was performed in RAxML-HPC2 on XSEDE v. 8.2.12 (Stamatakis 2014), with bootstrap support values (MLBS) calculated based on nonparametric bootstrapping with 1,000 replicates. The best-fit evolutionary model for each dataset was determined by MrModeltest v. 2.3, which selected the GTR+I+G for both ITS and LSU (Nylander et al. 2004). The BI analysis was performed using MrBayes on XSEDE v. 3.2.7a (Ronquist et al. 2011). Four simultaneous Markov chains were run for ten million generations, with trees sampled every 100^th^ generation (Cai et al. 2005). The first 25% of trees were discarded as burn-in, and the remaining trees were used to calculate Bayesian posterior probabilities (BPP) in the majority rule consensus tree. Convergence was confirmed by an average standard deviation of the split frequencies < 0.01 and an effective sample size (ESS) > 200 (Ronquist et al. 2011).

Phylogenetic trees were visualised and manipulated using FigTree v. 1.4.3 (http://tree.bio.ed.ac.uk/software/figtree/) and further edited in Adobe Illustrator CC 2018 (Adobe, USA) and Adobe Photoshop 2020 (Adobe, USA). Species boundaries among closely related taxa were determined using SplitsTree4 v. 4.19.2 (Huson and Bryant 2005), and recombination within the dataset was assessed using the PHI test (p). The *p* below 0.05 (*p* < 0.05) indicates significant recombination. The results of the PHI test were edited using Adobe Photoshop 2020 (Adobe, USA).

## 3. Results

### 3.1. Phylogenetic analyses

The guidelines of Chethana et al. (2021) were followed when introducing new species. The phylogenetic relationships of *Geoglossomycetes* were investigated based on the analyses of the combined LSU and ITS sequence data. The phylogenetic analyses were conducted with a dataset comprising 187 taxa, including 154 taxa from *Geoglossomycetes*, 29 taxa from other Ascomycota groups (*Archaeorhizomycetes*, *Candelariomycetes*, *Coniocybomycetes*, *Dothideomycetes*, *Laboulbeniomycetes*, *Lecanoromycetes*, *Leotiomycetes*, *Neolectomycetes*, *Novakomycetes*, *Orbiliomycetes*, *Pezizomycetes*, *Saccharomycetes*, *Schizosaccharomycetes*, *Sordariomycetes*, *Taphrinomycetes*, and *Xylonomycetes*), and four outgroup taxa (*Chaetospermum chaetosporum* CBS 154.59, *Laetisaria arvalis* CBS 131.82, *Marchandiomyces marsonii* ATCC MYA-4210, and *Mycotribulus mirabilis* CPC 14167). The alignment consists of 1,694 base pairs (bp) (ITS: 573 bp; LSU: 1,121 bp), with 32.33% gaps and undetermined characters. It has 1,260 distinct alignment patterns. The ML tree had a similar topology as the BI tree. The best ML tree is shown in Figure 1, with the final optimisation likelihood of −35,185.936016. In the BI analysis, the final average standard deviation of the split frequencies is 0.005564, indicating convergence.

**Figure 1.**
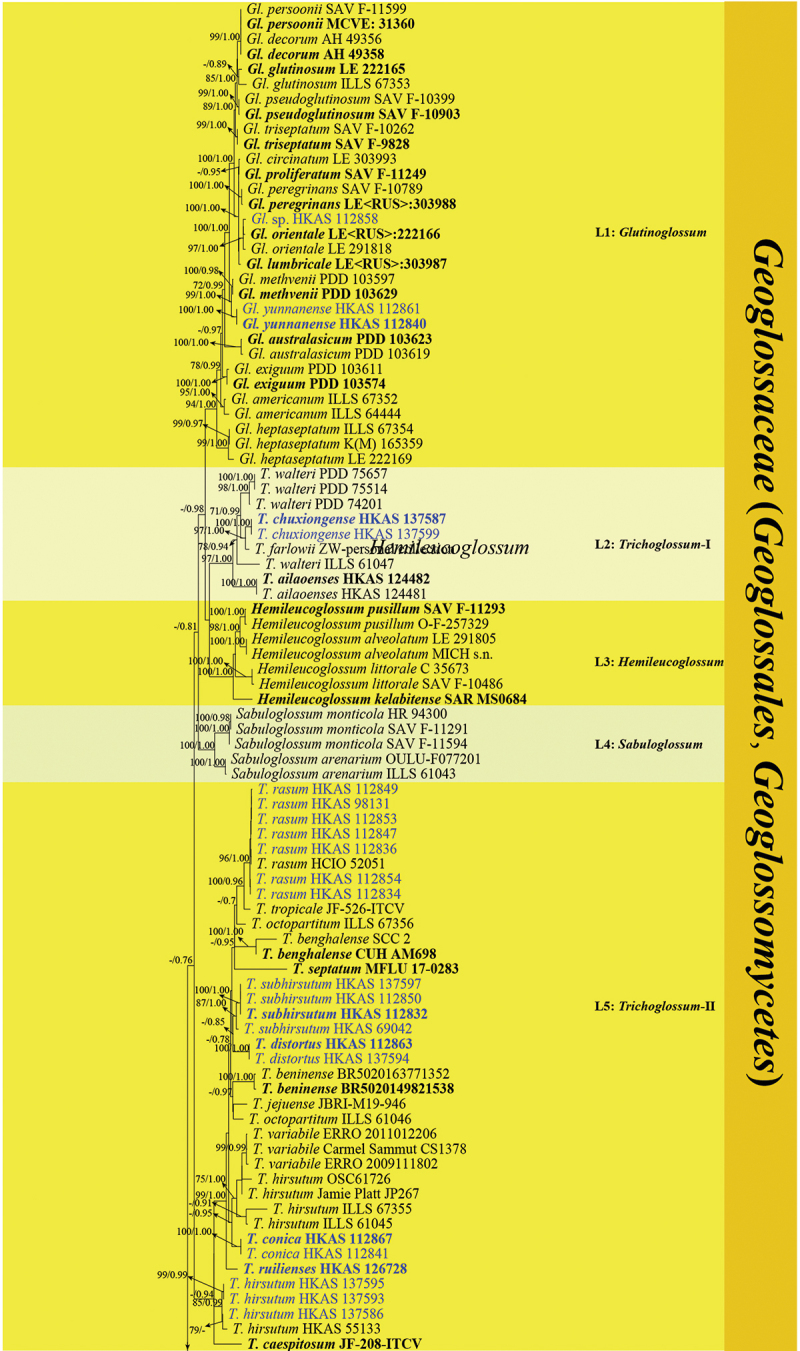
Phylogenetic tree derived from maximum likelihood analysis of the combined ITS and LSU rDNA sequences of *Geoglossomycetes*, using *Chaetospermum chaetosporum* (CBS 154.59), *Mycotribulus mirabilis* (CPC 14167), *Laetisaria arvalis* (CBS 131.82) and *Marchandiomyces marsonii* (ATCC MYA-4210) as outgroup taxa. MLBS ≥ 70% and BPP ≥ 0.70 are given near the nodes. The samples from this study are shown in blue. Type specimens are shown in bold.

In our phylogenetic tree, all *Geoglossaceae* taxa form a strongly supported clade with 91% bootstrap and 1.00 Bayesian posterior probability, consisting of ten lineages. These ten lineages are determined based on the phylogenetic analyses from this study and previous research (Kučera and Lizoň 2012; Hustad and Miller 2015a; Ekanayaka et al. 2017a; Fadnes et al. 2021). These ten clades represent nine genera; eight genera are monophyletic, while *Trichoglossum* is polyphyletic and dispersed across two lineages. Statistical support for the lineages varied, as shown in Figure 1.

**Figure 1.**
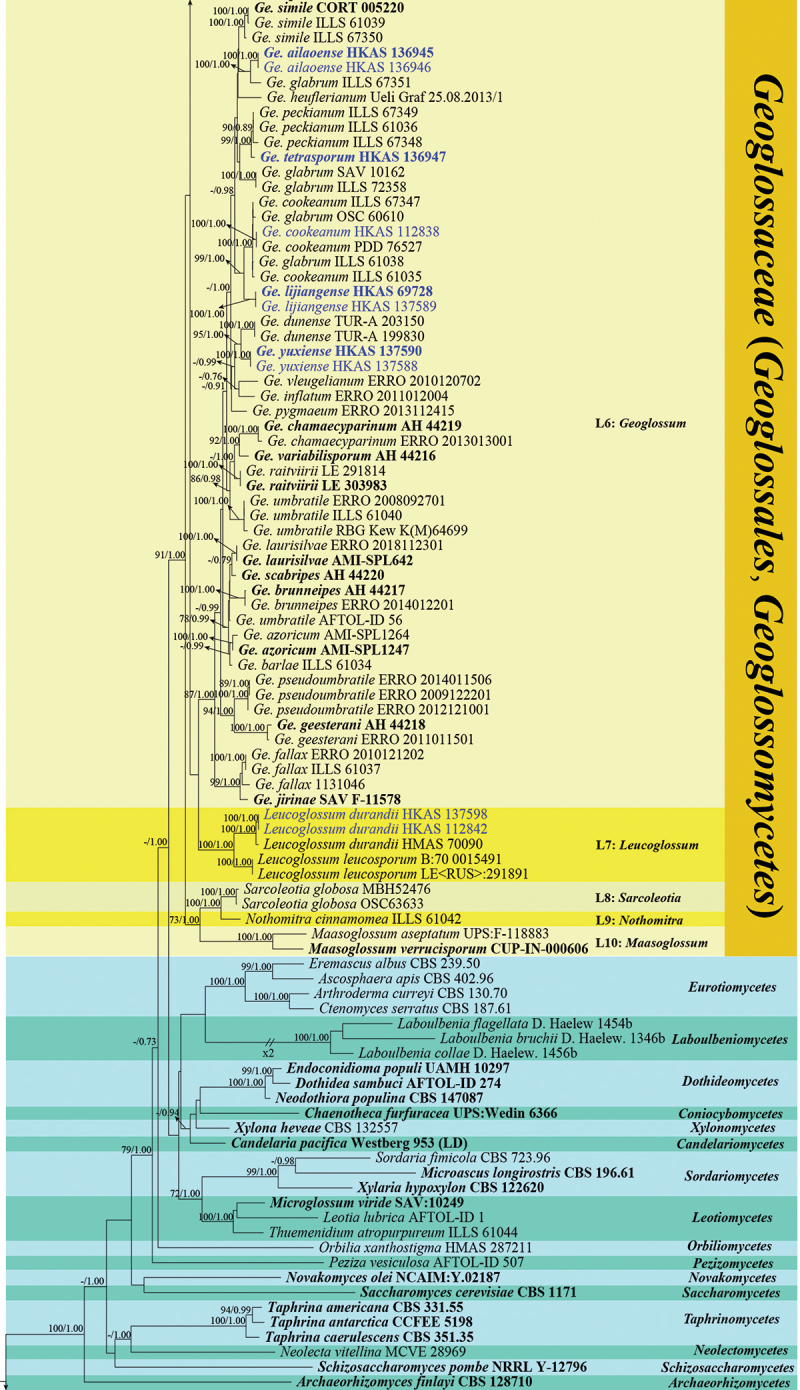
(Continued).

Our 34 samples nest within the clades of *Geoglossum*, *Glutinoglossum*, *Leucoglossum*, and *Trichoglossum* in our phylogenetic analyses. Within the clade of the *Geoglossum*, eight samples are present. *Geoglossum ailaoense* (HKAS 136945 and HKAS 136946) clusters with *Ge. glabrum* (ILLS 67351) with 100% bootstrap and 1.00 Bayesian posterior probability. *Geoglossum tetrasporum* (HKAS 136947) is sister to *Ge. peckianum* (ILLS 61036, ILLS 67348, and ILLS 67349) with 99% bootstrap and 1.00 Bayesian posterior probability. *Geoglossum cookeanum* (HKAS 112838) groups with *Ge. cookeanum* (PDD 76527) to form a strongly supported clade with 100% bootstrap and 1.00 Bayesian posterior probability. *Geoglossum lijiangense* (HKAS 69728 and HKAS 137589) forms a strongly supported clade separated from *Ge. cookeanum* (HKAS 112838, ILLS 61035, ILLS 67347, and PDD 76527) and *Ge. glabrum* (OSC 60610 and ILLS 61038) with 99% bootstrap and 1.00 Bayesian posterior probability. *Geoglossum yuxiense* (HKAS 137588 and HKAS 137590) clusters with *Ge. dunense* (TUR-A 199830 and TUR-A 203150) with 95% bootstrap and 1.00 Bayesian posterior probability.

One sample (HKAS 112858) clusters with *Glutinoglossum orientale* (LE<RUS>: 222166 and LE 291818) with 97% bootstrap and 1.00 Bayesian posterior probability. *Glutinoglossum yunnanense* (HKAS 112840 and HKAS 112861) clusters with *Gl. methvenii* (PDD 103597 and PDD 103629) with 99% bootstrap and 1.00 Bayesian posterior probability.

Two samples of *Leucoglossum durandii* (HKAS 112842 and HKAS 137598) cluster with *L. durandii* (HMAS 70090) with 100% bootstrap and 1.00 Bayesian posterior probability support within the *Leucoglossum* lineage.

The polyphyletic *Trichoglossum* taxa cluster into two lineages. *Trichoglossum chuxiongense* (HKAS 137587 and HKAS 137599) clusters with *T. farlowii* (ZW-personal collection) with 97% bootstrap and 1.00 Bayesian posterior probability within *Trichoglossum*-I clade. An additional 19 samples clustered within the *Trichoglossum*-II clade. Seven samples of *Trichoglossum rasum* (HKAS 98131, HKAS 112834, HKAS 112836, HKAS 112847, HKAS 112849, HKAS 112853, and HKAS 112854) cluster with *T. rasum* (HCIO 52051) with 96% bootstrap and 1.00 Bayesian posterior probability. Four samples of *T. subhirsutum* (HKAS 69042, HKAS 112832, HKAS 112850, and HKAS 137597) form a clade with 87% bootstrap and 1.00 Bayesian posterior probability, *T. distortus* samples (HKAS 112863 and HKAS 137594) form another strongly supported clade with 100% bootstrap and 1.00 Bayesian posterior probability; the two clades separate from each other by 29% bootstrap and 0.85 Bayesian posterior probability. *Trichoglossum conica* (HKAS 112841 and HKAS 112867) forms a clade separated from the clade comprising *T. variabile* (Carmel Sammut CS1378, ERRO 2009111802, and ERRO 2011012206), *T. hirsutum* (ILLS 61045, ILLS 67355, Jamie Platt JP267, and OSC 61726) with 36% bootstrap and 0.95 Bayesian posterior probability. *Trichoglossum ruiliense* (HKAS 126728) is nested within *Trichoglossum*-II with 69% bootstrap and 0.94 Bayesian posterior probability. Four samples of *T. hirsutum* (HKAS 55133, HKAS 137586, HKAS 137593, and HKAS 137595) form a clade with 85% bootstrap and 0.99 Bayesian posterior probability.

### 3.2. Taxonomy

In this study, we used 34 samples from China, proposed ten new species, and identified four known species, *Geoglossum cookeanum*, *Leucoglossum durandii*, *Trichoglossum hirsutum*, and *T. rasum*.

**Figure 2.**
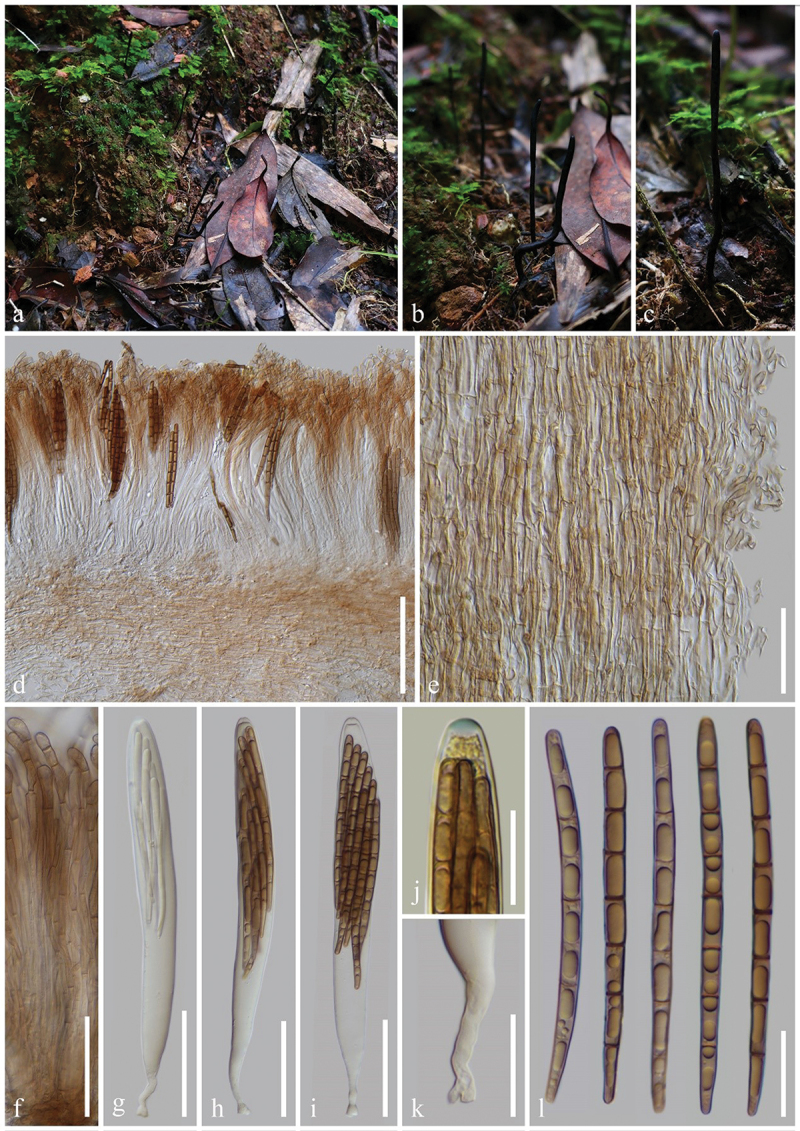
*Geoglossum ailaoense* (HKAS 136945, holotype). (a–c) Fresh ascomata. (d) Longitudinal section of an ascigerous portion. (e) Longitudinal section of a stipe. (f) Paraphyses. (g–i) Asci. (j) Ascus apex in MLZ. (k) Ascus base. (l) Ascospores. Scale bars: d = 100 µm, e–i = 50 µm, j–l = 20 µm.

***Geoglossum*** ***ailaoense*** H.L. Su, K.D. Hyde, Zhu L. Yang & Q. Zhao, sp. nov., Figure 2

*Index Fungorum number*: IF 903117; *Facesoffungi number*: FoF 16995.

*Etymology*: Referring to the type locality Ailao Mountains, Yunnan, China.

*Holotype*: HKAS 136945.

*Diagnosis*: *Geoglossum ailaoense* is similar to *Ge. glabrum*, but differs from the latter by its thinner ascigerous portion, shorter stipe, and paraphyses without constriction at the septa.

*Description*: *Saprobic* on soil among moss. Sexual morph: *Ascomata* gregarious, scattered, linear to cylindrical, stipitate, 2.5–4.5 cm in height when dried, smooth, without setae. *Ascigerous portion* cylindrical, sometimes compressed, straight, obtuse, 15–35 mm in height, 0.5–1 mm in diam. when dried, slightly thicker than stipe, dark brown to black, concolourous with the stipe, smooth. *Stipe* 10–20 mm in height, 0.5–1 mm in diam. when dried, cylindrical, slender, sometimes slightly compressed, straight to slightly curved. *Hymenium* 190–245 µm ($\overset{ˉ}{x}$ = 214 µm, *n* = 15) thick, consisting of paraphyses and asci at different stages of development. *Ascigerous portion interior* light brown to hyaline, composed of thin-walled hyphae of *textura porrecta*, 2.5–6.5 µm ($\overset{ˉ}{x}$ = 4.3 µm, *n* = 60) in diam. *Stipe surface* 15–70 µm ($\overset{ˉ}{x}$ = 29 µm, *n* = 20) thick, light brown to hyaline hyphae of *textura intricate*, septate, 1–6 µm ($\overset{ˉ}{x}$ = 2.8 µm, *n* = 99) in diam., the stipe surface covered with a thin layer of short, septate, irregularly arranged, brownish hyphae, 2.5–4.5 µm ($\overset{ˉ}{x}$ = 3.8 µm, *n* = 20) in diam. *Stipe interior* hyphae of *textura porrecta*, light brown, composed of thin-walled hyphae, 2.9–8.6 µm ($\overset{ˉ}{x}$ = 5.2 µm, *n* = 96) in diam. *Paraphyses* filiform to cylindrical, protruding above the asci, multi-septate, pale brown, lower portion 1.5–4.5 µm ($\overset{ˉ}{x}$ = 2.8 µm, *n* = 101) in diam., apical portion slightly fastigiate, slightly swollen, drop-shaped, or globose, 8–24.5 × 5–9 µm ($\overset{ˉ}{x}$ = 13.5 × 6.6 µm, *n* = 118), with slightly swollen internode. *Asci* (180–)185–210(−215) × 15–23(−25) µm ($\overset{ˉ}{x}$ = 198 × 19 µm, *n* = 31), 8-spored, cylindrical-clavate, attenuated towards the base, apically thick-walled, laterally thin-walled, with tapered, obtuse, amyloid apex, and croziers. *Ascospores* (139/4/1) (78–)81–98(−106) × (3.3–)3.9–5.5(−6.4) µm ($\overset{ˉ}{x}$ = 89 × 4.7 µm, Q = 13.9–26.4, ***Q*** = 19.18 ± 2.55), mostly fasciculate, clavate-filiform to acicular, obtuse ends, straight to slightly curved, thick-walled, multi-guttulate, mostly 7-septate, brown. Asexual morph: Not observed.

*Material examined*: China, Yunnan Province, Yuxi, Ailao Mountains, on soil among moss in evergreen broadleaf forests, 1 September 2021, H.L. Su, SHL159 (HKAS 136945, **holotype**); *ibid*., 2 September 2021, H.L. Su, SHL175 (HKAS 136946, **paratype**).

*Notes*: In the multi-loci phylogenetic analyses, *Geoglossum ailaoense* (HKAS 136945 and HKAS 136946) separates from *Ge. glabrum* (ILLS 67351) with 100% bootstrap and 1.00 Bayesian posterior probability. *Geoglossum ailaoense* is similar to *Ge. glabrum* in habitat, but they differ morphologically. *Geoglossum ailaoense* has linear to cylindrical ascomata with a cylindrical ascigerous portion nearly equal in diameter to its stipe (Figure 2a–c) (0.5–1 mm in diam.), whereas *Ge. glabrum* has clavate ascomata with a lanceolate ascigerous portion that is significantly thicker than its stipe (Ascigerous portion: 1–8 mm in diam.; Stipe: 0.4–4 mm in diam.) (Zhuang and Wang 1997; Kučera and Lizoň 2012; Hustad 2015). In addition, *Ge. glabrum* has paraphyses obviously constricted at the septa as shown in Hustad (2015), while *Ge. ailaoense* has paraphyses without that particular character. Furthermore, sequence comparisons between *Ge. ailaoense* (HKAS 136945) and *Ge. glabrum* (ILLS 67351) show a wide difference, they exhibit 41 base pairs (with 13 gaps) differences in the ITS region (498/539–7.8%), and 12 base pairs (with 1 gap) differences in the LSU region (968/980–1.2%) (Hustad et al. 2013; Jeewon and Hyde 2016). Based on our phylogenetic analyses, morphological studies, and sequence differences between *Ge. ailaoense* and *Ge. glabrum*, we introduce *Ge. ailaoense* as a new species.

***Geoglossum cookeanum*** Nannf. ex Minter & P.F. Cannon, IMI Descriptions of Fungi and Bacteria 204 (no. 2031): 1 (2015), Figure 3

**Figure 3.**
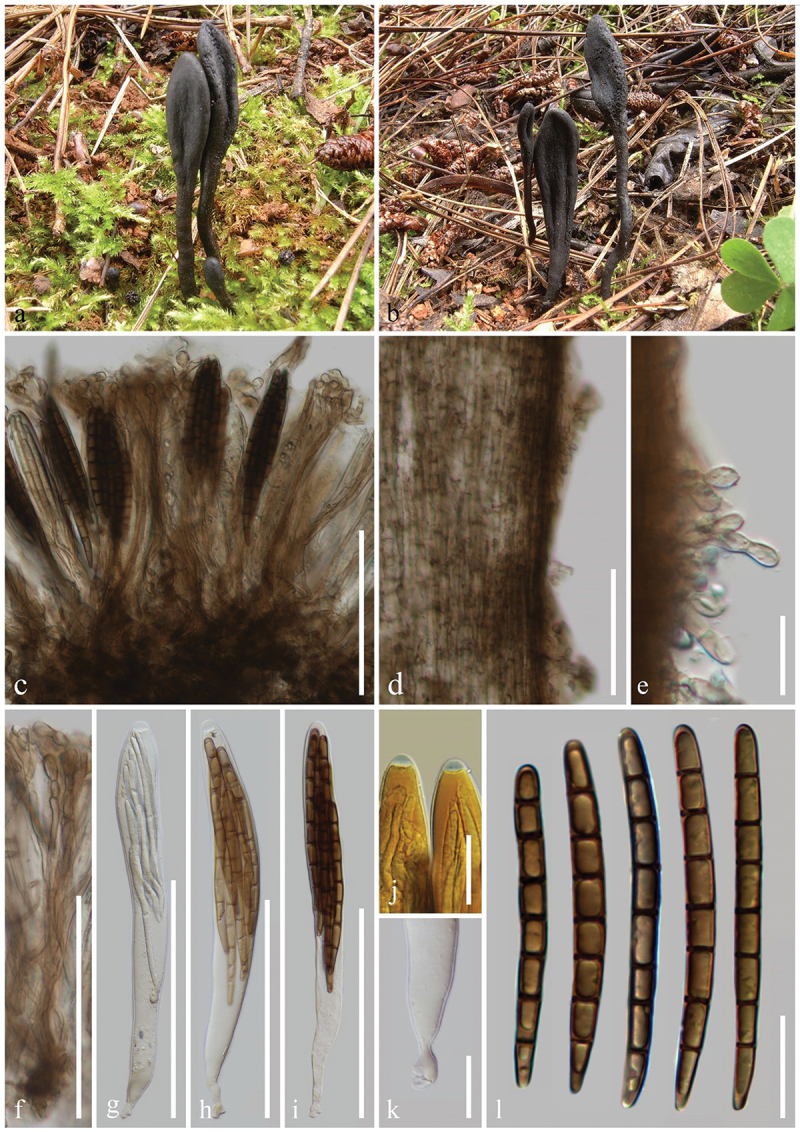
*Geoglossum cookeanum* (HKAS 112838). (a, b) Fresh ascomata. (c) Longitudinal section of an ascigerous portion. (d) Longitudinal section of a stipe. (e) Stipe surface. (f) Paraphyses. (g–i) Asci. (j) Ascus apex in MLZ. (k) Ascus base. (l) Ascospores. Scale bars: c, d, f–i = 100 µm, e, j–l = 20 µm.

*Index Fungorum number*: IF 551524; *Facesoffungi number*: FoF 17057.

*Description*: *Saprobic* on soil among moss. Sexual morph: *Ascomata* gregarious, scattered, clavate, stipitate, 1–3 cm in height, without setae. *Ascigerous portion* swollen, flattened lanceolate, with tapering, obtuse apex, 10–20 mm in height, 5–10 mm in diam., black, concolourous with the stipe. *Stipe* 15–25 mm in height, 2–5 mm in diam., cylindrical, straight to slightly curved. *Hymenium* 160–215 µm ($\overset{ˉ}{x}$ = 190 µm, *n* = 10) thick, consisting of paraphyses, and asci at different stages of development. *Ascigerous portion interior* brown to dark brown, composed of thick-walled hyphae of *textura intricata*, 2.8–5.8 µm ($\overset{ˉ}{x}$ = 3.9 µm, *n* = 41) in diam. *Stipe surface* 25–55 µm ($\overset{ˉ}{x}$ = 33 µm, *n* = 15) thick, brown hyphae of *textura porrecta*, septate, 3.5–8 µm ($\overset{ˉ}{x}$ = 5.5 µm, *n* = 50) in diam., in some areas the surface covered with a thin layer of short, frequently septate, irregularly arranged, light brown to hyaline hyphae, 3–6.5 µm ($\overset{ˉ}{x}$ = 4.7 µm, *n* = 30) in diam. *Stipe interior* hyphae of *textura porrecta*, brown, lighter than stipe surface, composed of thin-walled hyphae, 5.5–9.5 µm ($\overset{ˉ}{x}$ = 7.5 µm, *n* = 50) in diam. *Paraphyses* filiform, equal to asci, multi-septate, pale brown, lower portion 2.3–4.5 µm ($\overset{ˉ}{x}$ = 3.1 µm, *n* = 33) in diam., apical portion straight to slightly curved, swollen, 4.6–9.4 µm ($\overset{ˉ}{x}$ = 6.6 µm, *n* = 38) in diam., evenly and obviously constricted at the septa. *Asci* (155–) 160–195(−210) × (13–)16–21(−24) µm ($\overset{ˉ}{x}$ = 168 × 20 µm, *n* = 40), 8-spored, cylindrical-clavate, attenuated towards the base, apically thick-walled, laterally thin-walled, with tapered, obtuse, amyloid apex, and croziers. *Ascospores* (50/1/1) 65–75(−80) × (4.8–)5–5.8(−6.2) µm ($\overset{ˉ}{x}$ = 71 × 5.5 µm, Q = 11.2–14.7, ***Q*** = 12.9 ± 1.13), fasciculate, long-cylindrical, obtuse ends, straight to slightly curved, thick-walled, multi-guttulate, mostly 7-septate, brown. Asexual morph: Not observed.

*Material examined*: China, Yunnan Province, Kunming, the Kunming Institute of Botany, on soil among moss in coniferous forests, 25 September 2020, Z.L. Yang, Yang 6442 (HKAS 112838).

*Notes*: The species is recognised by clavate, stipitate, black ascomata, lanceolate, with a compressed ascigerous portion, cylindrical stipe, filiform paraphyses with barrel-shaped to globose, pale brown apical cells, relatively large (135–182 × 15–23 µm), 8-spored asci, and 7-septate, dark fuliginous ascospores measuring 55–92 × 5.5–7 µm (Kučera and Lizoň 2012; Hustad 2015). Our sample (HKAS 112838) groups with *Geoglossum cookeanum* (PDD 76527) forming a clade with 100% bootstrap and 1.00 Bayesian posterior probability in the combined tree. Additionally, our sample (HKAS 112838), *Ge. cookeanum* (PDD 76527, ILLS 67347, and ILLS 61035) and *Ge. glabrum* (OSC 60610 and ILLS 61038) form a clade with 100% bootstrap and 1.00 Bayesian posterior probability. *Geoglossum cookeanum* and *Ge. glabrum* differ in the characteristics of the stipe and the apical cells of the paraphyses (Nannfeldt 1942; Kučera and Lizoň 2012). Nannfeldt (1942) clearly distinguished *Ge. cookeanum* from *Ge. glabrum* based on differences in the stipe and paraphyses. *Geoglossum cookeanum* has a short and broad stipe (9–41 × 2–6 mm vs. 6–86 × 0.4–3 mm) bearing some small warts or hyphal tufts on its surface, whereas *Ge. glabrum* has long and slender stipe covered by a weft of long, dark-brown mycelium-like hyphae (Nannfeldt 1942; Zhuang and Wang 1997; Kučera and Lizoň 2012). Additionally, *Ge. glabrum* has paraphyses with larger (8–15 µm thick vs. 6–10 µm thick), very dark (almost opaque) tips, while *Ge. cookeanum* has paraphyses with smaller, pale brown tips (Nannfeldt 1942; Kučera and Lizoň 2012). Based on these morphological and phylogenetic analyses, we identify our sample (HKAS 112838) as *Ge. cookeanum*.

**Figure 4.**
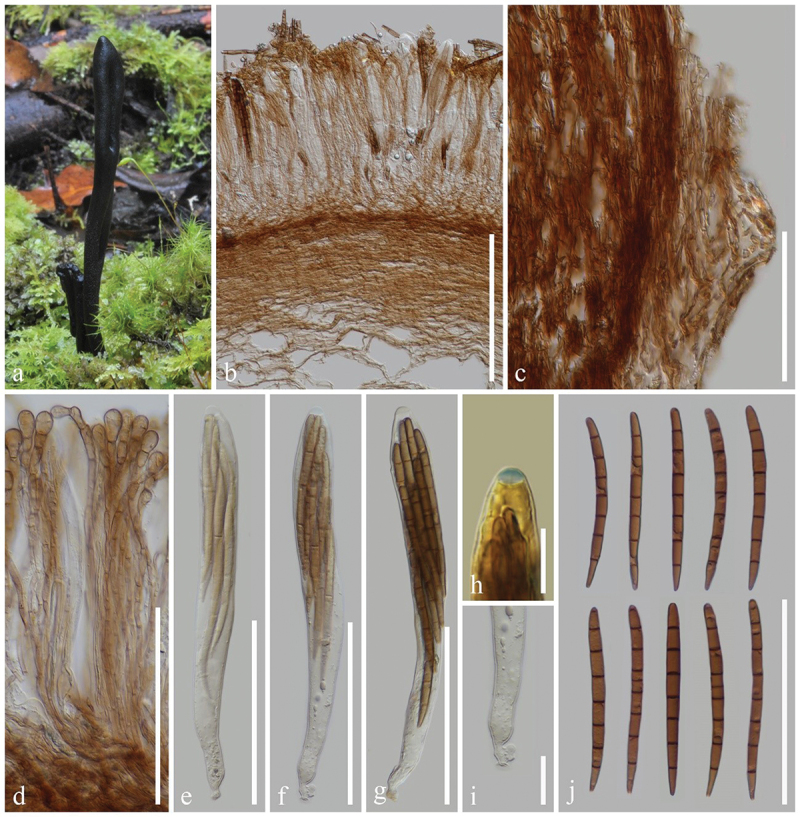
*Geoglossum lijiangense* (HKAS 69728, holotype). (a) Fresh ascomata. (b) Longitudinal section of an ascigerous portion. (c) Longitudinal section of a stipe with squamules. (d) Paraphyses. (e–g) Asci. (h) Ascus apex in MLZ. (i) Ascus base. (j) Ascospores. Scale bars: b = 200 µm, c–g, j = 100 µm, h, i = 20 µm.

***Geoglossum*** ***lijiangense*** H.L. Su, K.D. Hyde, Zhu L. Yang & Q. Zhao, sp. nov., Figure 4

*Index Fungorum number*: IF 903118; *Facesoffungi number*: FoF 16996.

*Etymology*: Referring to the type locality Lijiang, Yunnan, China.

*Holotype*: HKAS 69728.

*Diagnosis*: *Geoglossum lijiangense* is somewhat similar to *Ge. cookeanum* and *Ge. glabrum* in habitat and macro-morphological characteristics. However, *Ge. lijiangense* has a thicker ascigerous portion, longer asci, and longer ascospores than *Ge. cookeanum* and *Ge. glabrum*.

*Description*: *Saprobic* on decayed wood. Sexual morph: *Ascomata* solitary to gregarious, clavate, tongue-shaped, stipitate, 5–8 cm in height, smooth, without setae. *Ascigerous portion* terete to lanceolate, more or less compressed, with obviously shallow and longitudinal depressions in the centre, and tapered apex, 30–45 mm in height, 3–6 mm in diam., similar thickness to the stipe, dark brown to black, concolourous with the stipe, smooth. *Stipe* 35–60 mm in height, 2–4 mm in diam., cylindrical or slightly compressed, straight, brownish-black to black, rough, with tiny squamules. *Hymenium* 205–245 µm ($\overset{ˉ}{x}$ = 222 µm, *n* = 19) thick, consisting of paraphyses and asci at different stages of development. *Ascigerous portion interior* brown, composed of thin-walled hyphae of *textura porrecta*, 0.9–2.1 µm ($\overset{ˉ}{x}$ = 1.5 µm, *n* = 57) in diam. *Stipe surface* 23–68 µm ($\overset{ˉ}{x}$ = 44 µm, *n* = 13) thick, brown hyphae of *textura porrecta*, frequently septate, 2.5–6.5 µm ($\overset{ˉ}{x}$ = 4 µm, *n* = 103) in diam., in some areas hyphae aggregate into a protrusion. *Stipe interior* hyphae of *textura porrecta*, brown, composed of thin-walled hyphae, 3.2–6.7 µm ($\overset{ˉ}{x}$ = 4.7 µm, *n* = 217) in diam. *Paraphyses* filiform, protruding above the asci, multi-septate, pale brown, lower portion 2.4–4.5 µm ($\overset{ˉ}{x}$ = 3.4 µm, *n* = 122) in diam., apical portion, straight, swollen, globose, 9.5–17 × 6–9.5 µm ($\overset{ˉ}{x}$ = 12.8 × 8 µm, *n* = 97), slightly constricted at the septa. *Asci* 210–245(−250) × (15–)18–28(−30) µm ($\overset{ˉ}{x}$ = 225 × 22 µm, *n* = 41), 8-spored, cylindrical-clavate, attenuated towards the base, apically thick-walled, laterally thin-walled, with tapered, obtuse, amyloid apex, and croziers. *Ascospores* (185/4/1) (73–)81–103(−116) × (4.5–)5–7.5(−8.5) µm ($\overset{ˉ}{x}$ = 91.2 × 5.4 µm, Q = 10–26, ***Q*** = 15.69 ± 2.55), mostly fasciculate, clavate-filiform to acicular, round apex and relatively acuminate base, straight to slightly curved, thin-walled, multi-guttulate, 7-septate, light brown when immature, dark brown when mature. Asexual morph: Not observed.

*Material examined*: China, Yunnan Province, Lijiang, Jiuxianghe, on decayed wood in mixed conifer-broadleaf forests, 20 August 2010, Q. Zhao, ZQ1007 (HKAS 69728, **holotype**); China, Yunnan Province, Lijiang, Yulong, on soil among moss in broad-leaf forests, 13 September 2022, H.L. Su, SHL243 (HKAS 137589, **paratype**).

*Notes*: In our phylogenetic analyses, *Geoglossum lijiangense* (HKAS 69728 and HKAS 137589) forms a distinct clade from *Ge. cookeanum* and *Ge. glabrum* with 99% bootstrap and 1.00 Bayesian posterior probability. Due to the absence of LSU sequences in two samples of *Ge. lijiangense* (HKAS 69728 and HKAS 137589), we performed both single-gene and multi-gene phylogenetic analyses using the ITS and LSU of this species. Our ITS phylogeny (Additional file 1) and ITS-LSU phylogeny (Figure 1) revealed the same phylogenetic relationships between *Ge. lijiangense*, *Ge. Cookeanum*, and *Ge. glabrum*. *Geoglossum lijiangense* has a habitat similar to *Ge. cookeanum* and *Ge. glabrum*. *Geoglossum lijiangense* lives on decayed wood or soil among moss, *Ge. cookeanum* lives with moss or grass, or in grassy sand dunes and dune slacks, and *Ge. glabrum* grows on soil, often associated with *Sphagnum*. *Geoglossum lijiangense* shares morphological similarities with *Ge. cookeanum* and *Ge. glabrum*, including black to dark-brown ascomata and 7-septate, dark fuliginous ascospores. However, *Ge. lijiangense* differs from *Ge. cookeanum* in having a thicker ascigerous portion (30–45 mm vs. 8–15 mm), and longer asci (209–249 × 15–30 µm vs. 163–185 × 14–22 µm). Compared to *Ge. glabrum*, *Ge. lijiangense* has a thicker ascigerous portion (30–45 mm vs. 5–8 mm), and longer asci (209–249 × 15–30 µm vs. 150–210 × 15–24 µm) (Kučera and Lizoň 2012; Hustad 2015). Therefore, *Ge. lijiangense* is introduced as a new species based on these morphological and phylogenetic differences.

**Figure 1.**
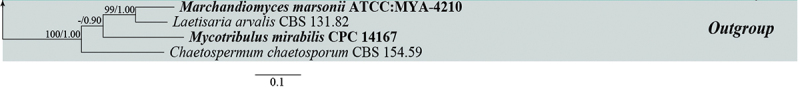
(Continued).

**Figure 5.**
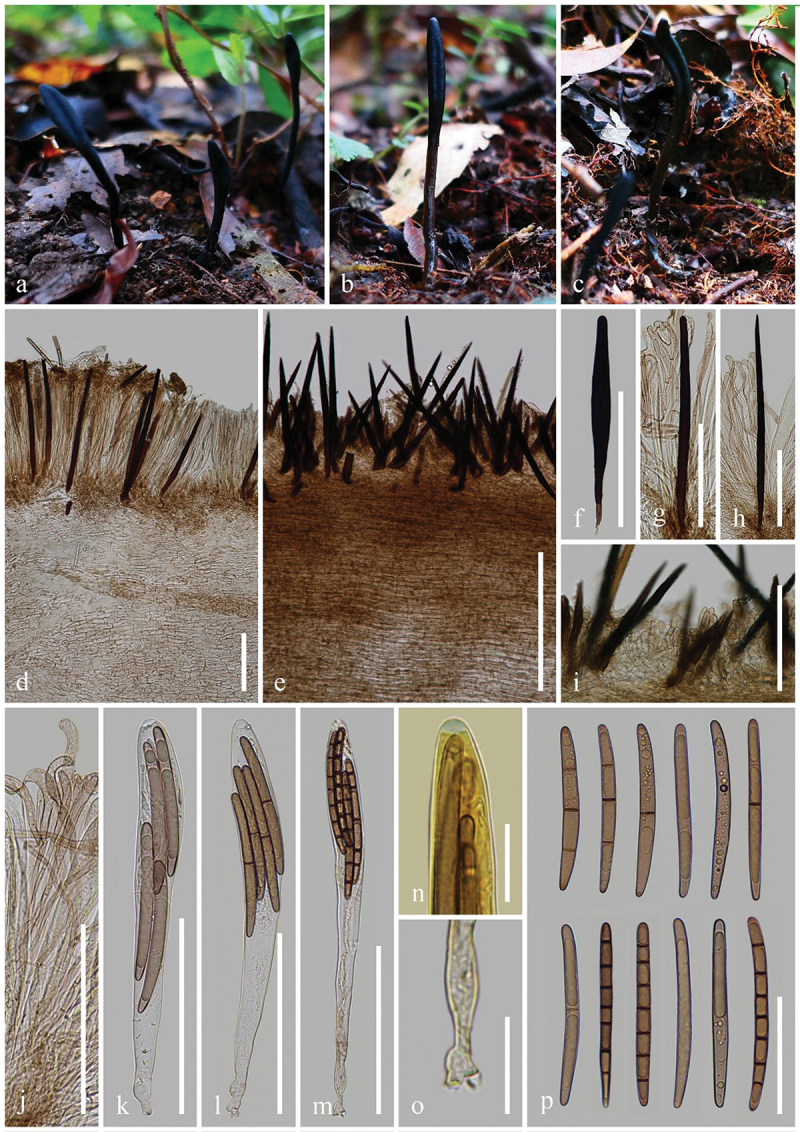
*Geoglossum tetrasporum* (HKAS 136947, holotype). (a–c) Fresh ascomata. (d) Longitudinal section of an ascigerous portion. (e) Longitudinal section of a stipe with setae. (f–h) Setae of hymenium. (i) Stipe surface. (j) Paraphyses. (k–m) Asci. (n) Ascus apex in MLZ. (o) Ascus base. (p) Ascospores. Scale bars: d–m = 100 µm, n, o = 20 µm, p = 50 µm.

***Geoglossum*** ***tetrasporum*** H.L. Su, K.D. Hyde, Zhu L. Yang & Q. Zhao, sp. nov., Figure 5

*Index Fungorum number*: IF 903119; *Facesoffungi number*: FoF 16997.

*Etymology*: Referring to 4-spored asci.

*Holotype*: HKAS 136947.

*Diagnosis*: Differs from all other *Geoglossum* species by all asci with four ascospores.

*Description*: *Saprobic* on soil among moss. Sexual morph: *Ascomata* scattered, gregarious, clavate, stipitate, 5–7.5 cm in height, hirsute, with numerous setae. *Ascigerous portion* swollen, cylindrical, compressed, mostly with obviously shallow and longitudinal depressions in the centre, and tapered, obtuse apex, 10–25 mm in height, 1.5–3 mm in diam., dark, relatively drier than stipe, densely hirsute from setae. *Stipe* 40–65 mm in height, 1–1.5 mm in diam., cylindrical, straight to slightly curved, brown, concolorous with ascigerous portion, or lighter than the ascigerous portion, densely hirsute from setae when fresh. *Hymenium* 205–265 µm ($\overset{ˉ}{x}$ = 235 µm, *n* = 33) thick (without setae), consisting of setae, paraphyses, and asci at different stages of development. *Ascigerous portion interior* light brown to hyaline, composed of thin-walled cells of *textura prismatica*, 11.5–44.5 × 3.5–10.5 µm ($\overset{ˉ}{x}$ = 26.5 × 7 µm, *n* = 81). *Stipe surface* 65–165 µm ($\overset{ˉ}{x}$ = 122 µm, *n* = 35) thick (without setae), composed of light brown to hyaline hyphae, frequently septate, 5.2–12.4 µm ($\overset{ˉ}{x}$ = 7.8 µm, *n* = 28) in diam., arranged parallel to setae. *Stipe interior* hyphae of *textura porrecta*, brown, darker than stipe surface, composed of thin-walled hyphae, 4.5–9.5 µm ($\overset{ˉ}{x}$ = 6.9 µm, *n* = 272) in diam. *Setae* 120–250 × (8–)8.5–15.5(−17) µm ($\overset{ˉ}{x}$ = 186 × 11 µm, *n* = 31), heavily protruding above paraphyses and asci, acicular, most with sharp apex, sometimes with obtuse apex, aseptate, dark brown to black, thick-walled. *Paraphyses* filiform, protruding above the asci, multi-septate, pale brown, lower portion 1.8–5 µm ($\overset{ˉ}{x}$ = 3 µm, *n* = 172) in diam., apical portion curved, swollen, 4.3–9.5 µm ($\overset{ˉ}{x}$ = 6.3 µm, *n* = 52) in diam. *Asci* 205–235(−240) × (13–)16–21(−24) µm ($\overset{ˉ}{x}$ = 216 × 19 µm, *n* = 46), 4-spored, cylindrical-clavate, attenuated towards the base, apically thick-walled, laterally thin-walled, with tapered, obtuse, amyloid apex, and croziers. *Ascospores* (122/4/1) (65–)72–85(−90) × (4.5–)5–7 (−7.5) µm ($\overset{ˉ}{x}$ = 79 × 5.9 µm, Q = 10.3–18, ***Q*** = 13.5 ± 1.34), fasciculate, cylindrical, obtuse ends, straight to slightly curved, thick-walled, multi-guttulate, mostly 2-guttulate, 1–7-septate, brown. Asexual morph: Not observed.

*Material examined*: China, Yunnan Province, Yuxi, Ailao Mountains, on soil among moss in evergreen broadleaf forests, 2 September 2021, H.L. Su, SHL 186 (HKAS 136947, **holotype**).

*Notes*: Phylogenetic analyses based on combined ITS and LSU sequences demonstrate that *Geoglossum tetrasporum* (HKAS 136947) is sister to *Ge. peckianum* (ILLS 61036, ILLS 67349, and ILLS 67348) with 99% bootstrap and 1.00 Bayesian posterior probability. *Geoglossum tetrasporum* is similar to *Ge. peckianum* in having clavate ascomata with compressed ascigerous portion. However, *Ge. tetrasporum* and *Ge. peckianum* differ in having thinner ascigerous portion (1.5–3 mm vs. 5–20 mm), slenderer stipe (1–1.5 mm vs. 6 mm), and shorter asci (205–240 × 13–24 µm vs. 240–275 × 18–25 µm). Additionally, *Ge. tetrasporum* has slender paraphyses with swollen and curved apex, 4-spored asci, and 1–7-septate ascospores, while *Ge. peckianum* has slender paraphyses with coiled apex, 8-spored asci, and mostly 15-septate ascospores. In addition, *Ge. tetrasporum* and *Ge. peckianum* have different habitats. *Geoglossum tetrasporum* grows on soil among moss in evergreen broadleaf forests. However, *Ge. peckianum* grows on forest soil and on rotting logs in mixed deciduous forests (Hustad 2015). Based on the nucleotide sequence comparisons, *Ge. tetrasporum* (HKAS 136947) differs from *Ge. peckianum* (ILLS 61036, ILLS 67348, and ILLS 67349) as shown in Table 2. Due to these differences in the morphology, ecology, phylogeny, and sequences, we introduce *Ge. tetrasporum* as a new species.

**Table 2.** Comparison of nucleotide differences among *Geoglossum tetrasporum* (HKAS 136947), *Ge. peckianum* (ILLS 61036, ILLS 67348, and ILLS 67349) sequences.

Taxa for comparison	ITS	LSU
*Ge. tetrasporum* (HKAS 136947) vs. *Ge. peckianum* (ILLS 61036)	12/532–2.3% (5 gaps)	-
*Ge. tetrasporum* (HKAS 136947) vs. *Ge. peckianum* (ILLS 67348)	21/531–4.0% (8 gaps)	7/862–0.8%
*Ge. tetrasporum* (HKAS 136947) vs. *Ge. peckianum* (ILLS 67349)	12/532–2.3% (5 gaps)	6/966–0.6%

**Figure 6.**
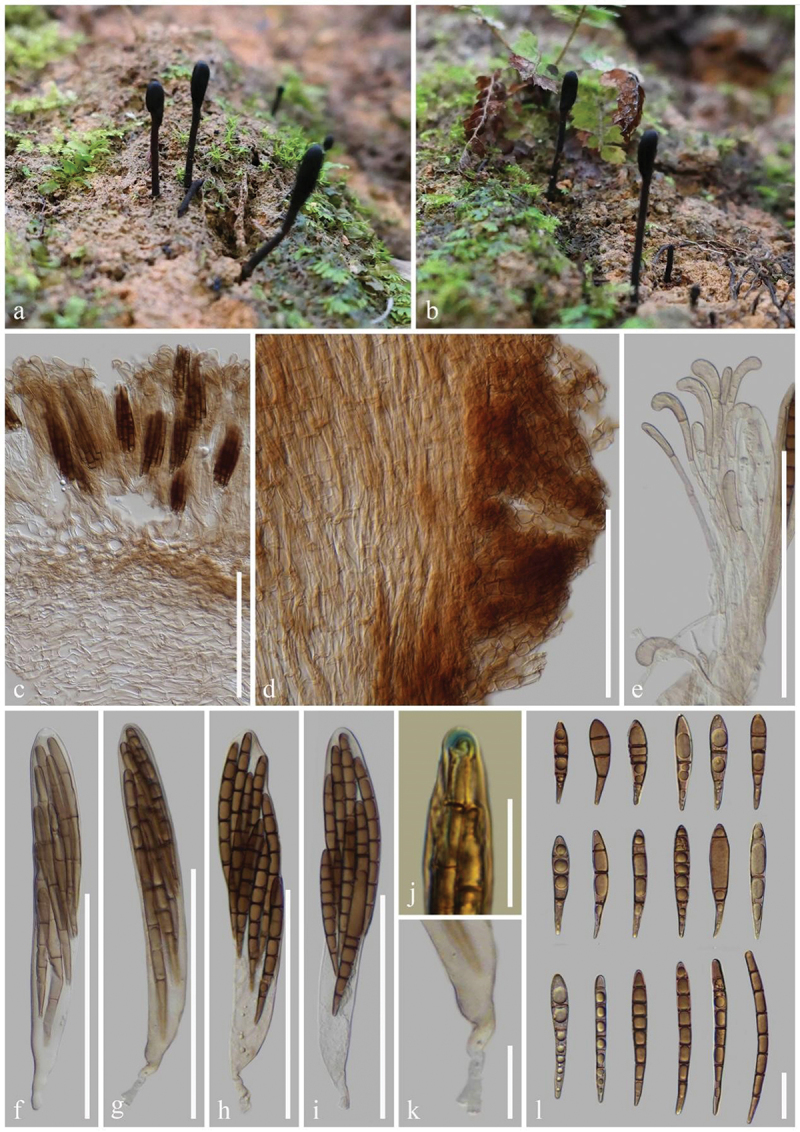
*Geoglossum yuxiense* (HKAS 137588, holotype). (a, b) Fresh ascomata. (c) Longitudinal section of an ascigerous portion. (d) Longitudinal section of stipe surface. (e) Paraphyses. (f–i) Asci. (j) Ascus apex in MLZ. (k) Ascus base. (l) Ascospores. Scale bars: c–i = 100 µm, j–l = 20 µm.

***Geoglossum*** ***yuxiense*** H.L. Su, K.D. Hyde, Zhu L. Yang & Q. Zhao, sp. nov., Figure 6

*Index Fungorum number*: IF 903120; *Facesoffungi number*: FoF 16998.

*Etymology*: Referring to the type locality Yuxi, Yunnan, China.

*Holotype*: HKAS 137588.

*Diagnosis*: *Geoglossum yuxiense* is somewhat similar to *Ge. dunense* in clavate ascospores. However, the 2 species have markedly different macromorphologies, paraphyses, and habitats.

*Description*: *Saprobic* on soil among moss. Sexual morph: *Ascomata* scattered to gregarious, clavate, stipitate, 1.2–2.5 cm in height when dried, smooth, without setae. *Ascigerous portion* swollen, cylindrical to clavate, sometimes slightly compressed, with obtuse apex, 3–7 mm in height, 1–2 mm in diam. when dried, thicker than stipe, dark, concolourous with the stipe, smooth, without setae. *Stipe* 9–22 mm in height, 0.2–0.7 mm in diam. when dried, cylindrical, slightly compressed, straight, rough, with tiny squamules. *Hymenium* 145–200 µm ($\overset{ˉ}{x}$ = 170 µm, *n* = 22) thick, consisting of paraphyses and asci at different stages of development. *Ascigerous portion interior* hyaline to light brown, composed of thin-walled cells of *textura angularis*, 10.2–22.7 × 6–13 ($\overset{ˉ}{x}$ = 16.4 × 9 µm, *n* = 73) in diam. *Stipe surface* 50–160 µm ($\overset{ˉ}{x}$ = 98 µm, *n* = 13) thick, brown hyphae of *textura porrecta*, frequently septate, 2.4–6.6 µm ($\overset{ˉ}{x}$ = 4.2 µm, *n* = 36) in diam., in some areas hyphae aggregate into a protrusion. *Stipe interior* hyphae of *textura porrecta*, brown, composed of thin-walled hyphae, 3.7–10.3 µm ($\overset{ˉ}{x}$ = 7.1 µm, *n* = 95) in diam. *Paraphyses* filiform to clavate, protruding above the asci, multi-septate, hyaline to pale brown, lower portion 1.5–3.2 µm ($\overset{ˉ}{x}$ = 2.2 µm, *n* = 53) in diam., apical portion curved, swollen, 4.1–8.6 µm ($\overset{ˉ}{x}$ = 6.3 µm, *n* = 58) in diam. *Asci* 155–180(−185) × (13–)15–23(−27) µm ($\overset{ˉ}{x}$ = 165 × 19 µm, *n* = 63), 8-spored, cylindrical-clavate, attenuated towards the base, apically thick-walled, laterally thin-walled, with tapered, obtuse, amyloid apex, and croziers. *Ascospores* (257/4/1) (31–)44–87(−97) × (3.8–)4.6–7.7(−10) µm ($\overset{ˉ}{x}$ = 66.5 × 5.8 µm, Q = 3.3–23.1, ***Q*** = 11.78 ± 3.26), partially fasciculate, morphologically various, mostly clavate, some filiform, falciform, acicular, some like tadpoles, with tapering, round apex, swollen middle and relatively acuminate base, straight to curved, thin-walled, multi-guttulate, 3–7-septate, mostly dark brown, sometimes light brown. Asexual morph: Not observed.

*Material examined*: China, Yunnan Province, Yuxi, Ailao Mountains, on soil among moss in evergreen broadleaf forests, 3 September 2021, H.L. Su, SHL 197 (HKAS 137588, **holotype**); *ibid*., 4 September 2021, H.L. Su, SHL 208 (HKAS 137590, **paratype**).

*Notes*: In our phylogenetic analyses, *Geoglossum yuxiense* (HKAS 137588 and HKAS 137590) clusters with *Ge. dunense* (TUR-A 199830 and TUR-A 203150) with 95% bootstrap and 1.00 Bayesian posterior probability. *Geoglossum yuxiense* shares several characteristics with *Ge. dunense*, including clavate, 8-spored asci with an amyloid apical pore, and fusiform to subfusiform, rarely subcylindrical ascospores. *Geoglossum yuxiense* and *Ge. dunense* have distinctly different macromorphology. In contrast to clavate ascomata with cylindrical to clavate ascigerous portion of *Geoglossum yuxiense*, *Ge. dunense* has club-shaped ascomata with an enlarged, occasionally cerebriform or rarely tongue-shaped, irregularly lobed ascigerous portion, which is clearly demonstrated by the pictures of this study and Loizides et al. (2015). Moreover, paraphyses in *Ge. yuxiense* are filiform with a curvy and swollen apical portion, whereas those of *Ge. dunense* are irregularly polymorphic, mostly moniliform, flexuous, or contorted, sometimes branching, with irregularly clavate thickenings, often constricted at the septa, and enlarged, clavate, subcapitate, occasionally forked or hooked tips. Additionally, *Ge. yuxiense* produces 3–7-septate ascospores, whereas *Ge. dunense* typically bears 0–4-septate ascospores. Furthermore, *Ge. yuxiense* and *Ge. dunense* have markedly different habitats. *Geoglossum yuxiense* was collected on soil among moss in evergreen broad-leaf forests in September. However, *Ge. dunense* was found in moist coastal dunes under *Juniperus phoenicea*, *Fumana thymifolia*, and *Olea europaea* from January to February. In addition, a sequence comparison between *Ge. yuxiense* (HKAS 137590) and *Ge. dunense* (TUR-A 199830) revealed 36 base pair differences (with 5 gaps) in the ITS region (36/474–7.6%), and 17 base pair differences (with 4 gaps) in the LSU region (17/812–2.1%) (Loizides et al. 2015; Jeewon and Hyde 2016). As a result, *Ge. yuxiense* is proposed as a new species.

**Figure 7.**
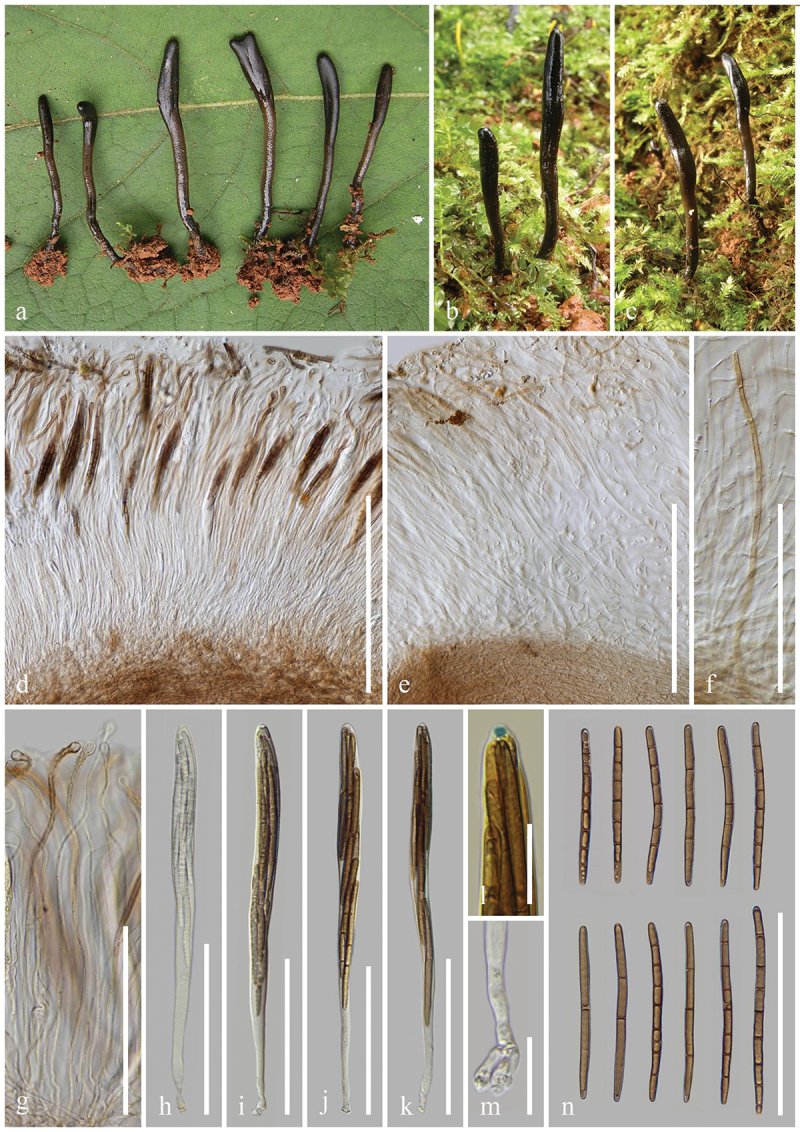
*Glutinoglossum yunnanense* (HKAS 112840, holotype). (a–c) Fresh ascomata. (d) Longitudinal section of an ascigerous portion. (e) Longitudinal section of stipe surface. (f) Setae of the stipe. (g) Paraphyses. (h–k) Asci. (l) Ascus apex in MLZ. (m) Ascus base. (n) Ascospores. Scale bars: d, e = 200 µm, f–k, n = 100 µm, l, m = 20 µm.

***Glutinoglossum yunnanense*** H.L. Su, K.D. Hyde, Zhu L. Yang & Q. Zhao, sp. nov., Figure 7

*Index Fungorum number*: IF 903121; *Facesoffungi number*: FoF 16999.

*Etymology*: Referring to the type locality Yunnan, China.

*Holotype*: HKAS 112840.

*Diagnosis*: *Glutinoglossum yunnanense* is similar to *Gl. methvenii*, but is distinguished from the latter by setae, paraphyses, and larger ascospores.

*Description*: *Saprobic* on soil among moss. Sexual morph: *Ascomata* scattered to gregarious, tongue-shaped to lanceolate, stipitate, 2–4 cm in height, smooth, without setae. *Ascigerous portion* slightly swollen, lanceolate to clavate, or cylindrical, more or less compressed, straight to slightly twisted, with tapering or widening, obtuse apex, 6–15 mm in height, 2–3 mm in diam., black, viscid. *Stipe* 10–20 mm in height, 1–3 mm in diam., cylindrical, sometimes slightly compressed, straight to slightly curved, dark brown, viscid, becoming gelatinous when wet. *Hymenium* 230–370 µm ($\overset{ˉ}{x}$ = 303 µm, *n* = 32) thick, consisting of paraphyses and asci at different stages of development. *Ascigerous portion interior* dark brown, composed of thin-walled hyphae of *textura porrecta*, 1.5–3.8 µm ($\overset{ˉ}{x}$ = 2.3 µm, *n* = 336) in diam. *Stipe surface* 225–435 µm ($\overset{ˉ}{x}$ = 312 µm, *n* = 13) thick, composed of light brown setae and hyaline hyphae-like paraphyses. *Stipe interior* hyphae of *textura porrecta*, brown, composed of thin-walled hyphae, 1.7–4 µm ($\overset{ˉ}{x}$ = 2.7 µm, *n* = 47) in diam. *Paraphyses-like hyphae of stipe surface* 1.9–3.8 µm ($\overset{ˉ}{x}$ = 2.8 µm, *n* = 118) in diam., filiform, obtuse apex, septate, rough, hyaline. *Setae* 2.4–3.7 µm ($\overset{ˉ}{x}$ = 3.1 µm, *n* = 21) in diam., filiform, straight to slightly curved, obtuse apex, septate, light brown, darker than hyphae of stipe surface, rough. *Paraphyses* filiform, protruding above the asci, septate, lower portion 1.3–3 µm ($\overset{ˉ}{x}$ = 2 µm, *n* = 124) in diam., hyaline, apical portion straight to naturally curved, swollen, globose, 3.1–8.1 µm ($\overset{ˉ}{x}$ = 5.3 µm, *n* = 57), mostly light brown. *Asci* 225–265 × (11–)12–16(−17) µm ($\overset{ˉ}{x}$ = 245 × 13.9 µm, *n* = 63), 8-spored, thin cylindrical-clavate, attenuated towards the base, apically thick-walled, laterally thin-walled, with tapered, obtuse, amyloid apex, and croziers. *Ascospores* (174/4/1) (72–)76–95(−103) × (3.4–)4–5.3(−5.9) µm ($\overset{ˉ}{x}$ = 85 × 4.7 µm, Q = 13.5–27, ***Q*** = 18.2  ± 1.97), partly fasciculate, clavate-filiform, obtuse ends, straight to slightly curved, thin-walled, multi-guttulate, 3–7-septate, brown. Asexual morph: Not observed.

*Material examined*: China, Yunnan Province, Kunming, the Kunming Institute of Botany, on soil among moss in broad-leaf forests, 26 September 2020, Z.L. Yang, Yang 6444 (HKAS 112840, **holotype**); *ibid*., 2 October 2020, Z.L. Yang, Yang 6465 (HKAS 112861, **paratype**).

*Notes*: In our phylogenetic analyses, *Glutinoglossum yunnanense* (HKAS 112840 and HKAS 112861) clusters as sister to *Gl. methvenii* with 99% bootstrap and 1.00 Bayesian posterior probability. *Glutinoglossum yunnanense* is similar to *Gl. methvenii* in black, glutinous ascomata, clavate ascigerous portion, terete stipe, clavate asci, and 3–7-septate, mature brown ascospores. However, *Gl. yunnanense* differs from *Gl. methvenii* by having ascomata with setae and larger ascospores (72–103 × 3.4–5.9 µm vs. 65–79 × 4–6.5 µm), whereas *Gl. methvenii* lacks setae. Furthermore, the paraphyses of *Gl. methvenii* have a curved to slightly hooked apex covered with brown gelatinous matter, while the paraphyses of *Gl. yunnanense* have a straight or slightly curved apex without gelatinous matter (Hustad and Miller 2015b). *Glutinoglossum yunnanense* and *Gl. methvenii* share a similar habitat and live on moss. However, their distributions are different, *Gl. yunnanense* was found in China, while *Gl. methvenii* was collected in Australia and New Zealand (Hustad and Miller 2015b). *Glutinoglossum yunnanense* (HKAS 112840) and *Gl. methvenii* (PDD 103629) differ by 15 base pairs (with 3 gaps) in the ITS region (15/544–2.8%), and 4 base pairs in the LSU region (548/552–0.7%) (Jeewon and Hyde 2016). *Glutinoglossum yunnanense* is introduced as a new species based on the above discrepancies in morphology, phylogeny, sequences, and distributions as per the guidelines of Chethana et al. (2021).

***Leucoglossum durandii*** (Teng) S. Imai, Botanical Magazine Tokyo 56: 524 (1942), Figure 8

**Figure 8.**
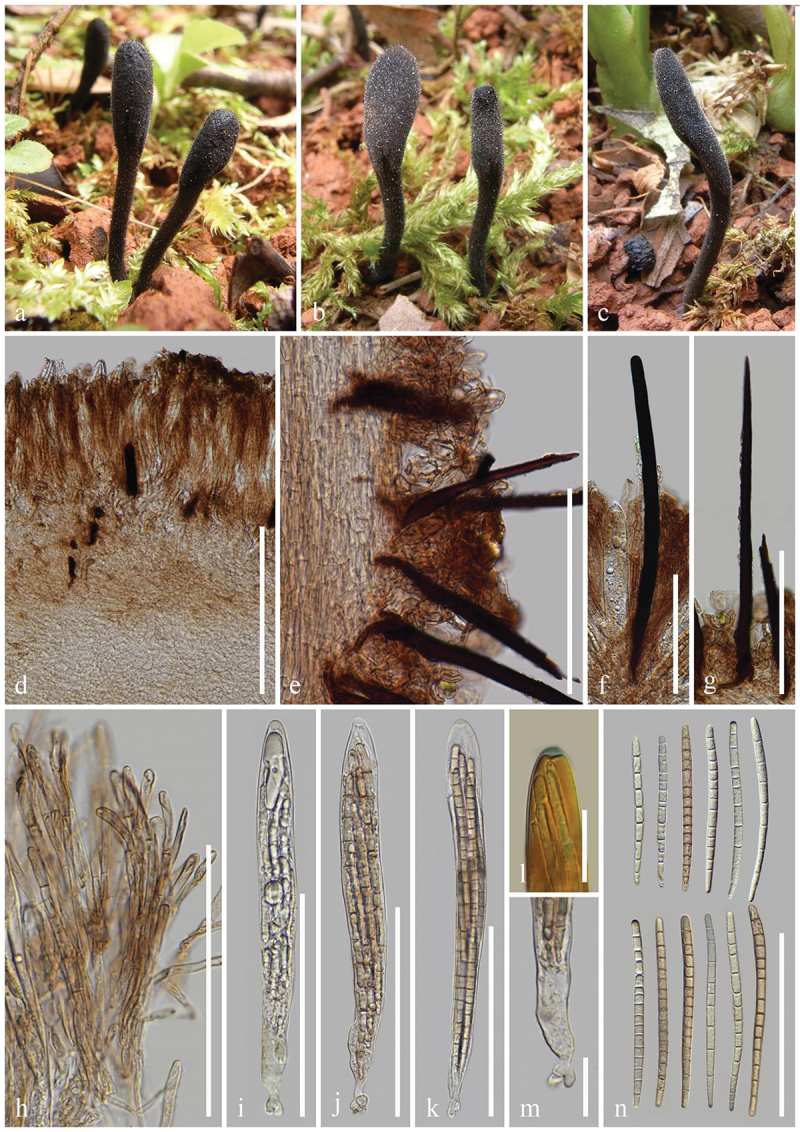
*Leucoglossum durandii* (HKAS 112842). (a–c) Fresh ascomata. (d) Longitudinal section of an ascigerous portion. (e) Longitudinal section of a stipe. (f, g) Setae. (h) Paraphyses. (i–k) Asci. (l) Ascus apex in MLZ. (m) Ascus base. (n) Ascospores. Scale bars: d = 200 µm, e–k, n = 100 µm, l, m = 20 µm.

*Index Fungorum number*: IF 287756; *Facesoffungi number*: FoF 17058.

*Description*: *Saprobic* on soil among moss. Sexual morph: *Ascomata* scattered, gregarious, tongue-shaped, stipitate, 2–3.5 cm in height, naturally curved, hirsute, with numerous setae. *Ascigerous portion* terete to elliptic, sometimes compressed, obtuse, 7–12 mm in height, 2–3 mm in diam., thicker than stipe, brownish black to black, concolourous with the stipe, surface cover white powder (ascospores). *Stipe* 7–20 mm in height, 1–1.5 mm in diam., cylindrical, slightly compressed, slightly curved, brownish black to black. *Hymenium* 170–220 µm ($\overset{ˉ}{x}$ = 198 µm, *n* = 27) thick, consisting of setae, paraphyses, and asci at different stages of development. *Ascigerous portion interior* brown, composed of septate, thin-walled hyphae of *textura intricate*, 3.3–7.3 µm ($\overset{ˉ}{x}$ = 5.1 µm, *n* = 51) in diam. *Stipe surface* 60–105 µm ($\overset{ˉ}{x}$ = 85 µm, *n* = 15) thick (without setae), composed of irregularly arranged, short, frequently septate, brown hyphae, with swollen, obtuse apical cells, 6.8–19 × 5–10.5 µm ($\overset{ˉ}{x}$ = 12 × 7.7 µm, *n* = 31). *Stipe interior* hyphae of *textura porrecta*, brown, composed of thin-walled hyphae, 2.7–6.3 µm ($\overset{ˉ}{x}$ = 4.1 µm, *n* = 56) in diam. *Setae* 190–710 × 9.8–21.7 µm ($\overset{ˉ}{x}$ = 479 × 13.7 µm, *n* = 57), heavily protruding above paraphyses and asci, acicular, most with sharp apex, sometimes with an obtuse apex, aseptate, dark brown to dark, thick-walled. *Paraphyses* filiform to thin-cylindrical, equal to asci, multi-septate, brown, lower portion 1.8–4 µm ($\overset{ˉ}{x}$ = 2.8 µm, *n* = 85) in diam., apical portion slightly curved, unswollen. *Asci* (170–)175–225(−230) × (14–)15–21(−22) µm ($\overset{ˉ}{x}$ = 196 × 18 µm, *n* = 72), 8-spored, cylindrical-clavate, attenuated towards the base, apically thick-walled, laterally thin-walled, with tapered, obtuse, amyloid apex, and croziers. *Ascospores* (70/4/1) (83–)91 –123(−132) × (4.1–)4.4–5.7(−6) µm ($\overset{ˉ}{x}$ = 107 × 5 µm, Q = 17.8–28, ***Q*** = 21.53 ± 2.73), mostly fasciculate, filiform to clavate, thin-walled, obtuse ends, straight to slightly curved, thin-walled, multi-guttulate, 8–15-septate, hyaline to light brown. Asexual morph: Not observed.

*Material examined*: China, Yunnan Province, Kunming, the Kunming Institute of Botany, on soil among moss in broad-leaf forests, 27 September 2020, Z.L. Yang, Yang 6446 (HKAS 112842); *ibid*., 17 September 2020, H.L. Su, SHL 9 (HKAS 137598).

*Notes*: The species is characterised by clavate, stipitate, black, hairy ascomata, an elliptic or lanceolate ascigerous portion covered with a powdery layer of ascospores, a cylindrical stipe, filiform paraphyses with slightly swollen, brownish, or subhyaline apical cells, and noticeably long (117–129 × 5–6 µm), clavate-cylindrical, 12–14-septate, hyaline to brownish ascospores (Tai 1944). Our two samples (HKAS 112842 and HKAS 137598) cluster with *Leucoglossum durandii* (HMAS 70090) as a lineage with 100% bootstrap and 1.00 Bayesian posterior probability within *Leucoglossum*. Based on these identical morphological characteristics and the very close phylogenetic relationship between our samples (HKAS 112842 and HKAS 137598) and *L. durandii* (HMAS 70090), we identify our two samples as *L. durandii*.

**Figure 9.**
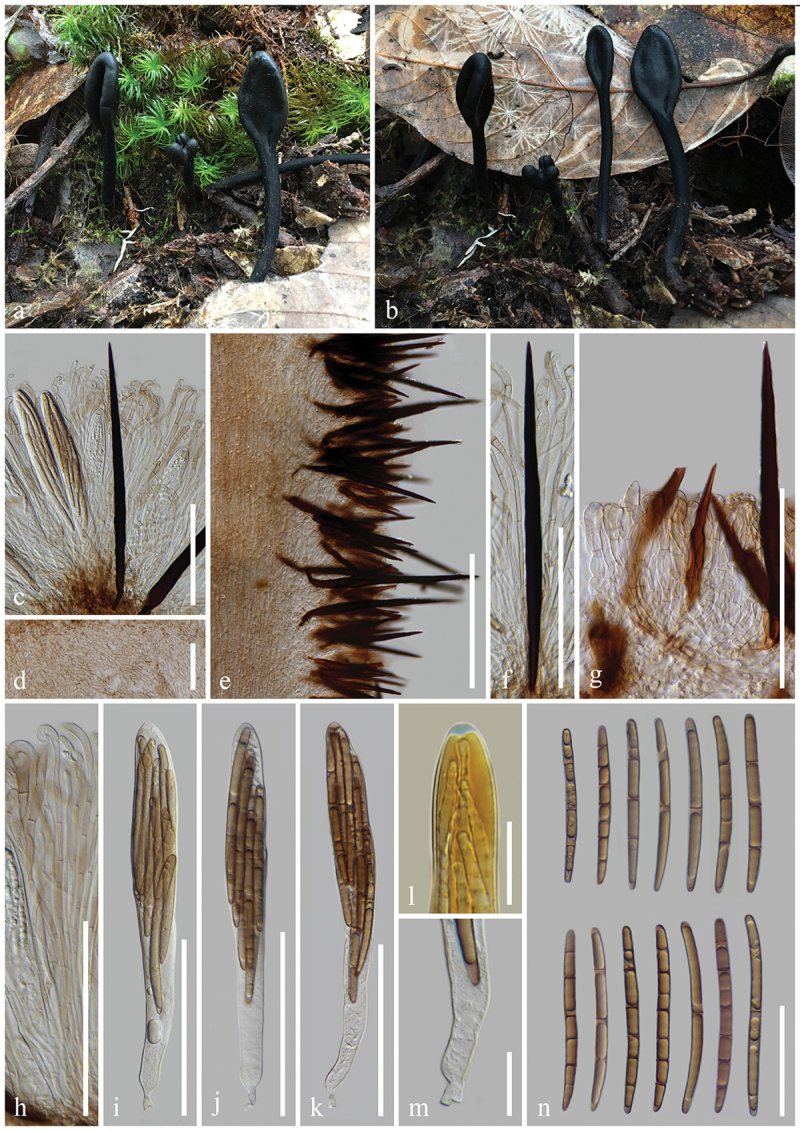
*Trichoglossum chuxiongense* (HKAS 137587, holotype). (a, b) Fresh ascomata. (c) Longitudinal section of an ascigerous portion. (d) Textura porrecta of ascigerous portion interior. (e, g) Longitudinal section of stipe surface with setae. (f) Seta of hymenium. (h) Paraphyses. (i–k) Asci. (l) Ascus apex in MLZ. (m) Ascus base. (n) Ascospores. Scale bars: c, f–k = 100 µm, d, n = 50 µm, e = 200 µm, l, m = 20 µm.

***Trichoglossum chuxiongense*** H.L. Su, K.D. Hyde, Zhu L. Yang & Q. Zhao, sp. nov., Figure 9

*Index Fungorum number*: IF 903122; *Facesoffungi number*: FoF 17000.

*Etymology*: Referring to the type locality Chuxiong, Yunnan, China.

*Holotype*: HKAS 137587.

*Diagnosis*: *Trichoglossum chuxiongense* is similar to *T. farlowii* in ecology, macromorphology, and paraphyses, but distinctly differs from the latter by its asci and ascospores.

*Description*: *Saprobic* on soil among moss. Sexual morph: *Ascomata* scattered, gregarious, cylindrical, stipitate, 5–10 cm in height, straight to slightly curved, hirsute, with numerous setae. *Ascigerous portion* swollen, flattened lanceolate or flexuous, more or less compressed, with obtuse apex, 10–25 mm in height, 10–15 mm in diam., black, concolourous with the stipe, densely hirsute from setae. *Stipe* 40–70 mm in height, 3–5 mm in diam., cylindrical, compressed, straight to slightly curved, sometimes branch, densely hirsute from setae. *Hymenium* 190–250 µm ($\overset{ˉ}{x}$ = 217 µm, *n* = 33) thick (without setae), consisting of setae, paraphyses, and asci at different stages of development. *Ascigerous portion interior* light brown to hyaline, composed of septate, thin-walled hyphae of *textura porrecta* 1.7–5 µm ($\overset{ˉ}{x}$ = 3.2 µm, *n* = 103) in diam. *Stipe surface* 90–170 µm ($\overset{ˉ}{x}$ = 127 µm, *n* = 18) thick (without setae), composed of hyaline to light brown hyphae, frequently septate, 5–12.7 µm ($\overset{ˉ}{x}$ = 7.8 µm, *n* = 60) in diam., arranged parallel to setae. *Stipe interior* cells of *textura porrecta*, brown, composed of thin-walled hyphae, 2.7–11 µm ($\overset{ˉ}{x}$ = 5.1 µm, *n* = 150) in diam. *Setae* 107–344 × 7–19.3 µm ($\overset{ˉ}{x}$ = 213 × 11 µm, *n* = 97), heavily protruding above paraphyses and asci, acicular, with sharp apex, aseptate, dark brown to black, thick-walled. *Paraphyses* filiform, equal to asci, multi-septate, hyaline to light brown, lower portion 1.5–4 µm ($\overset{ˉ}{x}$ = 2.5 µm, *n* = 63) in diam., apical portion curved, swollen, 3.3–10.8 µm ($\overset{ˉ}{x}$ = 5.7 µm, *n* = 139). *Asci* 205–235(−240) × 17–26(−27) µm ($\overset{ˉ}{x}$ = 219 × 21 µm, *n* = 46), 8-spored, cylindrical-clavate, attenuated towards the base, apically thick-walled, laterally thin-walled, with tapered, obtuse, amyloid apex, and croziers. *Ascospores* (214/4/1) (61–)70–93(−104) × (4.1–)4.8–6.5(−7.2) µm ($\overset{ˉ}{x}$ = 83 × 5.6 µm, Q = 10.5–21.8, ***Q*** = 14.99 ± 1.65), fasciculate, filiform to clavate, obtuse ends, straight to slightly curved, thin-walled, multi-guttulate, mostly 8-guttulate, 1–6-septate, mostly 6-septate, brown. Asexual morph: Not observed.

*Material examined*: China, Yunnan Province, Chuxiong, Nanhua, on soil among moss in mixed deciduous forests, 16 August 2017, J. Wang, WJ222 (HKAS 137587, **holotype**); *ibid*., 2 September 2021, H.L. Su, SHL 183 (HKAS 137599, **paratype**).

*Notes*: Our multi-loci phylogenetic tree shows that *Trichoglossum chuxiongense* (HKAS 23 and HKAS 183) clusters with *T. farlowii* (ZW-persomal collection and Inat 15754448) with 97% bootstrap and 1.00 Bayesian posterior probability. *Trichoglossum chuxiongense* and *T. farlowii* share the same habitat and live on soil in mixed deciduous forests. Moreover, the two species share similar tongue-shaped ascomata, compressed ascogenous portion, terete stipe, and cylindric paraphyses with curved, swollen apex. However, *T. chuxiongense* differs from *T. farlowii* in asci and ascospores. *Trichoglossum chuxiongense* has larger asci (205–240 × 17–27 µm vs. 150–180 × 15–20 µm) than those of *T. farlowii*. Additionally, *T. chuxiongense* produces mostly 6-septate, dark brown ascospores, whereas *T. farlowii* bears mostly 3-septate, light brown ascospores (Mains 1954). Sequence comparison of the ITS regions between *T. chuxiongense* (HKAS 137587) and *T. farlowii* (ZW-personal collection) reveals 21 base pair differences, including 4 gaps, across 493 base pairs (21/493–4.2%) (Wang et al. 2011; Jeewon and Hyde 2016). According to these differences in molecular phylogeny, morphology, and sequence comparisons, *T. chuxiongense* is introduced as a new species following the guidelines of Chethana et al. (2021).

**Figure 10.**
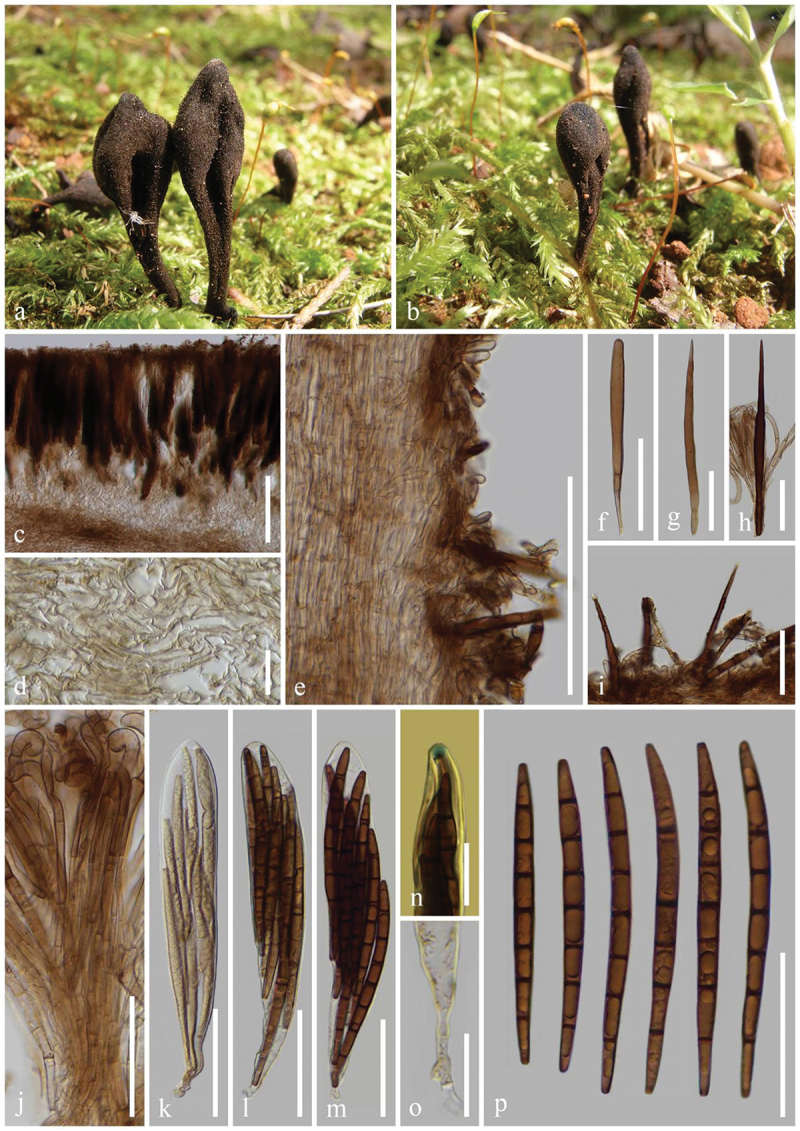
*Trichoglossum conica* (HKAS 112867, holotype). (a, b) Fresh ascomata. (c) Longitudinal section of an ascigerous portion. (d) Textura intricate of ascigerous portion interior. (e) Longitudinal section of a stipe. (f–h) Setae of hymenium. (i) Setae of stipe. (j) Paraphyses. (k–m) Asci. (n) Ascus apex in MLZ. (o) Ascus base. (p) Ascospores. Scale bars: c, e = 100 µm, f–m, p = 50 µm, d, n, o = 20 µm.

***Trichoglossum conica*** H.L. Su, K.D. Hyde, Zhu L. Yang & Q. Zhao, sp. nov., Figure 10

*Index Fungorum number*: IF 903123; *Facesoffungi number*: FoF 17001.

*Etymology*: Referring to mature ascigerous portion with tapered apex.

*Holotype*: HKAS 112867.

*Diagnosis*: *Trichoglossum conica* resembles *T. variabile* and *T. hirsutum* but can be distinguished from the latter two by its ascospores.

*Description*: *Saprobic* on soil among moss. Sexual morph: *Ascomata* caespitose to scattered, gregarious, clavate, stipitate, 1–4.5 cm in height, hirsute, with numerous setae. *Ascigerous portion* swollen, capitate, conical, or oval, mostly with tapered, obtuse apex, irregularly dented, 4–10 mm in height, 3–8 mm in diam., dark, concolorous with the stipe, densely hirsute from setae. *Stipe* 5–40 mm in height, 1–2.5 mm in diam., cylindrical, straight to slightly slanted, densely hirsute from setae. *Hymenium* 195–240 µm ($\overset{ˉ}{x}$ = 215 µm, *n* = 20) thick (without setae), consisting of setae, paraphyses, and asci at different stages of development. *Ascigerous portion interior* brown, composed of thin-walled hyphae of *textura intricate*, 1.7–6.5 µm ($\overset{ˉ}{x}$ = 3.5 µm, *n* = 94) in diam. *Stipe surface* 20–60 µm ($\overset{ˉ}{x}$ = 39 µm, *n* = 22) thick (without setae), composed of irregularly arranged, short, frequently septate, light brown hyphae, with slightly swollen, obtuse apical cells, 8.5–23.5 × 4.5–11 µm ($\overset{ˉ}{x}$ = 13.5 × 7 µm, *n* = 42). *Stipe interior* hyphae of *textura porrecta*, light brown, composed of thin-walled hyphae, 2.8–5.7 µm ($\overset{ˉ}{x}$ = 4 µm, *n* = 109) in diam. *Setae* (95–)100–225(−295) × (4.5–)5–12 (−14.5) µm ($\overset{ˉ}{x}$ = 168 × 8.5 µm, *n* = 22), heavily protruding above paraphyses and asci, acicular, most with sharp apex, sometimes with obtuse apex, mostly aseptate, sometimes 1-septate, light brown to dark brown, thick-walled. *Paraphyses* filiform to cylindrical, protruding above the asci, multi-septate, pale brown, lower portion 1.5–4 µm ($\overset{ˉ}{x}$ = 2.5 µm, *n* = 111) in diam., apical portion curved, swollen, 3.5–7.5 µm ($\overset{ˉ}{x}$ = 5.3 µm, *n* = 63) in diam. *Asci* (164–)168–203(−217) × (14–)18–29(−33) µm ($\overset{ˉ}{x}$ = 184 × 23 µm, *n* = 57), 8-spored, cylindrical-clavate, attenuated towards the base, apically thick-walled, laterally thin-walled, with tapered, obtuse, amyloid apex, and croziers. *Ascospores* (149/4/1) (86–)92–113(−117) × (5–)5.4–7.3(−8) µm ($\overset{ˉ}{x}$ = 103 × 6.3 µm, Q = 12.3–21.6, ***Q*** = 16.5 ± 1.67), fasciculate, mostly filiform to cylindrical, with slightly swollen middle, and tapered, obtuse ends, straight to slightly curved, thick-walled, mostly 8-guttulate, 5–7-septate, dark brown. Asexual morph: Not observed.

*Material examined*: China, Yunnan Province, Kunming, the Kunming Institute of Botany, on soil among moss, 11 October 2020, Z.L. Yang, Yang 6471 (HKAS 112867, **holotype**); *ibid*., 26 September 2020, Z.L. Yang, Yang 6445 (HKAS 112841, **paratype**).

*Notes*: Our phylogenetic analyses show that *Trichoglossum conica* (HKAS 112867 and HKAS 112841) nests within the *Trichoglossum*-II clade. *Trichoglossum conica* (HKAS 112867 and HKAS 112841) clusters with the clade including *T. variabile* (Carmel Sammut CS1378, ERRO 2009111802, and ERRO 2011012206) and *T. hirsutum* (ILLS 67355, ILLS 61045, Jamie Platt JP267, and OSC 61726) with 36% bootstrap and 0.95 Bayesian posterior probability. *Trichoglossum conica*, *T. hirsutum,* and *T. variabile* have different habitats. *Trichoglossum conica* and *T. hirsutum* both live on soil among moss, while *T. variabile* lives on rotten wood or humus among leaves (Persoon 1794; Durand 1908a). Moreover, the three species have similar clavate to capitate ascomata and asci. However, they are different in macro-morphological characteristics. *Trichoglossum conica* and *T. variabile* have caespitose ascomata, while *T. hirsutum* has scattered or gregarious ascomata. In addition, these three species have different ascospores, *T. conica* has 5–7-septate ascospores, while *T. hirsutum* has 13–17-septate, mostly 15-septate ones, and *T. variabile* has mostly 10–13-septate ones (Prabhugaonkar and Pratibha 2017; Dasgupta et al. 2022). Furthermore, these three species have distinctly different ITS and LSU sequences, which are compared in Table 3. Therefore, *T. conica* is introduced as a new species, supported by comprehensive analyses of its morphology, phylogeny, and ecology following the guidelines of Chethana et al. (2021).

**Table 3.** Comparison of nucleotide differences among *Trichoglossum conica* (HKAS 112867), *T. variabile* (ERRO 2009111802, Carmel Sammut CS1378, and ERRO 2011012206), and *T. hirsutum* (ILLS 67355, ILLS 61045, Jamie Platt JP267, and OSC 61726) sequences.

Taxa for comparison	ITS	LSU
*T. conica* (HKAS 112867) and *T. variabile* (ERRO 2009111802)	92/601–15.3% (56 gaps)	-
*T. conica* (HKAS 112867) and *T. variabile* (Carmel Sammut CS1378)	97/606–16% (59 gaps)	-
*T. conica* (HKAS 112867) and *T. variabile* (ERRO 2011012206)	88/581–15% (55 gaps)	-
*T. conica* (HKAS 112867) and *T. hirsutum* (ILLS 67355)	50/412–12.1% (25 gaps)	27/1000–2.7% (9 gaps)
*T. conica* (HKAS 112867) and *T. hirsutum* (ILLS 61045)	35/409–8.6% (23 gaps)	27/1000–2.7% (9 gaps)
*T. conica* (HKAS 112867) and *T. hirsutum* (Jamie Platt JP267)	-	18/848–2.1% (3 gaps)
*T. conica* (HKAS 112867) and *T. hirsutum* (OSC 61726)	92/626–14.7% (55 gaps)	19/864–2.2% (4 gaps)

**Figure 11.**
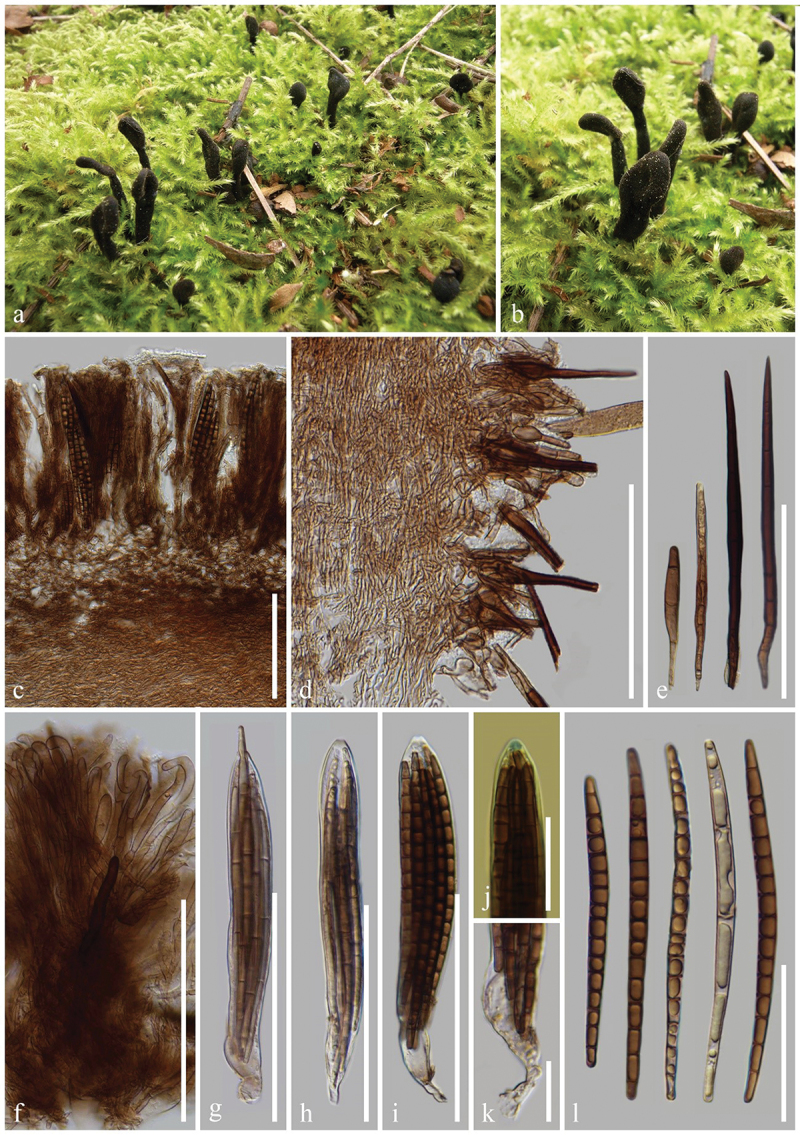
*Trichoglossum distortus* (HKAS 112863, holotype). (a, b) Fresh ascomata. (c) Longitudinal section of an ascigerous portion. (d) Longitudinal section of a stipe with setae. (e) Setae of hymenium. (f) Paraphyses. (g–i) Asci. (j) Ascus apex in MLZ. (k) Ascus base. (l) Ascospores. Scale bars: c–i = 100 µm, j, k = 20 µm, l = 50 µm.

***Trichoglossum distortus*** H.L. Su, K.D. Hyde, Zhu L. Yang & Q. Zhao, sp. nov., Figure 11

*Index Fungorum number*: IF 903124; *Facesoffungi number*: FoF 17002.

*Etymology*: Referring to the mostly curved ascomata.

*Holotype*: HKAS 112863.

*Diagnosis*: *Trichoglossum distortus* is similar to *T. subhirsutum*, but differs from the latter by its shorter ascomata, narrower ascigerous portion, shorter and thicker stipe, and shorter asci.

*Description*: *Saprobic* on soil among moss. Sexual morph: *Ascomata* scattered to gregarious, or clustered, clavate, stipitate, 1.5–2.5 cm in height, mostly curved, hirsute, with numerous setae. *Ascigerous portion* swollen, flattened lanceolate, or swollen triangular, with severe central depressions, 3–6 mm in height, 3–5 mm wide, black, concolourous with the stipe, densely hirsute from setae. *Stipe* 15–20 mm in height, 1.5–2.5 mm wide, cylindrical, slightly curved, densely hirsute from setae. *Hymenium* 170–235 µm ($\overset{ˉ}{x}$ = 201 µm, *n* = 27) thick (without setae), consisting of setae, paraphyses, and asci at different stages of development. *Ascigerous portion interior* brown, composed of septate, thin-walled hyphae of *textura porrecta*, 2.2–6.7 µm ($\overset{ˉ}{x}$ = 3.8 µm, *n* = 103) in diam. *Stipe surface* 49–72 µm ($\overset{ˉ}{x}$ = 63 µm, *n* = 18) thick (without setae), brown, thick-walled hyphae of *textura intricate*, frequently septate, 1.6–3.6 µm ($\overset{ˉ}{x}$ = 2.6 µm, *n* = 88) in diam. *Stipe surface* 49–72 µm ($\overset{ˉ}{x}$ = 63 µm, *n* = 18) thick (without setae), composed of brown, thick-walled hyphae, frequently septate, 2.8–5.8 µm ($\overset{ˉ}{x}$ = 4.3 µm, *n* = 28) in diam., arranged parallel to setae. *Stipe interior* hyphae of *textura intricata*, brown, composed of thick-walled hyphae, 1.4–6 µm ($\overset{ˉ}{x}$ = 3.3 µm, *n* = 126) in diam. *Setae* 55–190 × 4.5–10.5 µm ($\overset{ˉ}{x}$ = 107 × 7 µm, *n* = 25), equal to paraphyses and asci, acicular, with sharp or obtuse apex, septate, brown to dark brown, some with swollen bases. *Paraphyses* filiform, slightly protruding above the asci, multi-septate, brown or dark brown, lower portion 1.5–5.3 µm ($\overset{ˉ}{x}$ = 2.8 µm, *n* = 160) in diam., apical portion curved, slightly swollen, 2.5–6.5 µm ($\overset{ˉ}{x}$ = 4.4 µm, *n* = 63) in diam. *Asci* (154–)161–189(−192) × 19–26(−28) µm ($\overset{ˉ}{x}$ = 179 × 24 µm, *n* = 23), 8-spored, cylindrical-clavate, attenuated towards the base, apically thick-walled, laterally thin-walled, with tapered, obtuse, amyloid apex, and croziers. *Ascospores* (78/4/1) (96–)101–129(−135) × (5–)5.4–7(−7.4) µm ($\overset{ˉ}{x}$ = 114 × 6.2 µm, Q = 14.7–24.8, ***Q*** = 18.5 ± 1.93), fasciculate, filiform, obtuse ends, straight to slightly curved, thin-walled, multi-guttulate, 10–16-septate, mostly 15-septate, mostly brown, sometimes light brown. Asexual morph: Not observed.

*Material examined*: China, Yunnan Province, Kunming, the Kunming Institute of Botany, on soil among moss in broad-leaf forests, 7 January 2020, Z.L. Yang, Yang 6467 (HKAS 112863, **holotype**); *ibid*., 22 September 2020, H.L. Su, SHL81 (HKAS 137594, **paratype**).

*Notes*: In the multi-loci phylogenetic tree, *Trichoglossum distortus* (HKAS 112863 and HKAS 137594) forms a clade separated from *T. subhirsutum* (HKAS 112832, HKAS 137597, HKAS 112850, and HKAS 69042) with 29% bootstrap and 0.85 Bayesian posterior probability. *Trichoglossum distortus* and *T. subhirsutum* have a similar habitat and live on soil among moss in broad-leaf forests. In addition, *T. distortus* is similar to *T. subhirsutum* in their setae, paraphyses, and ascospores. However, *T. distortus* can be distinguished from *T. subhirsutum* by its shorter ascomata (1.5–2.5 cm vs. 2.5–6 cm), narrower ascigerous portion (3–5 mm vs. 5–10 mm), shorter stipe (15–20 mm vs. 20–50 mm), and shorter asci (154–192 × 19–28 µm vs. 195–232 × 16–30 µm). In addition, *T. distortus* (HKAS 112863) and *T. subhirsutum* (HKAS 137597) have significant differences in their ITS regions (41 base pairs with 28 gaps; 41/400–10.3%) and their LSU regions (6 base pairs; 6/830–0.7%) (Jeewon and Hyde 2016). Moreover, *T. distortus* shares similar macromorphological characteristics with *T. benghalense*, *T. confusum*, *T. kunmingense*, *T. octopartitum*, *T. peruvianum*, *T. qingchengense*, *T. rehmianum*, *T. septatum*, and *T. tropicale*. However, *T. distortus* differs from *T. confusum*, *T. kunmingense*, *T. octopartitum*, *T. peruvianum*, *T. qingchengense*, *T. rehmianum*, and *T. septatum* in its asci and ascospores, as shown in Table 7. *Trichoglossum distortus* also differs from *T. tropicale* in the amyloid reaction of asci and paraphyses; *T. distortus* has amyloid asci and slightly swollen paraphysis apices, while *T. tropicale* has inamyloid asci and obviously swollen apex of paraphyses (2.5–6.5 µm vs. 8–46 µm) (de la Fuente et al. 2022). Furthemore, *T. distortus* and *T. tropicale* are distantly related in our phylogenetic tree (Figure 1). Based on this morphology, phylogeny, and sequence comparison, a new taxon, *T. distortus*, is proposed following the guidelines of Chethana et al. (2021).

**Table 4.** Comparison of nucleotide differences among *Trichoglossum hirsutum* (HKAS 55133, HKAS 137586, HKAS 137593, and HKAS 137595) sequences.

Taxa for comparison	ITS	LSU
*T. hirsutum* (HKAS 55133) and *T. hirsutum* (HKAS 137586)	5/542–0.92% (3 gaps)	1/987–0.1%
*T. hirsutum* (HKAS 55133) and *T. hirsutum* (HKAS 137593)	12/552–2.2% (4 gaps)	1/984–0.01%
*T. hirsutum* (HKAS 55133) and *T. hirsutum* (HKAS 137595)	12/555–2.2% (4 gaps)	2/966–0.2%

***Trichoglossum hirsutum*** (Pers.) Boud., Histoire et Classification des Discomycètes d’Europe: 86 (1907), Figure 12

**Figure 12.**
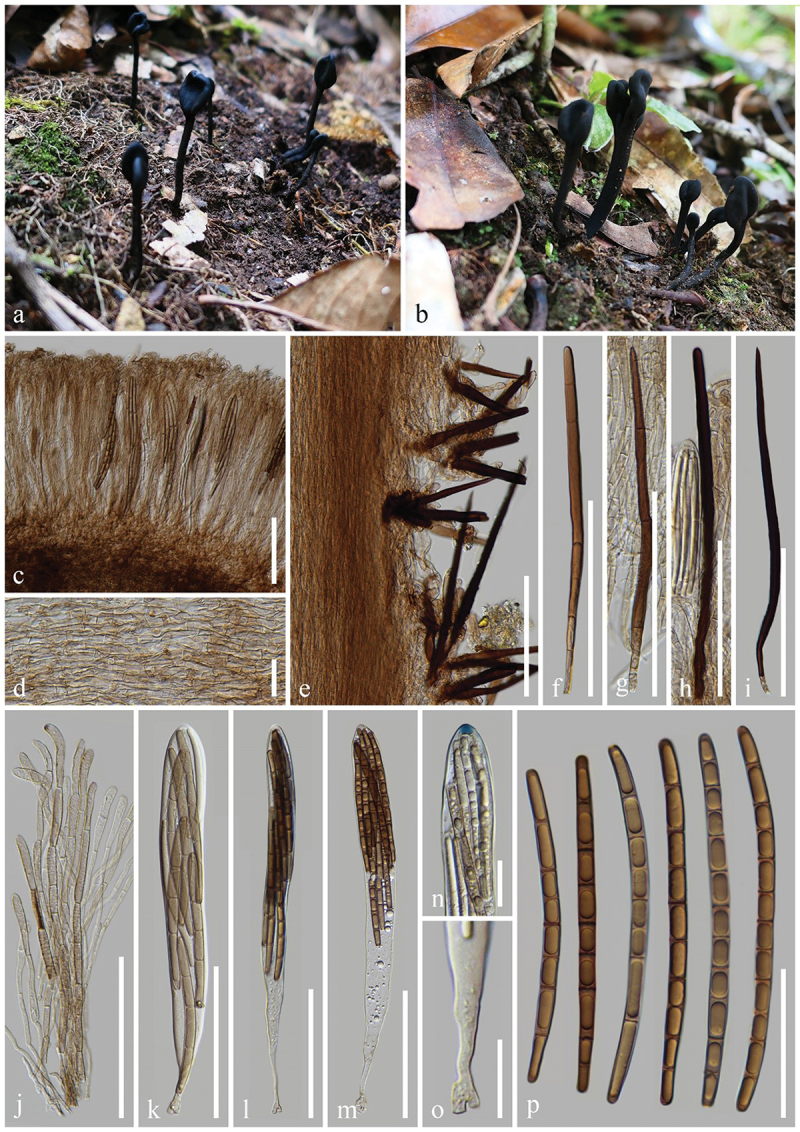
*Trichoglossum hirsutum* (HKAS 137595). (a, b) Fresh ascomata. (c) Longitudinal section of an ascigerous portion. (d) Textura porrecta of ascigerous portion interior. (e) Longitudinal section of a stipe surface with setae. (f–i) Setae of hymenium. (j) Paraphyses. (k–m) Asci. (n) Ascus apex in MLZ. (o) Ascus base. (p) Ascospores. Scale bars: c, e–m = 100 µm, d, n, o = 20 µm, p = 50 µm.

*Index Fungorum number*: IF 187796; *Facesoffungi number*: FoF 17059.

*Description*: *Saprobic* on soil among moss. Sexual morph: *Ascomata* scattered, gregarious, sometimes clustered, capitate, stipitate, 2.5–5 cm in height, hirsute, with numerous setae. *Ascigerous portion* flattened lanceolate, spatulate, irregularly folded, or triangular with severe central depressions, 5–25 mm in height, 2–11 mm wide, black, concolourous with the stipe, densely hirsute from setae. *Stipe* 20–40 mm in height, 0.8–2 mm wide, cylindrical, straight to slightly curved, black, densely hirsute from setae. *Hymenium* 170–315 µm ($\overset{ˉ}{x}$ = 220 µm, *n* = 23) thick (without setae), consisting of setae, paraphyses, and asci at different stages of development. *Ascigerous portion interior* light brown, composed of septate, thin-walled hyphae of *textura porrecta*, 2.7–8.1 µm ($\overset{ˉ}{x}$ = 5.3 µm, *n* = 102) in diam. *Stipe surface* 25–95 µm ($\overset{ˉ}{x}$ = 49 µm, *n* = 18) thick (without setae), composed of irregularly arranged, long, septate, hyaline to light brown hyphae, 3.3–10.5 µm ($\overset{ˉ}{x}$ = 5.6 µm, *n* = 59) in diam. *Stipe interior* hyphae of *textura porrecta*, brown, composed of thick-walled hyphae, 2.4–8.7 µm ($\overset{ˉ}{x}$ = 5.7 µm, *n* = 138) in diam. *Setae* 95–265 × 4–13 µm ($\overset{ˉ}{x}$ = 168 × 8.1 µm, *n* = 21), equal to paraphyses and asci, acicular, with sharp apex, sometimes with obtuse apex, septate, brown to dark brown. *Paraphyses* filiform, protruding above the asci, multi-septate, light brown or hyaline, lower portion 1.8–5.8 µm ($\overset{ˉ}{x}$ = 3.4 µm, *n* = 126) in diam., apical portion slightly curved, generally unswollen. *Asci* (235–)245–295(−300) × (17–)19–30(−35) µm ($\overset{ˉ}{x}$ = 272 × 24 µm, *n* = 73), 8-spored, cylindrical-clavate, attenuated towards the base, apically thick-walled, laterally thin-walled, with tapered, obtuse, amyloid apex, and have croziers. *Ascospores* (131/4/1) (101–)105–128(−134) × (5–)5.2–7.1(−7.4) µm ($\overset{ˉ}{x}$ = 118 × 6.1 µm, Q = 14.8–26, ***Q*** = 19.51 ± 2.13), fasciculate, filiform, obtuse ends, straight to slightly curved, thin-walled, multi-guttulate, 7–11-septate, light brown to brown. Asexual morph: Not observed.

*Material examined*: China, Yunnan Province, Yuxi, Ailao Mountains, on soil among moss in broad-leaf forest, 2 September 2021, H.L. Su, SHL191 (HKAS 137595); *ibid*., 2 September 2021, H.L. Su, SHL196 (HKAS 137593); China, Yunnan Province, Wenshan, Laojun Mountains, on soil among moss, 18 October 2017, J. Wang, WJ403 (HKAS 137586).

*Notes*: *Trichoglossum hirsutum* is distinguished by clavate to capitate, stipitate, black, hairy ascomata, a clavate to lanceolate ascigerous portion, a cylindrical stipe, acuminate, brown to blackish brown setae, filiform paraphyses with an unswollen, slightly curved apex, and fusiform, 13–17-septate, brown ascospores (Prabhugaonkar and Pratibha 2017; Kaygusuz et al. 2020; Dasgupta et al. 2022). Our three samples (HKAS 137586, HKAS 137593, and HKAS 137595) are morphologically similar to *T. hirsutum* but differ in ascospore septation, our three samples have 7–11-septate ascospores, whereas *Trichoglossum hirsutum* has 13–17-septate ascospores. In our phylogenetic analyses, our three samples (HKAS 137586, HKAS 137593, and HKAS 137595) form a clade with *T. hirsutum* (HKAS 55133) with 100% bootstrap and 1.00 Bayesian posterior probability. Furthermore, comparing the sequences of our three samples (HKAS 137586, HKAS 137593, and HKAS 137595) and *T. hirsutum* (HKAS 55133), only minor differences were found in the ITS and LSU regions (Table 4) (Jeewon and Hyde 2016). Based on the comprehensive research results, we identify our three samples as *T. hirsutum* and attribute the differences in ascospore septation to intraspecific variation within the species.

**Table 5.** Comparison of nucleotide differences among *Trichoglossum subhirsutum* (HKAS 112832) and *T. hirsutum* (ILLS 67355, ILLS 61045, Jamie Platt JP267, OSC 61726, HKAS 137586, HKAS 55133, HKAS 137593, and HKAS 137595) sequences.

Taxa for comparison	ITS	LSU
*T. subhirsutum* (HKAS 112832) and *T. hirsutum* (ILLS 67355)	61/618–9.9% (22 gaps)	123/1005–12.2% (21 gaps)
*T. subhirsutum* (HKAS 112832) and *T. hirsutum* (ILLS 61045)	66/619–10.7% (25 gaps)	123/1005–12.2% (21 gaps)
*T. subhirsutum* (HKAS 112832) and *T. hirsutum* (Jamie Platt JP267)	-	109/834–13.1% (13 gaps)
*T. subhirsutum* (HKAS 112832) and *T. hirsutum* (OSC 61726)	79/615–12.8% (39 gaps)	111/855–13.0% (14 gaps)
*T. subhirsutum* (HKAS 112832) and *T. hirsutum* (HKAS 137586)	83/585–14.2% (40 gaps)	107/990–10.8% (12 gaps)
*T. subhirsutum* (HKAS 112832) and *T. hirsutum* (HKAS 55133)	88/602–14.6% (44 gaps)	107/997–10.7% (12 gaps)
*T. subhirsutum* (HKAS 112832) and *T. hirsutum* (HKAS 137593)	87/604–14.4% (41 gaps)	107/981–10.9% (12 gaps)
*T. subhirsutum* (HKAS 112832) and *T. hirsutum* (HKAS 137595)	87/601–14.5% (41 gaps)	106/965–11.0% (12 gaps)

***Trichoglossum rasum*** Pat., Bull. Soc. mycol. Fr. 25: 130 (1909), Figure 13

**Figure 13.**
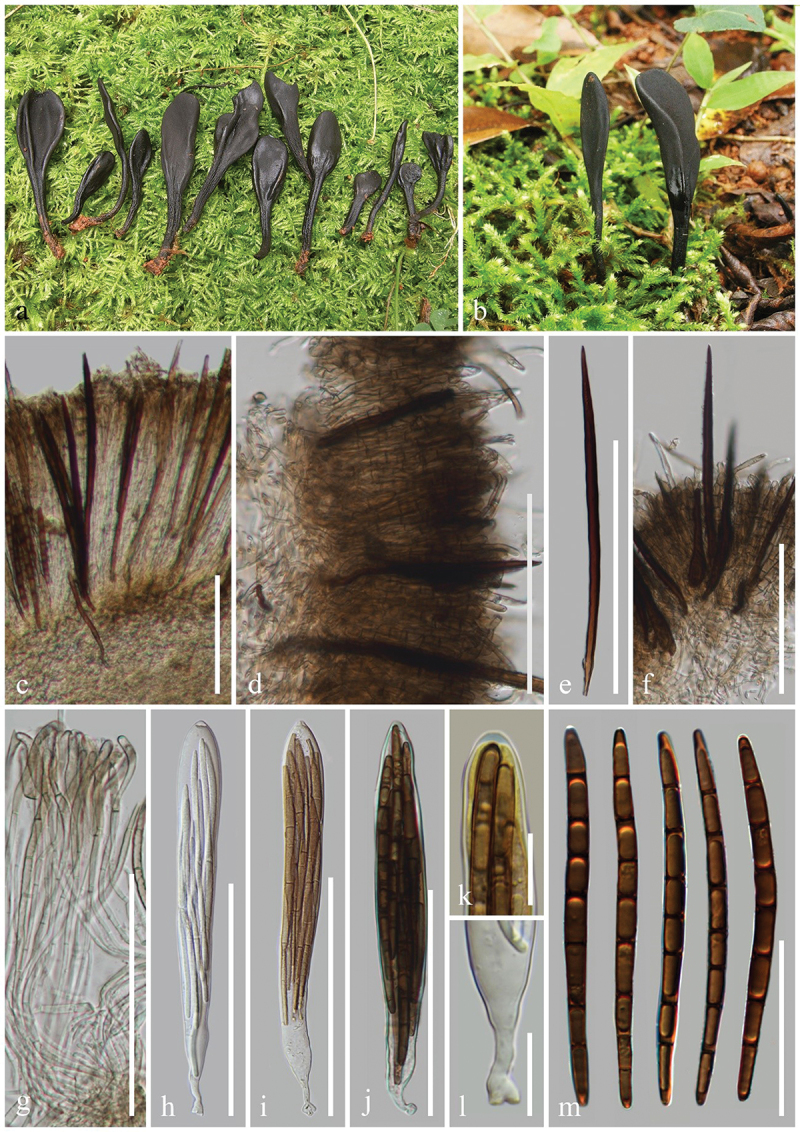
*Trichoglossum rasum* (HKAS 112847). (a, b) Fresh ascomata. (c) Longitudinal section of an ascigerous portion. (d) Longitudinal section of a stipe. (e) Setae of hymenium. (f) Setae of stipe. (g) Paraphyses. (h–j) Asci. (k) Ascus apex in MLZ. (l) Ascus base. (m) Ascospores. Scale bars: c–j = 100 µm, k, l = 20 µm, m = 50 µm.

*Index Fungorum number*: IF 183730; *Facesoffungi number*: FoF 17060.

*Description: Saprobic* on soil among moss. Sexual morph: *Ascomata* gregarious, scattered, clavate to spatulate, tongue-shaped, stipitate, 2–5 cm in height, hirsute, with numerous setae. *Ascigerous portion* swollen, compressed, obtuse, 15–40 mm in height, 5–10 mm wide, obviously wider than stipe, dark brown to black, concolourous with the stipe. *Stipe* 10–20 mm in height, 2–5 mm in diam., cylindrical, more or less compressed, straight to slightly curved. *Hymenium* 190–230 µm ($\overset{ˉ}{x}$ = 213 µm, *n* = 21) thick (without setae), consisting of setae, paraphyses, and asci at different stages of development. *Ascigerous portion interior* light brown to hyaline, composed of septate, thin-walled hyphae of *textura intricate*, 3.5–6.6 µm ($\overset{ˉ}{x}$ = 4.9 µm, *n* = 48) in diam. *Stipe surface* 90–135 µm ($\overset{ˉ}{x}$ = 108 µm, *n* = 15) thick (without setae), dark brown, septate hyphae, 4.5–8.5 µm ($\overset{ˉ}{x}$ = 6.1 µm, *n* = 52) in diam., arranged parallel to setae. *Stipe interior* hyphae of *textura intricate*, light brown, composed of thin-walled hyphae, 1.6–3 µm ($\overset{ˉ}{x}$ = 2.3 µm, *n* = 56) in diam. *Setae* 100–235 × 4–10 µm ($\overset{ˉ}{x}$ = 154 × 6.8 µm, *n* = 22), heavily protruding above paraphyses and asci, acicular, with sharp apex, mostly aseptate, dark brown to black, thick-walled. *Paraphyses* filiform, equal to asci, multi-septate, lower portion hyaline, 1.8–4.6 µm ($\overset{ˉ}{x}$ = 2.9 µm, *n* = 39) in diam., apical portion slightly curved, slightly swollen, light brown, 4.2–5.9 µm ($\overset{ˉ}{x}$ = 4.9 µm, *n* = 32) in diam. *Asci* (195–)205–235(−245) × (15–)17–25(−27) µm ($\overset{ˉ}{x}$ = 223 × 23 µm, *n* = 35), 8-spored, cylindrical-clavate, attenuated towards the base, apically thick-walled, laterally thin-walled, with tapered, obtuse, amyloid apex, and croziers. *Ascospores* (72/3/3) (93–)108–118(−122) × (5.6–)6–6.2(−6.5) µm ($\overset{ˉ}{x}$ = 109 × 6 µm, Q = 16.6–18.5, ***Q*** = 18 ± 2.43), fasciculate, filiform, obtuse ends, straight to slightly curved, thin-walled, multi-guttulate, 5–8-septate, mostly 7-septate, brown. Asexual morph: Not observed.

*Material examined*: China, Yunnan Province, Kunming, the Kunming Institute of Botany, on soil among moss in broadleaf forests, 13 August 2016, J.W. Liu, LJW552 (HKAS 98131); *ibid*., 24 September 2020, Z.L. Yang, Yang 6438 (HKAS 112834); *ibid*., 25 September 2020, Z.L. Yang, Yang 6440 (HKAS 112836); *ibid*., 29 September 2020, Z.L. Yang, Yang 6451 (HKAS 112847); *ibid*., 29 September 2020, Z.L. Yang, Yang 6453 (HKAS 112849); *ibid*., 30 September 2020, Z.L. Yang, Yang 6457 (HKAS 112853); *ibid*., 30 September 2020, Z.L. Yang, Yang 6458 (HKAS 112854).

*Notes*: The species is characterised by clavate to broadly spathulate, stipitate, black, hairy ascomata, a flattened ascigerous portion, a cylindrical stipe, filiform paraphyses with slightly swollen, brownish, or subhyaline apical cells, and fusoid-clavate to fusoid, mostly 6–9-septate, brown ascospores (Mains 1954; Dasgupta et al. 2022). Our three samples (HKAS 98131, HKAS 112849, and HKAS 112853) cluster with *Trichoglossum rasum* (HCIO 52051) as a lineage with 99% bootstrap and 1.00 Bayesian posterior probability. Based on these similar morphological characteristics and the very close phylogenetic relationship between our three samples (HKAS 98131, HKAS 112849, and HKAS 112853) and *T. rasum* (HCIO 52051), we identify our three samples as *T. rasum*.

**Figure 14.**
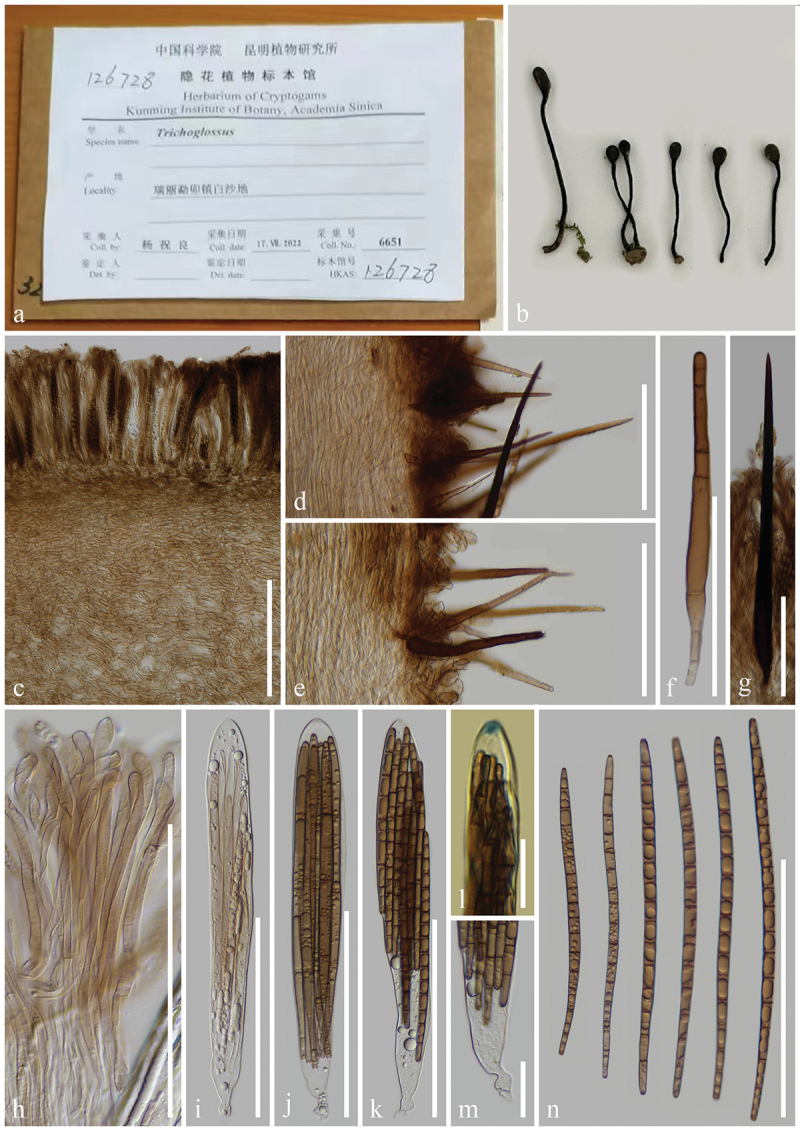
*Trichoglossum ruiliense* (HKAS 126728, holotype). (a, b) Herbarium specimen. (c) Longitudinal section of an ascigerous portion. (d, e) Longitudinal section of a stipe with setae. (f) Seta of hymenium with obtuse apex. (g) Seta of hymenium with sharp apex. (h) Paraphyses. (i–k) Asci. (l) Ascus apex in MLZ. (m) Ascus base. (n) Ascospores. Scale bars: c = 200 µm, d–k, n = 100 µm, l, m = 20 µm.

***Trichoglossum ruiliense*** H.L. Su, K.D. Hyde, Zhu L. Yang & Q. Zhao, sp. nov., Figure 14

*Index Fungorum number*: IF 903125; *Facesoffungi number*: FoF 17003.

*Etymology*: Referring to the type locality Ruili, Yunnan, China.

*Holotype*: HKAS 126728.

*Diagnosis*: *Trichoglossum ruiliense* is similar to *T. hirsutum* in microscopic characteristics, but differs from the latter in the size of ascomata.

*Description*: *Saprobic* on soil among moss. Sexual morph: *Ascomata* scattered, gregarious, clavate, stipitate, 1–2.5 cm in height when dried, hirsute, with numerous setae. *Ascigerous portion* slightly swollen, cylindrical, flattened lanceolate, with obtuse apex, 2–4 mm in height, 1–2.5 mm in diam. when dried, dark, densely hirsute from setae. *Stipe* 8–20 mm in height, 0.3–1 mm in diam. when dried, slender, straight to slightly curved, dark, concolorous with ascigerous portion, densely hirsute from setae. *Hymenium* 185–210 µm ($\overset{ˉ}{x}$ = 200 µm, *n* = 32) thick (without setae), consisting of setae, paraphyses, and asci at different stages of development. *Ascigerous portion interior* brown, composed of thin-walled hyphae of *textura intricate*, 3.5–8 µm ($\overset{ˉ}{x}$ = 5.5 µm, *n* = 302) in diam. *Stipe surface* 15–40 µm ($\overset{ˉ}{x}$ = 32 µm, *n* = 20) thick (without setae), composed of irregularly arranged, short, light brown, swollen cells, 8–19.8 × 7–9.3 µm ($\overset{ˉ}{x}$ = 15 × 7 µm, *n* = 33). *Stipe interior* cells of *textura porrecta*, light brown, composed of thin-walled hyphae, 1.5–7.5 µm ($\overset{ˉ}{x}$ = 3.9 µm, *n* = 104) in diam. *Setae* (90–)95–365(−400) × (6–)7.5–17.5(−20) µm ($\overset{ˉ}{x}$ = 220 × 12.5 µm, *n* = 66), heavily protruding above paraphyses and asci, acicular, most with sharp apex, sometimes with obtuse apex, multi-septate, brown to dark brown, thick-walled. *Paraphyses* filiform to cylindrical, protruding above the asci, with few septa, most one, or no septum in the middle, pale brown, lower portion 2.5–7 µm ($\overset{ˉ}{x}$ = 4.4 µm, *n* = 55) in diam., apical portion slightly curved, slightly swollen, 3.5–7.5 µm ($\overset{ˉ}{x}$ = 5.3 µm, *n* = 56) in diam. *Asci* (185–)190–220(−230) × (15–)16.5–26(−28) µm ($\overset{ˉ}{x}$ = 205 × 21 µm, *n* = 60), 8-spored, cylindrical-clavate, attenuated towards the base, apically thick-walled, laterally thin-walled, with tapered, obtuse, amyloid apex, and have croziers. *Ascospores* (165/4/1) (115–)133–166(−179) × (3.6–)4–6.2(−7) µm ($\overset{ˉ}{x}$ = 152 × 5 µm, Q = 19.9–41.2, ***Q*** = 30.54 ± 4.29), fasciculate, mostly filiform to acicular, obtuse apex, relatively acuminate base, straight to slightly curved, thick-walled, mostly 16-guttulate and 15-septate, dark brown. Asexual morph: Not observed.

*Material examined*: China, Yunnan Province, Ruili, Mengmao, on soil among moss, 17 July 2022, Z.L. Yang, Yang 6651 (HKAS 126728, **holotype**).

*Notes*: In the phylogenetic analyses, *Trichoglossum ruiliense* (HKAS 126728) forms an isolated clade within *Trichoglossum*-II with 69% bootstrap and 0.94 Bayesian posterior probability. The species is characterised by small, clavate ascomata with slightly swollen, cylindrical to flattened lanceolate ascigerous portion, filiform, 0–1-septate paraphyses with a slightly swollen, slightly curved apex, relatively large asci (185–230 × 15–28 µm), and relatively large, mostly 15-septate, dark brown ascospores (115–179 × 3.6–7 µm). *Trichoglossum ruiliense* is similar to *T. variabile* in macromorphology, the size of asci, the size, and the septum of ascospores. However, *Trichoglossum ruiliense* differs from *T. variabile* in paraphyses, the first has 0–1-septate paraphyses with a slightly swollen, slightly curved apex, while the last has septate paraphyses with an enlarged, bent, or convolute apex. Based on these morphological and phylogenetic analyses, *T. ruiliense* is introduced as a new species.

**Figure 15.**
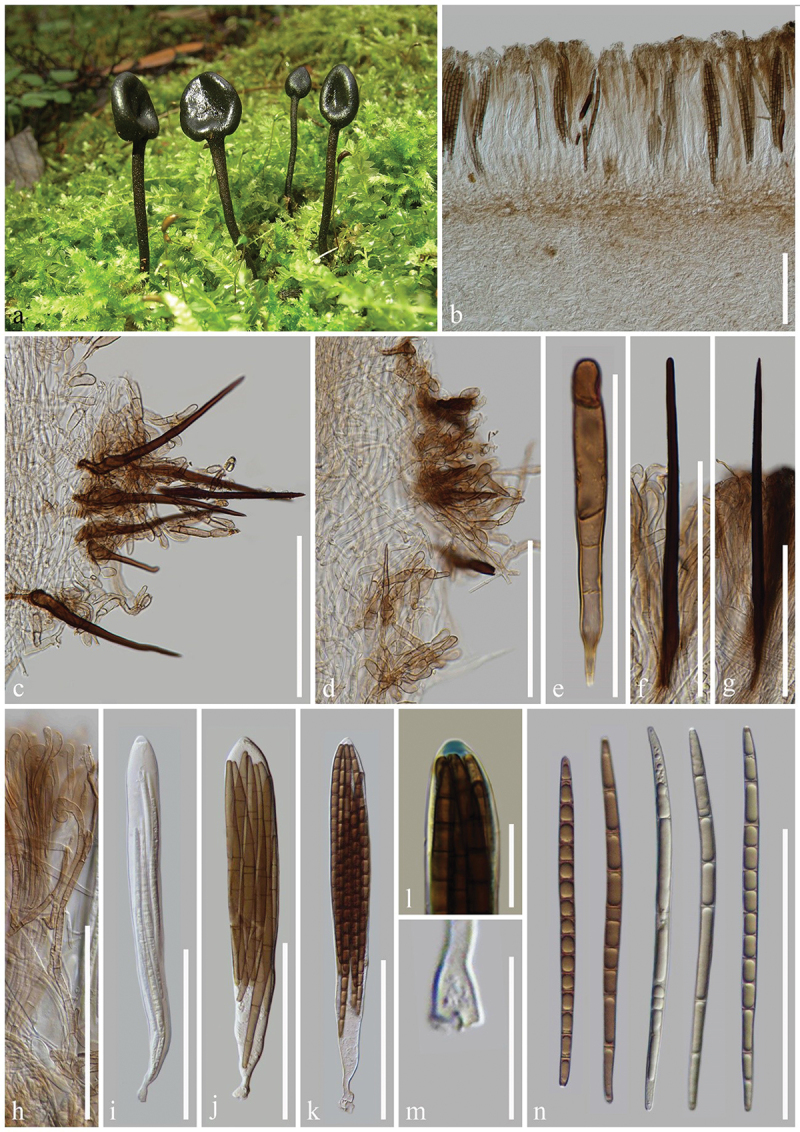
*Trichoglossum subhirsutum* (HKAS 112832, holotype). (a) Fresh ascomata. (b) Longitudinal section of an ascigerous portion. (c, d) Longitudinal section of a stipe. (e–g) Setae of hymenium. (h) Paraphyses. (i–k) Asci. (l) Ascus apex in MLZ. (m) Ascus base. (n) Ascospores. Scale bars: b–k, n = 100 µm, l, m = 20 µm.

***Trichoglossum subhirsutum*** H.L. Su, K.D. Hyde, Zhu L. Yang & Q. Zhao, sp. nov., Figure 15

*Index Fungorum number*: IF 903126; *Facesoffungi number*: FoF 17004.

*Holotype*: HKAS 112832.

*Diagnosis*: *Trichoglossum subhirsutum* is morphologically and ecologically similar to *T. hirsutum*, but differs from the latter by its wider ascigerous portion and septate setae.

*Etymology*: Referring to the morphological similarity with *Trichoglossum hirsutum*.

*Description*: *Saprobic* on soil among moss. Sexual morph: *Ascomata* scattered, gregarious, sometimes caespitose, capitate, stipitate, 2.5–9 cm in height, hirsute, with numerous setae. *Ascigerous portion* swollen, flattened lanceolate, or irregularly folded, or triangular with severe central depressions, more or less compressed, with obtuse apex, 5–10 mm in height, 5–10 mm in diam., black, concolourous with the stipe, densely hirsute from setae. *Stipe* 20–60 mm in height, 1–3 mm in diam., cylindrical, straight to slightly curved, densely hirsute from setae. *Hymenium* 205–285 µm ($\overset{ˉ}{x}$ = 237 µm, *n* = 19) thick (without setae), consisting of setae, paraphyses, and asci at different stages of development. *Ascigerous portion interior* light brown to hyaline, composed of septate, thin-walled hyphae of *textura porrecta*, 2.2–5.3 µm ($\overset{ˉ}{x}$ = 3.5 µm, *n* = 53) in diam. *Stipe surface* 35–95 µm ($\overset{ˉ}{x}$ = 60 µm, *n* = 28) thick (without setae), composed of irregularly arranged, long, septate, light brown hyphae, 4–9.2 µm ($\overset{ˉ}{x}$ = 6.1 µm, *n* = 41) in diam. *Stipe interior* hyphae of *textura porrecta*, brown, composed of thick-walled hyphae, 2–6.6 µm ($\overset{ˉ}{x}$ = 4 µm, *n* = 128) in diam. *Setae* 70–260 × 5.7–12.3 µm ($\overset{ˉ}{x}$ = 154 × 7.9 µm, *n* = 12), heavily protruding above paraphyses and asci, acicular, with sharp apex, sometimes with obtuse apex, septate, brown to dark brown, or black, thick-walled. *Paraphyses* filiform, slight protruding above the asci, multi-septate, light brown to brown, lower portion 1.3–3.3 µm ($\overset{ˉ}{x}$ = 2.1 µm, *n* = 98) in diam., apical portion curved, swollen, 3–8 µm ($\overset{ˉ}{x}$ = 5.2 µm, *n* = 70). *Asci* (195–)199–230(−232) × (16–)17–27(−30) µm ($\overset{ˉ}{x}$ = 213 × 21 µm, *n* = 45), 8-spored, cylindrical-clavate, attenuated towards the base, apically thick-walled, laterally thin-walled, with tapered, obtuse, amyloid apex, and croziers. *Ascospores* (134/4/1) (100–)115–154(−164) × (4–)4.5–6.3(−7.6) µm ($\overset{ˉ}{x}$ = 137 × 5.4 µm, Q = 13.9–35.7, ***Q*** = 25.5 ± 3.53), fasciculate, filiform, obtuse apex, straight to slightly curved, thin-walled, 7–16-guttulate, mostly 15-septate, rarely 2–10-septate, light brown to dark brown. Asexual morph: Not observed.

*Material examined*: China, Yunnan Province, Kunming, the Kunming Institute of Botany, on soil among moss in broad-leaf forests, 22 September 2020, Z.L. Yang, Yang 6436 (HKAS 112832, **holotype**); *ibid*., 20 September 2020, H.L. Su, SHL13B (HKAS 137597, **paratype**); *ibid*., 22 September 2020, Z.L. Yang, Yang 6454 (HKAS 112850, **paratype**); *ibid*., 29 September 2020, T. Guo, GT50 (HKAS 69042, **paratype**).

*Notes*: In our phylogenetic analyses, *Trichoglossum subhirsutum* (HKAS 137597, HKAS 69042, HKAS 112832, HKAS 112850, and HKAS 112852) forms a strong and isolated clade with 97% bootstrap and 1.00 Bayesian posterior probability within *Trichoglossum*-II. *Trichoglossum subhirsutum* closely resembles *T. hirsutum* in most morphological features. In addition, *T. subhirsutum* and *T. hirsutum* share a similar habitat and live on soil in broadleaf forests (Persoon 1794; Boudier 1907). However, the two species can be distinguished by their wider ascigerous portion (5–10 mm vs. 2–5 mm) and septate setae (Imai 1941; Kaygusuz et al. 2020). In addition, *T. subhirsutum* (HKAS 112852) and *T. hirsutum* (ILLS 67355, ILLS 61045, Jamie Platt JP267, OSC 61726, HKAS 55133, and HKAS 22) have distant phylogenetic relationships (Figure 1) and significantly different sequences (Table 5) (Jeewon and Hyde 2016). Based on these morphological, phylogenetic, and ecological analyses, *T. subhirsutum* is introduced as a new species following the guidelines of Chethana et al. (2021).

**Table 6.** Comparison of *Geoglossum* species and detailed descriptions.

Species	Ascomata	Ascigerous portion	Stipe	Paraphyses	Asci	Ascospores	Reference
*Geoglossum ailaoense*	Linear to cylindrical, 2.5–4.5 cm high (when dry), dark brown to black, without setae	Cylindrical, sometimes compressed, obtuse, 15–35 × 0.5–1 mm (when dry)	Cylindrical, slender, sometimes slightly compressed, 10–20 × 0.5–1 mm (when dry)	Filiform to cylindrical, multi-septate, apical portion slightly fastigiate, slightly swollen to drop-shaped or globose, with swollen internode, protruding above the asci, lower portion 1.5–4.5 µm ($\overset{ˉ}{x}$ = 2.8 µm, *n* = 101) in diam., apical portion 8–24.5 × 5–9 µm, $\overset{ˉ}{x}$ = 13.5 × 6.6 µm, pale brown	Cylindrical-clavate, with croziers, (180–)185–210(−215) × 15–23(−25) µm, $\overset{ˉ}{x}$ = 198 × 19 µm, 8-spored, amyloid	Clavate-filiform to acicular, mostly 7-septate, (78–)81–98(−106) × (3.3–)3.9–5.5(−6.4) µm, $\overset{ˉ}{x}$ = 89 × 4.7 µm, Q = 13.9–26.4, ***Q*** = 19.18 ± 2.55 brown	This study
*Ge. alpinum*	Clavate, somewhat compressed, 1.4–1.8 cm high, black, without setae	-	Glabrous or faintly verruculose	Filiform, straight, multi-septate, apical portion slightly swollen, 1–3 µm thick, uppermost cell 5–7 µm thick, hyaline below, coloured above, strongly agglutinated by a dark brown, amorphous matter	Clavate, 130–150 × 18–22 µm, 8-spored	Clavate, 7-septated, 55–70 × 5.5–7.5 µm, fuligineous brown	Eckblad (1963)
*Ge. australe*	2–4 cm high, dark brown to black, without setae	Slightly compressed, 1/3 to 1/2 the total length of the ascoma, up to 2 mm wide	Cylindric, slightly compressed, up to 2 mm thick, dark brown, distinctly alveolate	Cylindrical, often curved above, slightly agglutinated at the apex, septate, with swollen apex, constricted at the septa, longer than the asci, apex up to 8 µm in diam., light brown	Cylindric-clavate, (160–)180–200(−215) × (14.5–)17–20(−22) µm, 8-spored, amyloid	Clavate-subfusoid, 3- or 7-septate, (81–)90–105(−115) × 5.5–6.5(−7.5) µm, initially hyaline, light brown at maturity	Hustad (2015)
*Ge. azoricum*	Gracile, furrowed, 44–70 mm high, blackish, without setae	Cylindrical, clavate to lanceolate, subcylindrical to spatulate in cross section, compressed, with one or more longitudinal furrows, 19–20 × 4.2–4.8 mm, 1.7–2.2 mm thick, blackish, brownish in the area near the stipe, smooth or somewhat wrinkled	Terete, usually recurved, 24–50 × 1–1.8 mm, greyish blackish or brownish, paler brownish at apex, flesh greyish, scaly	Filiform, septate, with capitate, pyriform, obovoid, claviform, straight to hooked apex, 5–6.5 µm wide in middle, apex 15–30 × 5–11(−17) µm, hyaline base, with greenish-greyish or brownish-full parietal and encrusting pigment	Clavate, fusiform, with rounded apex, narrowed below, and croziers, 180–220(−250) × 15–19(−25) μm, 8-spored, hemiamyloid	Cylindrical-clavate, sub-fusiform, with acute basal end, 3–7-septate, mostly 7-septate, (65–) 72.7–93.7(−99.4) × (4.7–)5–6.1(−6.3) μm, $\overset{ˉ}{x}$ = 84.1 × 5.4 µm, Q = (13.1–)13.7–17.5(−18.6), ***Q*** = 15.1, initially hyaline, aseptate, finally dark brown	Crous et al. (2023)
*Ge. barlae*	Linguiform or slenderly lanceolate, with groove that sometimes divides the clavula into two halves beneath the tip, up to 3.5 cm in height, and 0.8 cm in width, black, without setae	-	-	Crozier-like, multi-septate, duck-head shape, twisted, hyaline to slightly brownish	Clavate cylindrical, with somewhat fusiform, rounded, amyloid apex, 215–245 × 17–25 μm, 8-spored	Cylindrical to fusiform, with one tapering end, 7-septate 53–69 × 5–6 μm, hyaline when young, sooty at maturity	Aouali (2021)
*Ge. berteroi*	Cylindric, slender, erect, flexuous, 15–29 mm high, without setae	Spathulate, 6–15 × 0.5–1.5 mm, chestnut brown to purplish brown when fresh, black-brown when dry, giving a purplish brown colour with KOH solution	Cylindric, 8–25 × 0.5–1.5 mm, dark chestnut brown when fresh, black when dry, pubescent	Clavate, branched, longer than the asci, 0.8–1.3 µm thick at the apex, containing a purplish brown pigment	Cylindric-clavate, 50–72 × 2.4–4 µm, 8-spored, amyloid	Cylindric, aseptate, (4.5–)5.8–10 × 0.8–1.4 µm, hyaline	Zhuang and Wang (1997)
*Ge. brunneipes*	Nail or lance-shaped, irregularly thickened when mature, 30–79 mm high, without setae	Elliptic to lance-shaped, irregularly thickened, compressed on one side, with one or several longitudinal grooves that give it a wrinkled appearance when mature, (8–)18–33(−42) × (3–)5–8(−9.7) mm, black	Subcylindrical to laterally compressed with a longitudinal groove, straight or twisted, 19–57 × 1.4–4.8 mm, brown to dark reddish-brown, with a darker base	Filamentous, slightly thickened at the tips, initially formed by cylindrical elements, the terminal element thickens moderately to strongly at maturity, becoming clavate, capitate, or pyriform, occasionally fusoid or lanceolate, straight or somewhat curved, (15–) 25–40(−54) × (5–) 9–12 (−15) µm, $\overset{ˉ}{x}$ = 34.2 × 10.1 µm, overall grey or slightly lighter greyish at the base, sometimes darker brown, occasionally more brownish at the apex, paraphyses often appear slightly to moderately cohesive in rehydrated material	Nearly cylindrical, slightly fusoid with a short stalk and a rounded apex, 164–190 × 17.2–21.8 µm, 8-spored, amyloid	Cylindrical-clavate, straight or slightly curved, 7-septate, (60.2–)66.6–68.7(−75.1) × (4.5–)5.3–5.6(−6.5) µm, $\overset{ˉ}{x}$ = 67.7 × 5.5 µm, Q = (10.1–)12.1–12.7 (−14.7), ***Q*** = 12.4, dark brown at maturity	Arauzo and Iglesias (2014)
*Ge. chamaecyparinum*	Club-shaped, or slender, 27–62 mm high, blackish to dark brown, without setae	Cylindrical and sometimes elliptical or club-shaped, sometimes swollen or lanceolate, 8.9–18.8(−26) × 2.2–4.8 (−7.5) mm, with one or two indistinct longitudinal ridges, blackish-brown	Cylindrical, occasionally broader at the top, sometimes compressed or longitudinally grooved, 19.4–36(−48.5) × 1–2(−3.7) mm, slightly lighter brown	Filiform, with a thickened, capitate apex, curved, sometimes with nodules or irregular shapes, (19–)42–72.2(−91.3) × (3.4–)6–8(−9.6) μm, $\overset{ˉ}{x}$ = 55.59 × 6.46 μm, ***Q*** = 9.06, with light fuliginous grey pigment at the apex	Cylindrical to slightly fusiform, with rounded apex or slightly irregular, (164.4–)181.4–195.8(−217) × (16–)19.4–23.2(−25.7) μm, $\overset{ˉ}{x}$ = 189.4 × 21.2 μm, Q = (7.2) 8.6–9.5 (11), ***Q*** = 9.05, 8-spored, amyloid	Clavate, 7-septate, (49.5–)72.1–82.2(−89.6) × (5.2–)5.8–6.1(−6.7) μm, $\overset{ˉ}{x}$ = 78.4 × 5.94 μm, Q = (10.7–)12.8–13.6(−15.7), ***Q*** = 13.22	Arauzo and Iglesias (2014)
*Ge. cookeanum*	Clavate, 1–6 cm high, brownish black to black	Lanceolate, terete, compressed, with tapering apex, 10–54 × 5–15 mm	Cylindrical, (9–)18–33(−41) × 2–6 mm	Cylindrical, slender, 2.3–4.5 µm thick, septate, the apical cells barrel-shaped to globose, often in chains of alternating thicker and slender cells, 5–10 μm thick, straight to somewhat curved, pale brown in upper part	Cylindrical-clavate with narrowed obtuse apex, 155–210 × 13–24 µm, 8-spored, amyloid	Cylindrical, tapering towards one end, 7-septate, 55–83 × 5–7 µm, dark fuliginous	Kučera and Lizoň (2012); this study
*Ge. dunense*	Club-shaped, 1–2.3 × 0.8–1.1 cm, black, without setae, without setae	Enlarged, club-shaped, sometimes cerebriform or rarely tongue-shaped, irregularly lobed, 0.5–1.2 × 0.5–1.1 cm	0.5–1.1 × 0.1–0.2 cm, minutely squamulose	Irregularly polymorphic, mostly moniliform with irregular clavate thickenings, multi-septate, constricted at the septa, sometimes branching, often flexuous or contorted, with enlarged, clavate, subcapitate, sometimes forked or hooked tips, exceeding the length of the asci, up to 7 μm wide, grey	Clavate, 166–205 × 17–27 μm, 8-spored, amyloid	Fusiform to subfusiform or rarely subcylindrical, slightly bent, often somewhat rounded at one end, 0–4-septate, (28–)31–44(−53) × (7–)8–10(−12) μm, Q = (3.5–) 4.3–5.7 (−6.4), ***Q*** = 5, dark grey to greyish-brown, thin-walled, multi-guttulate at maturity	Loizides et al. (2015)
*Ge. fallax*	Clavate, 2–8.5 cm high, fulvous or brownish, blackish when dried, without setae	Clavate, lanceolate or elongated ellipsoid, obtuse, compressed, 8–15 × 3–5 mm	Slender, terete, upwards slightly thickened, downwards 1–2 mm thick, squamulose	Filiform and hyaline below, with curved or circinate, with abruptly ellipsoid or globose tips, becoming subhyaline or pale fuliginous above	Cylindrical-clavate, with contracted apex, 150–250 × 17.5–20 μm, 8-spored, amyloid	Cylindrical-clavate, straight or curved, 62–105 × 5–7 μm, initially hyaline, later becoming dark brown, 7–12-septate	Imai (1941)
*Ge. geesterani*	Cylindrical and tapered, up to 35–55 mm high, dark blackish, without setae	Cylindrical and somewhat ellipsoidal or occasionally club-shaped from the edge to a somewhat compressed centre, with one side having longitudinal groove, sometimes wrinkled lengthwise, 14–28 × 2.3–4.3 mm	Cylindrical or slightly compressed lengthwise, sometimes furrowed, 20–35 × 1.2–2.7 mm, dark brown to deep reddish-brown	Filiform, with appendages or branches, slightly longer than the asci, 1.2 to 2.5 µm wide, base hyaline, the apex broadly claviform or pyriform, curved or circinate, (14) 21–24 (31) × (5) 7–9 (12.5) µm, $\overset{ˉ}{x}$ = 22.5 × 8 µm, light grey smoky	Spindle-like, with a slightly ridged or somewhat ribbed edge, (150–)170–187(−205) × (14–)17–19.5(−22.5) µm, $\overset{ˉ}{x}$ = 178.8 × 18.3 µm, Q = (7.7–)9.2–10.5(−12.1), ***Q*** = 9.9, 8-spored, amyloid	Cylindrical-clavate, 7-septate, (50–)58.5–61.6(−71.5) × 5.2–6.9 µm, $\overset{ˉ}{x}$ = 60.04 × 6.04 µm, Q = (8–)9.7–10.2(−11.9), ***Q*** = 9.97, initially hyaline and ultimately colored	Arauzo and Iglesias (2014)
*Ge. glabrum*	Setae absent	Elongate-lanceolate to elongate-clavate, 2–34 × 1–3 mm, dark brown to black	6–47 × 0.4–1.5 mm	Apex enlarged and moniliform, 2–4 cells, straight or curved	152–203 × 18–23 µm, 8-spored, amyloid	Clavate, narrower at the lower end, 7-septate, (51–)59–91(−96) × 5–7 µm	Zhuang and Wang (1997)
*Ge. inflatum*	Up to 6 cm high, black, without setae	1/2 of the ascoma	Terete, up to 4 mm wide at the apex	Cylindrical, septate, slightly constricted at the septa, distinctly inflated to 8 µm in diam. at the apex, hyaline below, light brown above	Clavate, (150–)160–174(−200) × (15–)17.5–19(−22) µm, 8-spored, amyloid	Cylindric-clavate, 7-septate, (65–)70–81(−88) × (4.5–)5.5–7(−7.9) µm, brown	Hustad (2015)
*Ge. jirinae*	Clavate, 18–36 × 2–4 mm (when dry), black, without setae	Lanceolate or broadly clavate, compressed, 1/3–2/3 of the total ascomata length	Cylindrical, slightly thickened upward, 9–15 × 1.5–2.5 mm, squamulose	Straight, septate, 2–3 µm wide, hyaline, agglutinated by light brown amorphous matter in apical part, apical cells variable, cylindrical, clavate to capitate, curved, contorted, sometimes bifurcate, or proliferating, mostly 22–47 × 2–3 µm, some cells inflated up to 10 µm	Cylindrical to clavate, (123–)135–145(−166) × 14–17 µm (in 3% KOH), ***Q*** = 7.8–9.7	Elongated clavate to ellipsoid baculiform, 1–4(−7)-septate, mostly 3-septate, (39–)45–57(−60) × 5–6(−6.5) µm, ***Q*** = 7–10(−12), first hyaline, finally brown	Crous et al. (2020a)
*Ge. laccatum*	50–57 mm high when dry, gelatinous, black, without setae	Lanceolate to clavate, compressed, 14–15 × 2.5–3 mm	Nearly terete, 20–30 × 1.5–2.5 mm, outer layer obviously gelatinous	Filiform, septate, with unswollen and strongly curved or circinate apex, 2.5 µm in diam. below, subhyaline	Clavate, 228–254 × 18–20 µm, 8-spored, amyloid	Subcylindrical, slightly narrow at the lower end, 7-septate, 72–94 × 6–7 µm	Zhuang and Wang (1997)
*Ge. laurisilvae*	Gracile, cylindrical or subcapitate but usually clavate, 14–48 mm high, dull grey blackish, dark brown or black, without setae	Cylindrical to clavate, spatulate, sometimes longitudinally furrowed, 5–17 × 1.5–4 mm, felted in appearance	Terete, sometimes compressed, lobed with longitudinal furrow, generally recurved, occasionally serpentiform, narrowed towards the base, which ends somewhat widened, 9–37 × 1.1–2.2 mm, greyish brown to dark, upper part finely fibrillose, with small scales, lower part more cylindrical, smooth, sometimes granulate	Filiform, constricted at the septa, 2.5–3.5 mm wide in middle, with distinctly and variously enlarged, broadly clavate, capitate, pyriform or obovoid, sometimes with appendage or somewhat curved or flexuous, hook-like or circinate apex, apex (14–)17–37 × (5–)5.5–8(−11) µm, hyaline base, apex with parietal or encrusting grey-fuliginous pigment	Clavate, cylindrical or fusiform, with rounded apex, narrowed below, and croziers, (160–)175.5–190(−205) × (14.4–)14.7–16(−19) µm, $\overset{ˉ}{x}$ = 182.7 × 15.6 µm, 8-spored, amyloid	Cylindrical-clavate, subfusiform, acute basal ends, rarely 1- or 3-septate, 7-septate, (65–)71.6–86(−90) × (4.8–)5–6.47(−6.5) µm, $\overset{ˉ}{x}$ = 79.3 × 5.8 µm, initially hyaline and aseptate, finally dark brownish full-brown	Crous et al. (2022)
*Ge. lignicola*	4–5 cm high, black, with setae	Clavate, round or compressed, about 3 mm thick	Slender, usually crooked, velvety	Clavate, straight, septate, about 6 µm thick, apex tinged olive	Clavate, obtuse, 150 × 15 µm, 8-spored, amyloid	Linear-clavate, 7-septate, brown, translucent	Massee (1896)
*Ge. lijiangense*	Clavate, tongue-shaped, 5–7.5 cm high, brownish black to black, without setae	Terete to lanceolate, more or less compressed, with obviously shallow and longitudinal depressions in the centre, tapering apex, 20–25 × 2.5–4 mm	Cylindrical or slightly compressed, 25–60 × 1–2 mm, with tiny squamules	Filiform, protruding above the asci, septate, pale brown overall, lower portion 2.4–4.5 µm, $\overset{ˉ}{x}$ = 3.4 µm in diam., apical portion swollen to globose, 9.5–17 × 6–9.5 µm, $\overset{ˉ}{x}$ = 12.8 × 8 µm, evenly thin constricted at the septa	Cylindrical-clavate, with croziers, 210–245(−250) × (15–)18–28(−30) µm, $\overset{ˉ}{x}$ = 225 × 22 µm, 8-spored, amyloid	Clavate-filiform to acicular, 7-septate, with round apex and relatively acuminate base, (73–)81–103(−116) × (4.5–)5–7.5(−8.5) µm, $\overset{ˉ}{x}$ = 91.2 × 5.4 µm, Q = 10–26, ***Q*** = 15.69 ± 2.55, dark, brown	This study
*Ge. lineare*	Narrowly linear, terete or slightly compressed, up to 4.5 cm high, black, without setae	Elongate, 1/3–1/2 of the total ascomata length, up to 4 mm in diam.	Smooth, viscid	Filiform, with gradually incrassate, ellipsoidal-globular apex, straight, septate, distinctly longer than the asci, 4.5–5.5 µm in diam. below, 12.5–14 µm in diam. above, basally hyaline, above subhyaline to pale brown	Clavate, obtuse, 130–160 × 12–16.5 µm, 8-spored, amyloid	Cylindrical-clavate, 3–7-septate, 41–70 × 4.5–6 µm, pale brown	Hakelier (1967); Hustad (2015)
*Ge. montanum*	Up to 3 cm high, black, without setae	Narrow, slightly compressed, 1/4–1/3 of the total ascomata length, up to 2 mm wide at widest point	Slender, up to 2 mm wide at the apex	Filiform, slender, with curved or coiled, swollen tip cells, brown, apex up to 5 µm wide	120–163 × 14–20 μm, 4–6-spored, amyloid	Clavate, 7-septate, 42.5–70 × 5–7 μm, light brown	Ohenoja (1995); Hustad (2015)
*Ge. muelleri*	Clavate, 1.5–2 cm high, black, without setae	Clavate, compressed, 1/3 to 1/2 the total length of the ascoma, 2–3 mm wide at widest point	1–2 mm thick at apex, conspicuously squamulose	Filiform, with swollen and strongly agglutinated above, septate, 2–3 µm in diam. in middle, 4–7 µm in diam. at the apex, dark brown	Clavate, (128–)135–155(−160) × (15–)16–18(−19) µm, 8-spored, amyloid	Cylindric-clavate, 3–7-septate, (49.5–)53–67(−71) × (5.5–)6–7 µm, light to dark brown	Hustad (2015)
*Ge. paludosum*	0.5–2 cm high, brownish black whe dried, with setae	Narrow, compressed, 1/2 the total length	Terete, very slender, minutely hairy	Cylindrical, straight, the terminal 2–3 cells clavate-thickened, the apical one usually more swollen and piriform to elliptical in outline, slightly constricted at the septa, 3 µm thick in middle, apex 10–14 × 7–8 µm, pale, the apex brown	Clavate, narrowed apex, 175–200 × 17–18 µm, 8-spored	Cylindrical-clavate, tapering slightly each way from above the middle, 15-septate, 122–140 × 6–7 µm, brown or fuliginous	Durand (1908a)
*Ge. peckianum*	Clavate, compressed, 3–10(−15) cm high, black, viscid when fresh, without setae	Strongly compressed, occupying the upper 1/4 to 1/2 of the ascoma, 5–20 mm wide at the widest point, black	Terete, up to 6 mm wide at the apex, compressed	Slender, coiled at the apex, sparsely septate, slightly longer than the asci, hyaline to subhyaline below, slightly brownish at the apex	Clavate, 240–275 × 18–25 µm, 8-spored, amyloid	Cylindrical-clavate, broadest at the centre, 15-septate, (70–)95–120(−135) × 6–7 µm, brown	Hustad (2015)
*Ge. pseudoumbratile*	Lanceolate, 3–8 cm high, black, without setae	1/3 to 1/2 of the ascoma, up to 6 mm wide at widest point	Cylindric	Cylindrical, straight, with inflated, variously curved to hooked apices, the apices up to 6 µm in diam., hyaline below, lightly pigmented above	Cylindric-clavate, (170–)181–223(−231) × (17–)19–22(−24.5) µm, 8-spored, amyloid	Cylindric-clavate, 7-septate, (61.5–)80–95.5(−107) × (5–)5.5–6.5(−7) µm, light to dark brown	Hustad (2015)
*Ge. pumilum*	8–12 mm high when dry, black, without setae	Spathulate, subcapitate to shortly clavate, 2–3.5 × 1 mm	6–10 × 0.3 mm, squamulose	Slightly enlarged at the apex, light brown	160–194 × 18–23 µm, 8-spored	Cylindrical-clavate, narrow at the lower end, mostly 15-septate, rarely 12–14-septate, 79–132 × 5–7 µm, brown	Zhuang and Wang (1997)
*Ge. pygmaeum*	Capitate, 35–50 mm high, without setae	Slightly globoid to ellipsoid, slightly undulate to somewhat sharp margin, sometimes compressed with a longitudinal groove, 1/4 to 1/5 of the apothecium, 8.5–13 × 4.4–7.6 mm wide, black	Cylindrical or slightly broader at the apex, straight or somewhat twisted, 30–40 × 1.6–2.9 mm, dark brown to blackish, hiary	Filiform, apex thickened, slightly to moderately moniliform, apex cell (9.2–)14.4–18.5(−29.3) × (7.1–)9.1–11.7(−15.9) µm, $\overset{ˉ}{x}$ = 16.46 × 9.87 µm, Q = (1–) 1.5–1.8(−2.3), ***Q*** = 1.65	Cylindrical to ellipsoid-shaped, with a slightly rounded or somewhat sharp-edged obtuse, 215–245 × 17–25 µm, amyloid	Cylindrical to slightly curved, 15-septate, (105–)122–139.5(−149.6) × (5.3–)5.9–6.3(−6.9) µm, $\overset{ˉ}{x}$ = 126.8 × 6.1 µm, Q = (16.4–)20–21.9(−25.4), ***Q*** = 20.9, dark brown to fuliginous	Arauzo and Iglesias (2014)
*Ge. raitviirii*	Clavate, 2.2–4.5 cm high, dark brown, without setae	Clavate, broadly clavate, compressed, 1/4 to 1/2 of the total ascocarp length, 0.5–1.8 cm long, black to dark brown, darker than the stipe, hairy	Terete, compressed, brown to dark brown, squamous	Straight, sometimes branched, with swollen, pyriform, globose, hockey stick-like, hook-like, cylindrical tips, septate, 2–5 µm, $\overset{ˉ}{x}$ = 3 µm in diam. in middle, apical cells (12.5–)32.5–38.5(−97) × (4.5–)8.5–9.5(−13.5) µm, pale brown, incrusted	Clavate to broadly clavate, (161.5–)172.5–176.5(−191.5) × (21–)24.5–26.5(−31) µm, 8-spored, amyloid	Elongate-clavate, subfusiform to fusiform, narrowed to basal end, 3–11-septate, mostly 7-septate, (49–)76.5–81.5(−93.5) × (5.5–)6–6.5(−9.5) µm, brown, sometimes hyaline, with pigmented septae and poles	Crous et al. (2016)
*Ge. scabripes*	Cylindrical, apically capitate, slightly curved, 12–13 mm high, dark chestnut to blackish brown, without setae	Cylindrical, slightly curved, 3–7 × 1.7–3.2 mm, not compressed or only slightly so on one side, lacking longitudinal ridges	Cylindrical or slightly compressed, 8–16.5 × 0.8–1.5 mm, slightly lighter than ascigerous portion	Filamentous, with distinct branches at the lower part, sometimes slightly swollen, straight or curved apex cells, (12.6–)18.2–26.4(−35.4) × (4–)5.2–7.3(−8.5) µm, $\overset{ˉ}{x}$ = 20.97 × 5.77 µm, Q = (1.6–) 3.2–4.3 (5.9), ***Q*** = 3.78	Cylindrical, obtuse, (146.2–)155.3–190.4 (−205.4) × (12.1–)15.5–19(−21) µm, $\overset{ˉ}{x}$ = 181.3 × 16.4 µm, Q = (8.7–)10.5–11.8(−13.6), ***Q*** = 11.2, 8-spored, amyloid	Cylindrical to ellipsoidal, 7-septate, (55.5–)62.8–73.7(−79.1) × (4.6–)5.3–5.7(−6.5) µm, $\overset{ˉ}{x}$ = 68.7 × 5.5 µm, Q = (9–)11.8–13.3(−16), ***Q*** = 12.5, darkened	Arauzo and Iglesias (2014)
*Ge. simile*	8–11 mm high, without setae	Broadly clavate, 3 × 1.6–1.7 mm, blackish brown to black	Terete, 3–6 × 1 mm, squamulose	Filiform, slightly enlarged at the apex, sometimes constricted at septa	135–165 × 17–22 µm, 4–8-spored, inamyloid	Clavate, slightly narrower at the lower end, 7-septate, 58–100 × 5–7 µm	Zhuang and Wang (1997)
*Ge. spathulatum*	Spathulate, compressed, 10–15 × 2–3 mm, dark, pilose, with setae	-	Sub-compressed, 3–4 cm long, about 2 mm in diam., pilose	Filiform, with capitate apex, septate, brown	Cylindraceous to subclavate, 80–90 × 9–10 µm, 8-spored, amyloid	Cylindraceous to clavate, pointed at both ends, 65–70 × 5–6 µm, multi-septate, light brown	Massee (1908)
*Ge. starbaeckii*	Up to 5 cm high, dusky brown to black, without setae	Lanceolate, compressed, occupying the upper 1/3 to 2/5 of the ascoma, up to 5 mm wide at widest part	Slender, dark brown to black	Slender, narrow below, inflating slightly at the apex, up to 5 µm, brown, not agglutinated by amorphous material at the apex	Cylindrical to clavate, 180–210 × 10–18 µm, 8-spored	Cylindral-clavate, 7–9-septate, (50–)67–85(−100) × (4.5–)5–6(−6.5) µm, light brownish	Hustad (2015)
*Ge. tetrasporum*	Clavate, 5–7.5 cm high, brown to dark, hirsute, with setae	Swollen, cylindrical, compressed, mostly with obviously shallow and longitudinal depressions in the centre, and tapered, obtuse apex, 10–25 mm in height, 1.5–3 mm in diam.	Cylindrical, 40–65 × 1–1.5 mm	Filiform, multi-septate, with obviously swollen and curved apex, protruding above the asci, lower portion 1.8–5 µm, $\overset{ˉ}{x}$ = 3 µm, in diam., apical portion 4.3–9.5 µm, $\overset{ˉ}{x}$ = 6.3 µm in diam., pale brown	Cylindrical-clavate, attenuated towards the base, with croziers, 205–235(−240) × (13–)16–21(−24) µm, $\overset{ˉ}{x}$ = 216 × 19 µm, 4-spored, amyloid	Cylindrical, 1–7-septate, mostly 1-septate, with tapered, obtuse ends, (65–)72–85(−90) × (4.5–)5–7 (−7.5) µm, $\overset{ˉ}{x}$ = 79 × 5.9 µm, Q = 10.3–18, ***Q*** = 13.5 ± 1.34, brown	This study
*Ge. tubaraoense*	Clavate, sulcate, sometimes contorted, 1–2.5 cm long, dark, hairy, without setae	3–10 × 3–8 mm	Teret to compressed sulcate, 8–13 × 1–1.5 mm	Filiform, septate, with obtuse, swollen apex	Cylindrical to clavate, apically obtuse, base tapered curved, 120–140 × 16–21 µm, 8-spored	Filiform, both sides of the tapered obtuse, 7-septate, 70–110 × 4–7 µm, brown	Hennings (1900)
*Ge. umbratile*	Lanceolate to cylindric, up to 7 cm high, black, without setae	Compressed, occupying the upper 1/4 to 1/2 of the ascoma, up to 10 mm wide at widest point	Cylindric to terete, up to 5 mm wide at apex	Filiform, septate, slightly constricted at the septa, curved to slightly hooked at the apex, only slightly inflated at the apex, not agglutinated, slightly longer than the asci, hyaline below, subhyaline to light brown above	Cylindric-clavate, (170–)183–208.5(−225) × (17–)18.5–22(−25) µm, 8-spored, amyloid	Cylindric-clavate, (56–)74–91(−102) × (5.5–)6.5–7(−7.5) µm, brown	Hustad (2015)
*Ge. variabilisporum*	Cylindrical to slightly clavate, slender, up to 6.3–19 mm tall, without setae	2–6.2 × 1.4–2 mm, dark	Cylindrical or slightly clavate, 4.3–13 × 1–1.5 mm, lighter above, darker base	Filiform, apex swollen to slightly or prominently cylindrical, straight or occasionally curved, (26.9–)53.8–77(−93.8) × (3.7–)5.3–7.1(−8.5) μm	Cylindrical to slightly clavate, (124–)145.5–168.8(−187) × (15.3–)18.3–21.2(−24) μm, $\overset{ˉ}{x}$ = 157.11 × 19.79 μm, Q = (6.8–)7.6–8.3(−9.1), ***Q*** = 7.96, 8-spored, amyloid	Claviform to vermiform, with a sharply rounded base, 0–14-septate, mostly 7-septate, (54.7–)72.4–92.9(−105.4) × (4.7–)5.6–6.7(−7.9) μm, $\overset{ˉ}{x}$ = 83.54 × 6.25 μm, Q = (8.6–)12.6–14.6(18.6), ***Q*** = 13.56, dark brown	Arauzo and Iglesias (2014)
*Ge. vleugelianum*	Cylindrical or lanceolate, about 41 mm high, blackish olive-toned, without setae	Lanceolate-shaped, compressed, occupying about 1/2 to 1/3 of the total length, 8.7–17.5 × 2–4.2 mm, dark olive to almost blackish olive-toned	cylindrical or slightly compressed, wider towards the apex, 14.2–26.8 × 1.3–2.5 mm, slightly lighter in colour than the clavula, surface scaly, especially in the upper part, tending to be smooth in the lower part or with fine longitudinal striations	Filiform, apex slightly short, swollen to subglobose or pyriform to clavate, straight or less curved, (8.2–)14.6–18.1(−27) × (4.4–)6.2–7.3(−8.2) µm, $\overset{ˉ}{x}$ = 16.37 × 6.45 µm, Q = (1.2)2.3–3(4.6), ***Q*** = 2.61, hyaline or light grey smoky	Cylindrical or fusiform, (122–)135.7–151(−164.2) × (12–)13.5–16.7(−18.7) µm, $\overset{ˉ}{x}$ = 143.35 × 15.14 µm, Q = (5.6–)8.5–11.1(−13.9), ***Q*** = 9.77, 8-spored, amyloid	Cylindrical to subcylindrical, 7-septate, (38–)56.8–66.3(−77.1) × (4.6–)5.5–5.8(−6.7) µm, $\overset{ˉ}{x}$ = 64.37 × 5.67 µm, Q = (8.4–)11–11.9 (−14.5), ***Q*** = 11.44	Arauzo and Iglesias (2014)
*Ge. xylarioides*	Dark, without setae	Elongate-ovate, sub-clavate, compressed, obtuse, 2–5 × 0.2 mm, dark	Cylindrical, tapered, 3–5 × 0.3–0.5 mm	Filiform, septate, with curved apex, 5 µm in diam.	Clavate, with round and incrassate apex, 120 × 18 µm, 8-spored	Cylindrical, obtuse, 7-septate, 45–60 × 5 µm, dark, or olivaceous	Pazschke (1896)
*Ge. yuxiense*	Clavate, 1.2–2.5 cm high, dark, without setae	Cylindrical to clavate, more or less compressed, with rounded apex, 3–7 × 1–2 mm, thicker than stipe	Cylindrical, slightly compressed, 9–22 × 0.2–0.7 mm, with tiny squamules	Filiform to clavate, straight to slightly curved, protruding above the asci, septate, hyaline to pale brown, lower portion 1.5–3.2 µm, $\overset{ˉ}{x}$ = 2.2 µm in diam., apical portion obviously curvy and swollen, 4.1–8.6 µm $\overset{ˉ}{x}$ = 6.3 µm in diam.	Cylindrical-clavate, with croziers, 155–180(−185) × (13–)15–23(−27) µm, $\overset{ˉ}{x}$ = 165 × 19 µm, 8-spored, amyloid	Various, clavate to filiform, falciform, acicular, 3–7-septate, with tapering, round apex, swollen middle and relatively acuminate base, straight to slightly curved, (31–)44–87(−97) × (3.8–)4.6–7.7(−10) µm, $\overset{ˉ}{x}$ = 66.5 × 5.8 µm, Q = 3.3–23.1, ***Q*** = 11.78 ± 3.26, mostly dark brown	This study

## 4. Discussion

In recent years, many studies have focused on *Discomycetes* in Asia (Ekanayaka et al. 2017b, 2018, 2019; Hongsanan et al. 2020a, 2020b; Phutthacharoen et al. 2022; Zeng et al. 2022, 2023; Lestari et al. 2023; Lestari and Chethana 2024; Li et al. 2024; Luo et al. 2024). Among them, *Geoglossomycetes* is a class with a cosmopolitan distribution (Hustad et al. 2011, 2013, 2014; Fedosova and Kovalenko 2015; Hustad and Miller 2015a; Fedosova 2019; Kaygusuz et al. 2020; Aouali 2021; Crous et al. 2022; de la Fuente et al. 2022). Our phylogenetic analyses, supported by previous phylogenetic studies (Arauzo and Iglesias 2014; Hustad and Miller 2015a; Ekanayaka et al. 2017a; Fadnes et al. 2021; Kučera et al. 2021), show that *Geoglossaceae* is a monophyletic family comprising ten strongly supported clades (Figure 1). Notably, *Trichoglossum* is the only polyphyletic genus (Ekanayaka et al. 2017a; de la Fuente et al. 2022). However, the support among the nine genera is relatively low. Future research could enhance the understanding of their phylogenetic relationships through more in-depth studies or by employing newer methodologies.

*Geoglossum* accommodates 47 legitimate species, with sequences published for 19 of them, leaving 28 species without published sequence (Massee 1896; Pazschke 1896; Hennings 1900; Durand 1908a; Imai 1941; Eckblad 1963; Hakelier 1967; Gamundi 1977; Ohenoja 1995; Zhuang and Wang 1997; Kučera and Lizoň 2012; Arauzo and Iglesias 2014; Hustad 2015; Loizides et al. 2015; Crous et al. 2016, 2020a, 2020b, 2022, 2023; Aouali 2021). In addition, we did not examine the detailed morphological descriptions of eight *Geoglossum* species (*Ge. bhutanicum*, *Ge. bogoriense*, *Ge. gracile*, *Ge. oblongum*, *Ge. ozonioides*, *Ge. polymorphum*, *Ge. sinense*, and *Ge. tropicale*) (Arauzo and Iglesias 2014; Hustad 2015; Index Fungorum 2002–2024; this study). This study introduced four new species of *Geoglossum*, each morphologically distinct from these known species, with detailed morphological descriptions provided in Table 6. Similarly, *Trichoglossum* accommodates 30 legitimate species, with sequences published for fifteen species, and fifteen species lack effective sequences (Durand 1908b, 1921; Patouillard 1909; Sinden and Fitzpatrick 1930; Tai 1944; Cash 1958; Zhuang and Wang 1997; Kučera et al. 2010; Ekanayaka et al. 2017a; Prabhugaonkar and Pratibha 2017; Lee et al. 2021; de la Fuente et al. 2022; Hyde et al. 2023). We introduced six new species of *Trichoglossum* in this study, each showing morphological differences from known species, as outlined in Table 7.

**Table 7.** Comparison of *Trichoglossum* species.

Species	Ascomata	Ascigerous portion	Stipe	Paraphyses	Asci	Ascospores	References
*Trichoglossum ailaoense*	Spathulate, 3‒5 cm high, black, with setae	Inflated, flattened, usually vertically grooved, irregularly twisted, 0.2–0.4 × 0.5–1 cm	Cylindrical, up to 4.5 cm tall, about 1‒3 mm in diam.	Filiform, unbranched, apex swollen and hooked, septate, 2–3.5 μm, $\overset{ˉ}{x}$ = 172 × 2.9 μm, in middle, swollen part 4–7.5 μm, $\overset{ˉ}{x}$ = 5.1 μm, in diam., hyaline in the middle, light brown at base and apex	Narrowly cylindrical to broadly clavate, with rounded apex, and narrowed base, 200–250 × 14–25 μm, $\overset{ˉ}{x}$ = 218 × 19.4 μm, dark brown, 8-spored, amyloid	Clavate with rounded ends, 0–3-septate, (46.5–)49.5–67 (−69.5) × (4–)4.8–6.9(−7.6) μm, $\overset{ˉ}{x}$ = 59.7 × 5.8 μm, Q = 7.7–14.2, ***Q*** = 10.4 ± 1.07, dark brown	Hyde et al. (2023)
*T. benghalense*	Clavate, dumbbell or oval shaped, 2–6 × 20–35 mm, black, with setae	Flattened lanceolate to clavate	Flexuous and cylindrical	Filiform, septate, 3–6 μm wide in diam., light brown, with granulated wall	Clavate-cylindrical, obtuse, 127–227 × 13–20 μm, brown, 8-spored	Fusoid to clavate, 0–7-septate, 63–210 × 4–7 μm, brownish orange to dark brown	Chakraborty et al. (2022)
*T. caespitosμm*	Caespitose, clavate to spathulate, 18–30 × 4–10 mm, black, with setae	Glossoid, ellipsoidal, flattened, compressed, sometimes curved, 3–7 × 0.5–1 mm	Cylindrical, 1–33 mm long, up to 6 mm thick	Filamentous, septate, with wide, coiled to clavate, rugose apex, 16–28 × 3–8 µm	Cylindrical-clavate, obtuse, short-pedicellate at the base, 183–221 × 16–20 µm, hyaline, 8-spored, inamyloid	Filiform, narrowed and rounded at both ends, mostly 9–11-septate, 119–127 × 5–7 µm, brown to olivaceous	de la Fuente et al. (2022)
*T. cheliense*	Clavate, simple or bifurcated, 3–6 cm long, dark brown, with setae	Elliptic, rounded or forked, compressed, 5–14 × 5–10 mm	Terete to compressed, 30–45 × 1.5–3 mm	Hooked apex, brown or pale brown above, septate	Clavate, 200–231 × 18–22 µm, 8-spored	Clavate-cylindrical to subcylindrical, mostly 15-septate, rarely 13–14-septate, 89–151 × 5–6 µm, brown	Tai (1944)
*T. chuxiongense*	Cylindrical, 3.5–8.5 cm high	Flattened lanceolate or flexuous, obtuse, more or less compressed, 10–25 × 3–15 mm, black	Cylindrical, compressed, sometimes branch, 25–60 × 1.5–2.5 mm, black	Filiform, septate, with obviously swollen and curved apical portion, lower portion 1.5–4 µm, $\overset{ˉ}{x}$ = 2.5 µm in diam., 3.3–10.8 µm, $\overset{ˉ}{x}$ = 5.7 µm above, hyaline to light brown	Cylindrical-clavate, with croziers, 205–235(−240) × 17–26(−27) µm, $\overset{ˉ}{x}$ = 219 × 21 µm, 8-spored, amyloid	Filiform to clavate, 1–6-septate, mostly 6-septate, (61–)70–93(−104) × (4.1–)4.8–6.5(−7.2) µm, $\overset{ˉ}{x}$ = 83 × 5.6 µm, Q = 10.5–21.8, ***Q*** = 14.99 ± 1.65, brown	This study
*T. conica*	Clavate, 1–4.5 cm high, dark	Capitate, conical, or oval, mostly with tapered obtuse apex, irregularly dented, 4–10 × 3–8 mm	Cylindrical, straight to slightly slanted, 5–40 × 1–2.5 mm, dark	Filiform to cylindrical, multi-septate, with obviously swollen and curved apical portion, lower portion 1.5–4 µm, $\overset{ˉ}{x}$ = 2.5 µm in diam., apical cell 3.5–7.5 µm, $\overset{ˉ}{x}$ = 5.3 µm in diam., pale brown	Cylindrical-clavate, with croziers, (164–)168–203(−217) × (14–)18–29(−33) µm, $\overset{ˉ}{x}$ = 184 × 23 µm, 8-spored, amyloid	Filiform to cylindrical, mostly 8-guttulate, 5–7-septate, (86–)92–113(−117) × (5–)5.4–7.3(−8) µm, $\overset{ˉ}{x}$ = 103 × 6.3 µm, Q = 12.3–21.6, ***Q*** = 16.5 ± 1.67, dark brown	This study
*T. confusμm*	1.5–2.5 cm long, black when dry, with setae	Obovate	Terete, 10–20 × 1–1.5 mm	Apex slightly thickened, straight or curved, pale brown	Clavate, apically obtuse, 175 × 12 µm, 8-spored	Cylindric to clavate, mostly 7-septate, 55–73 × 4–5 µm	Durand (1921)
*T. distortus*	Clavate, 1.5–2.5 cm high	Flattened lanceolate, or swollen triangular, with severe central depressions, 3–6 × 3–5 mm	Cylindrical, 15–20 × 1.5–2.5 mm	Filiform, multi-septate, straight to slightly curved, with obviously curved, slightly swollen apical portion, lower portion 1.5–5.3 µm, $\overset{ˉ}{x}$ = 2.8 µm in diam., apical portion 2.5–6.5 µm, $\overset{ˉ}{x}$ = 4.4 µm in diam., brown or dark brown overall	Cylindrical-clavate, with croziers, (154–)161–189(−192) × 19–26(−28) µm, $\overset{ˉ}{x}$ = 179 × 24 µm, 8-spored, amyloid	Filiform, 10–16-septate, mostly 15-septate, (96–)101–129(−135) × (5–)5.4–7 (−7.4) µm, $\overset{ˉ}{x}$ = 114 × 6.2 µm, Q = 14.7–24.8, ***Q*** = 18.5 ± 1.93, mostly brown, sometimes light brown	This study
*T. farlowii*	Clavate, 2–6 cm long, black or brownish black, with setae	Lanceolate, compressed, 1–3 cm long, 3–5 mm or more thicker	Terete, often flexuous, 2 × 20–40 mm long	Cylindrical, septate, with curved to circinate, somewhat thickened tips, brownish above	Clavate, apex somewhat narrowed but rounded, 170–200 × 15–18 µm, amyloid, 8-spored	Clavate-cylindric, mostly 3-septate, rarely 1–5 or more, tapering very little or not at all above the middle, 48–85 × 6 µm, fuliginous or brownish	Durand (1908b)
*T. gracile*	Gregarious, 1–2 cm high, black, with setae	Clavate, ovate, obtuse, compressed, 1–3 mm long, 0.3–1.5 mm wide	Slender, filiform, flexuous	Filiform, slightly thickened at the top, dark brown above, septate	Clavate, 120 × 20 μm, amyloid, 8-spored	Fusiform-clavate, gradually tapering at both ends, 15-septate, 115–160 × 5–6 µm thick	Tai (1944)
*T. hirsutum*	Clavate to capitate, spathulate, 10–90 × 2–5 mm, black to brownish black, with setae	Compressed, 5–35 × 2–8 mm	Cylindrical, up to 20–50 mm tall, 1–3 mm thick	Filiform, curved or coiled at the apex, 2–6 µm in diam. in middle, 4–7 µm in diam. at the apex, below hyaline and above brown	Clavate, 180–300 × 17–35 µm, 8-spored	Fusoid-clavate, cylindric, fusiform, with acute base and rounded apex, 11–17-septate, mostly 15-septate, 80–170 × 5–7 μm	Prabhugaonkar and Pratibha (2017); Kaygusuz et al. (2020); Dasgupta et al. (2022); this study
*T. jejuense*	Tongue-shaped or fingerlike, 2–5 × 0.5–1.5 cm, dark brown to dark, with setae	Club-shaped, 1–2 cm tall, dark brown	Slender, 3–5 mm long, black	Filiform, tapered towards the tips, brown, septate	Cylindrical to club-shaped, 100–150 × 10–15 μm, amyloid, 8-spored	Elongate-fusiform, 50–70 × 3–4 µm, olive-brown, 15–16 septate	Lee et al. (2021)
*T. kunmingense*	1.5–3.5 cm long, without setae	Clavate, lanceolate to elliptical, compressed, 5.5–8 × 3–4 mm, brown	Terete, compressed, 2–3 mm thick, black	Hooked, septate, brown	Clavate, 175–225 × 19–25 µm, 8-spored	Clavate-cylindrical or sub-cylindrical, 7-septate, rarely 3–6-septate, 104–144 × 6–8 µm, brown	Tai (1944)
*T. octopartitμm*	Clavate, (20–)22–38(−39) mm high, black or grey-black, without setae	Flattened clavate to lanceolate, sometimes curved, occasionally vertically grooved, (8–)7–14(−15) × (2–)2.3–3(−4) mm	Cylindrical or flexuous and/or compressed, (10–)14–25(−27) × (1–)1.4–2.5 mm	Filiform, with somewhat enlarged, clavate, straight to hooked apex, septate, 1.5–2.5 μm in diam. in middle, apical cell 15–18 × 2.5–5.5 μm, hyaline	Cylindrical-clavate, narrowed below, with rounded apex, (185–)188–210(−228) × 17–21 μm, 8-spored, amyloid	Fusoid to fusoid-clavate, mostly 7-septate, (99–)101–125(−138) × 5–6 μm, brown	Kučera et al. (2010)
*T. persoonii*	2.5–4 cm long, black, without setae	Subglobose or ovate, with rounded apex, 5–9 × 3–8 mm	Terete, 10–30 × 1–2 mm, dark brown	Clavate, septate, with thickened, straight or curved above, brown	Clavate, 225–275 × 18–23 µm, 8-spored	Clavate-cylindrical, mostly 15–19-septate, rarely 13–20-septate, 162–200 × 5–6 µm, brown	Tai (1944)
*T. peruvianum*	Narrow-clavate to clavate-cylindrical, 1–2.5 cm long, fuscous black, with setae	Compressed, 1.5–2 mm in diam.	5–10 × 1 mm, black	Slender, septate, with erect or slightly recurved, clavate tips, inflated above to 5–6 μm in diam., subhyaline or pale brownish, hyaline below	Long-cylindrical, narrowed sharply at the apex and gradually towards the base, 120–130 × 8–11 µm, amyloid, 8-spored	Elongate-fusoid, broadest near the centre and narrowed towards both ends, often more acutely below, olivaceous, uniformly 7-septate, 50–75 × 2.5–3.5 µm	Cash (1958)
*T. qingchengense*	2–2.6 cm long, dark brown to black when dry, with setae	Broadly clavate, compressed, 7–9 × 3–4 mm	Somewhat compressed, 6–12 × 1.5–1.8 mm	Apex straight, curved, or circinate, slightly enlarged, 5–7.5 μm wide at apex, 3–4 μm wide in the lower portion	Clavate, 198–230 × 20–24 µm, amyloid, 8-spored	Subcylindrical, somewhat narrow at lower end, mostly 7–9-septate, rarely 6-septate, 76–117 × 6.5–7.7 µm, brown	Zhuang and Wang (1997)
*T. rasum*	Clavate to broadly spathulate, 15 cm high, black, with setae	Spatulate, lanceolate, strongly compressed, usually inciso-crenate, 15–50 × 5–20 mm	Terete, slender, cylindrical, flexuous, 2–5 mm thick	Filiform, with hooked, barely thickened tips, fuliginous	Cylindrical-clavate, 200–250 × 15–27 µm, amyloid, 8-spored	Clavate, one end abruptly pointed, mostly 5–9-septate, 93–140 × 5.6–6.5 µm, dark, brown	Patouillard (1909); this study
*T. rehmianum*	1.5–2.5 cm high when dry	Obovate, even or longitudinally furrowed. rather irregular, 1/3 to 1/2 the total length	Terete, 1–20 × 1–1.5 mm	Straight or curved, slightly thickened above, pale brown	Narrowly clavate, obtuse, 175 × 12 µm, 8-spored	Clavate-cylindrical, 3–7-septate, 55–73 × 4–5 µm, fuliginous	Durand (1908b)
*T. septatum*	Clavate, oval or dumbbell-shaped, slender, 18–35 × 2–4 mm, black	Flattened clavate to lanceolate, sometimes curved	Cylindrical or flexuous	Filiform, septate, apically enlarged and curved, 7–9 µm, $\overset{ˉ}{x}$ = 8.2 µm wide at the apices, light brown at the base, darker at the apex, granulate wall	Cylindrical-clavate, narrowed below, apex rounded, arising from croziers, 300–410 × 25–40 µm, $\overset{ˉ}{x}$ = 341 × 32 µm, 8-spored, amyloid	Fusoid to fusoid-clavate, 7- or 15-septate, 190–270 × 7–9 µm, $\overset{ˉ}{x}$ = 230 × 8.5 µm, greenish-brown to dark brown	Ekanayaka et al. (2017b)
*T. sinicum*	Clavate, 5.5–7 cm long, black	Lanceolate, compressed, 2.5–3 × 0.8–1 cm	Cylindrical, often slightly compressed and grooved, 30–40 × 3.5–5 mm	Clavate, septate, with brown tips	Clavate-cylindrical, 237–281 × 21–26 µm, 8-spored	Cylindrical-clavate, mostly 15-septate, rarely 7–15-septate, 147–175 × 6–7 µm, brown	Tai (1944)
*T. subhirsutum*	Capitate, 2.5–6 cm high	Obviously flattened lanceolate, sometimes irregularly folded, or triangular with severe central depressions, obtuse, compressed, 5–10 × 5–10 mm in diam.	Cylindrical, 20–50 mm in height, 1–3 mm in diam.	Filiform, septate, straight to slightly curved, with obviously swollen and curved apical portion, lower portion 1.3–3.3 µm, $\overset{ˉ}{x}$ = 2.1 µm in diam., apical cell 3–8 µm, $\overset{ˉ}{x}$ = 5.2 µm, light brown to brown	Cylindrical-clavate, with croziers, (195–)199–230(−232) × (16–)17–27(−30) µm, $\overset{ˉ}{x}$ = 213 × 21 µm, 8-spored, amyloid	Filiform, mostly 15-septate, rarely 2–10-septate, (100–)115–154(−164) × (4–)4.5–6.3(−7.6) µm, $\overset{ˉ}{x}$ = 137 × 5.4 µm, Q = 13.9–35.7, ***Q*** = 25.5 ± 3.53, light brown to dark brown	This study
*T. tetrasporμm*	3–8 cm high, black	Elliptical to sub-rotund, compressed, rounded above, not more than 1/5 the total length of the entire ascoma	Terete, flexuous, 1–2 mm thick, black	Cylindrical, somewhat curved, septate, with slightly thickened tips, 3 μm thick below, 7 μm thick above, smoky brown	Clavate, apically narrowed, 175–220 × 20–25 µm, 4-spored	Cylindrical, clavate, normally 15-septate, broadest above the middle, tapering each way to subobtuse ends, brown, 110–160 × 6–7 µm	Sinden and Fitzpatrick (1930)
*T. tropicale*	Clavate to capitate, 15–30 × 2–4 mm, black	Glossoid, ellipsoidal, flattened, sometimes curved, compressed, 2–4 mm × 1–2 mm thick	Cylindrical, 10–20 × 1 mm thick	Filamentous, septate, with capitate to bulbous terminal cells, 8–46 × 6–10 µm	Cylindrical to clavate, obtuse, short-pedicellate base, 155–180 × 16–18 µm, hyaline, inamyloid, 8-spored	Filiform, narrowed and rounded at both ends, mostly 10–12-septate, 122–132 × 5–5.5 µm, brown to olivaceous	de la Fuente et al. (2022)
*T. variabile*	Clavate to sub-capitate, 20–40 × 2–5 mm	Clavate to lanceolate or widely elliptic, compressed, generally vertically grooved, 6–13.7 × 2.4–4.8 mm, black or brownish black	Cylindrical, slender, occasionally compressed, flexuous, 11.9–32.9 × 1.1–2 mm, base up to 5 mm, slightly lighter colour than ascigerous portion	Filiform, septate, with enlarged, bend or convolute apex, 6–8 µm in diam. in middle, apical cell 17.8–27.1 × 3.7–6.2 µm, below hyaline, above dark brown	Cylindric-clavate, apex widely conical, tapered below, 150–226 × 18–20 µm, 8-spored	Subfusoid or fusoid-clavate, tapered at both the ends, more on one end, mostly 10–13-septate, rarely 4–16-septate, 80–150 × 4.5–6 µm, brown	Prabhugaonkar and Pratibha (2017); Dasgupta et al. (2022)
*T. velutipes*	Up to 10 cm high, black or brownish black	Lanceolate, elliptical or subrotund, compressed, obtuse, 3–15 × 4–10 mm	Terete, somewhat flexuous, 2–3 mm thick, black	Cylindrical, septate, with slightly thickened and curved or uncinate at the tips, pale brown	Clavate, short stipitate, apex narrowed, 175–210 × 18 μm, amyloid, 4-spored	Narrowed each way from above the middle, 8–11-septate, 115–166 × 6–7 µm, smoky brown	Durand (1908b)
*T. walteri*	3–7 cm or more high, brownish black, without setae	Narrowly elliptical to lanceolate, obtuse, compressed, 1–2 cm long, 3–5 mm or more wider	Terete or compressed, about 2 mm diam.	Cylindrical, septate, slightly thickened and curved at the tips, brown	Clavate, apex narrowed, obtuse, 175–200 × 18–20 µm, amyloid, 8-spored	Clavate-cylindrical, 82–107 × 6 μm, fuliginous or pale brown	Durand (1908b)
*T. wrightii*	Clavate, black, without setae	Irregular, about 1/3 the total length	About 2/3 the total length	Cylindric, slightly thickened and strongly curved apex, septate, pale brown above	Clavate-cylindric, 250–265 × 20–25 µm, 8-spored	Clavate, broadest above the middle, mostly 8–9-septate, rarely 5–7-septate, 105–145 × 7 μm, brown	Durand (1921)
*T. yunnanense*	3.5–7.5 cm, dark, without setae	Elliptical to subglobose, compressed	Terete, flexuous, 45–70 × 2–3 mm	Clavate, septate, with curved apex, brown	Cylindrical-clavate, 237–294 × 19–22 μm, 2–8-spored	Clavate to cylindrical, 15-septated, rarely 16-septated, 143–187 × 6–7 μm, brown	Tai (1944)

The topology of our phylogenetic trees differs from previous studies primarily due to two factors: the difference in taxon sampling and the different genes used in each study (Hustad and Miller 2015a; Fedosova et al. 2018; Fadnes et al. 2021; Kučera et al. 2021; Baba and Hirose 2023). Until 2015, the generic scope of *Geoglossaceae* had been defined to include nine genera: *Geoglossum*, *Glutinoglossum*, *Hemileucoglossum*, *Leucoglossum*, *Maasoglossum*, *Nothomitra*, *Sabuloglossum*, *Sarcoleotia*, and *Trichoglossum* (Hustad and Miller 2015a). Fedosova et al. (2018) constructed their ML tree using ITS, LSU, and the translation elongation factor 1 alpha (*TEF1*) genes for *Geoglossum*, *Glutinoglossum*, *Leucoglossum*, *Sabuloglossum*, *Sarcoleotia*, and *Trichoglossum*, but their study lacked sequences for *Hemileucoglossum*, *Maasoglossum*, *Nothomitra*, and *Sabuloglossum*. Baba and Hirose (2023) used ITS, LSU, *MCM7*, and *RPB1* to construct their ML phylogenetic tree of *Geoglossomycetes*, but they did not include sequences for *Nothomitra*. These differences in the taxon sampling and the genes used in the phylogenetic analyses caused differences between their phylogenetic trees and ours. Additionally, Hustad and Miller (2015a), Fadnes et al. (2021), and Kučera et al. (2021) constructed phylogenetic trees for *Geoglossomycetes* using ITS and LSU based on a larger number of genera, each with two or three species. In contrast, our phylogenetic trees are based on sequences from all legitimate species of *Geoglossomycetes*. We found that less than one-third of these taxa for our phylogenetic analyses had sequences for *MCM7*, *RPB1*, and *TEF1*. Therefore, we relied on ITS and LSU for our phylogenetic analyses, resulting in a different topology from other studies.

**Figure 16.**
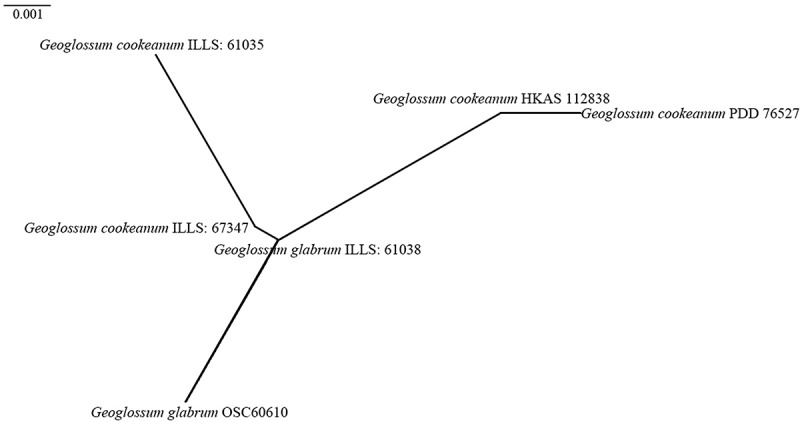
Results of the PHI test of *Geoglossum cookeanum* (PDD 76527, HKAS 112838, ILLS 67347, and ILLS 61035) and *Ge. glabrum* (OSC 60610 and ILLS 61038) using both LogDet transformation and splits decomposition. The PHI test results showed that the test did not find statistically significant evidence for recombination (*p* = 1.0).

Our phylogenetic analysis indicates that *Geoglossum cookeanum* (PDD 76527, HKAS 112838, ILLS 67347, and ILLS 61035) and *Ge. glabrum* (OSC 60610 and ILLS 61038) are closely related. Species boundaries can be defined using split analysis. The PHI results below 0.05 (*p* < 0.05) indicated significant recombination in the dataset, suggesting that they belong to one species, while the PHI results above 0.05 (*p* > 0.05) suggests that they belong to different species. Our PHI test based on ITS and LSU shows *p* = 1.0, which does not find statistically significant evidence for recombination. Therefore, our PHI test suggests that *Ge. cookeanum* (HKAS 112838 and PDD 76527) is one species, *Ge. cookeanum* (ILLS 61035 and ILLS 67347) and *Ge. glabrum* (ILLS 61038 and OSC 60610) belong to another species (Figure 16). Nannfeldt (1942) conducted a detailed morphological comparison between *Ge. cookeanum* and *Ge. glabrum*, and found distinct apical cells of paraphyses, surface characteristics of ascomata, and habitats, supporting the recognition of two separate species. However, subsequent researchers seem to have overlooked these differences, leading to inaccurate identifications. Therefore, in the future, we should conduct a more detailed morphological study of ILLS 61035 and ILLS 67347.

Our phylogeny showed that *Trichoglossum hirsutum* and *Geoglossum glabrum* are polyphyletic based on currently published sequences (Figure 1). Specimens of *T. hirsutum* from different collection sites, previously classified as the same species based on morphology, exhibit polyphyly. The specimens ILLS 67355 from the USA, ILLS 61045 from the Czech Republic, and HKAS 55133 from China (with Jamie Platt JP267 and OSC 61726 from unknown sites) clustered in separate sub-clades. Similarly, *Ge. glabrum* also shows polyphyly, with specimens ILLS 72358 from the Czech Republic and ILLS 61038 from the USA clustering in two separate clades. These findings suggest that the specimens may represent multiple cryptic species. In the future, researchers will need to obtain more samples of *T. hirsutum* and *Ge. glabrum* from different regions to validate this hypothesis through comprehensive studies involving morphology, phylogeny, and ecology. Additionally, one of our samples (HKAS 112858) and two specimens of *Glutinoglossum orientale* (LE<RUS>: 222166 and LE 291818) form a strong clade with 97% bootstrap and 1.00 Bayesian posterior probability. However, our sample is immature; therefore, we do not provide a detailed description or corresponding illustration in this study. *Glutinoglossum orientale* has currently only been reported in Russia, and may occur in China, which is a speculative guess (Fedosova et al. 2018). Future researchers could collect additional samples to verify this possibility.

## Supplementary Material

Additional file 1.jpg
